# Critical Evaluation of Polarizable and Nonpolarizable Force Fields for Proteins Using Experimentally Derived Nitrile Electric Fields

**DOI:** 10.1021/jacs.3c14775

**Published:** 2024-02-28

**Authors:** Jacob M. Kirsh, Jared Bryce Weaver, Steven G. Boxer, Jacek Kozuch

**Affiliations:** † Department of Chemistry, Stanford University, Stanford, California 94305-5012, United States; ‡ Experimental Molecular Biophysics, Department of Physics, Freie Universität Berlin, 14195 Berlin, Germany

## Abstract

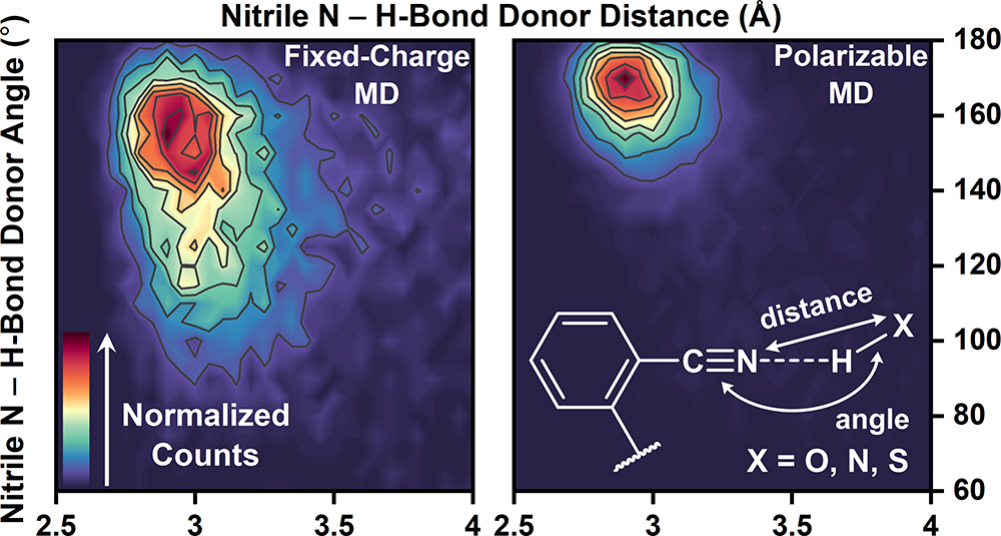

Molecular dynamics (MD) simulations are frequently carried out for proteins to investigate the role of electrostatics in their biological function. The choice of force field (FF) can significantly alter the MD results, as the simulated local electrostatic interactions lack benchmarking in the absence of appropriate experimental methods. We recently reported that the transition dipole moment (TDM) of the popular nitrile vibrational probe varies linearly with the environmental electric field, overcoming well-known hydrogen bonding (H-bonding) issues for the nitrile frequency and, thus, enabling the unambiguous measurement of electric fields in proteins (*J. Am. Chem. Soc.***2022**, *144* (17), 7562–7567). Herein, we utilize this new strategy to enable comparisons of experimental and simulated electric fields in protein environments. Specifically, previously determined TDM electric fields exerted onto nitrile-containing *o*-cyanophenylalanine residues in photoactive yellow protein are compared with MD electric fields from the fixed-charge AMBER FF and the polarizable AMOEBA FF. We observe that the electric field distributions for H-bonding nitriles are substantially affected by the choice of FF. As such, AMBER underestimates electric fields for nitriles experiencing moderate field strengths; in contrast, AMOEBA robustly recapitulates the TDM electric fields. The FF dependence of the electric fields can be partly explained by the presence of additional negative charge density along the nitrile bond axis in AMOEBA, which is due to the inclusion of higher-order multipole parameters; this, in turn, begets more head-on nitrile H-bonds. We conclude by discussing the implications of the FF dependence for the simulation of nitriles and proteins in general.

## Introduction

The organization of amino acids in proteins gives rise to local electrostatic environments which play pivotal roles in biological processes like protein folding,^1−3^ drug-target recognition and binding,^4−6^ and enzymatic catalysis.^5,7−9^ Given the importance of these processes, molecular dynamics (MD) simulations are frequently used to parse and quantify their underlying electrostatic contributions,^10−13^ which may then be used as design principles for novel systems.^14^ To ensure these simulations are reliable, computationally derived parameters need to be benchmarked against experimental results to improve the force fields (FFs) governing MD. Parameterizations of protein FFs often compare simulations with experimentally derived secondary and tertiary structures and NMR *J* coupling constants, among other observables;^15−17^ these comparisons are against either global or local structural properties, but not *local electrostatic* interactions. Vibrational Stark effect (VSE) spectroscopy interprets changes in the absorption of molecular vibrations through the influence of the local environmental electric field^4^ and therefore provides a direct comparison with the output of simulations. Nitriles (−C≡N) are popular vibrational probes^18−22^ whose VSE facilitates the measurement of the electric field projected along the −C≡N bond axis, *F*_C≡N_,^23^ and, as such, enables the quantification of the strength of noncovalent interactions using an electrostatic scale. Previous work demonstrated that nitrile frequencies of small molecules observed in aprotic solvation environments have a linear correlation with nitrile electric fields obtained from fixed-charge (FC) and polarizable (POL) MD simulations (called *F*_C≡N,FC MD_ and *F*_C≡N,POL MD_, respectively).^4,24^ This indicates that nitrile frequencies in aprotic environments can be evaluated within the framework of the VSE, and electric field-frequency calibrations enable the extraction of an electric field when a frequency is measured in a new environment, herein termed *F*_C≡N,FC FREQ_ or *F*_C≡N,POL FREQ_ depending on the FF used for calibration. Many studies attempted to extract *F*_C≡N,FREQ_ values for nitriles in protic solvents^25,26^ and more complex H-bonding environments in proteins (where nitriles were introduced via ligands and noncanonical or modified amino acids)^18,27−30^ for direct comparisons with *F*_C≡N,MD_ values in these environments. However, the comparisons were complicated by anomalous blueshifts in the nitrile frequencies due to H-bonding interactions which are not captured by the VSE frequency calibrations.^24,31−33^

**Figure 1:**
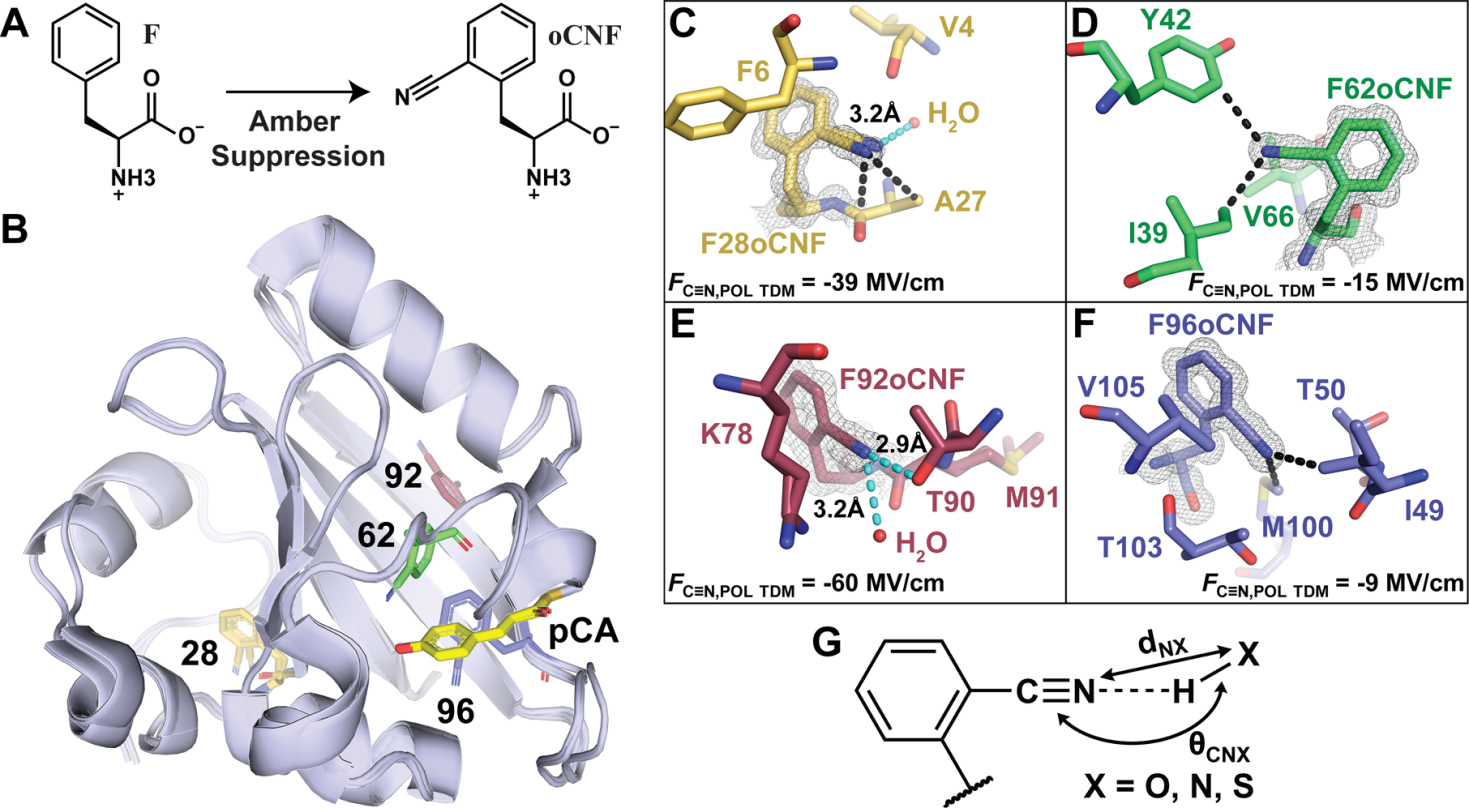
Overview of nitrile incorporation into PYP variants, their structural characterization, and nitrile H-bond geometric definitions. (A) Four native phenylalanines (F) were replaced with *o*-cyanophenylalanine (oCNF) via amber suppression. An overlay of the high-resolution crystallographic structures of each nitrile-containing PYP variant in (B) indicates the positions of each labeled nitrile site. oCNF residues are labeled by position and colored gold (F28oCNF PYP; PDB ID: 7SPX; resolution: 0.97 Å), green (F62oCNF PYP; 7SPW; 1.05 Å), red (F92oCNF PYP; 7SPV; 1.18 Å), and blue (F96oCNF PYP; 7SJJ; 0.95 Å).^24^ The PYP chromophore, p-coumaric acid (pCA), is displayed for each variant in yellow. 2mF_o_−DF_c_ electron density maps for F28oCNF (C), F62oCNF (D), F92oCNF (E), and F96oCNF (F) are shown contoured at 1σ along with local environments. *F*_C≡N,POL TDM_ values are shown in the bottom corners. Black dashed lines indicate hydrophobic interactions. Cyan dashed lines indicate potential H-bonds, where H-bonds are described in (G) by the distance from the −C≡N nitrogen to the heavy-atom donor (X; distance denoted *d*_NX_) and the −C≡N···X angle (θ_CNX_). H-bonds in (C) and (E) have *d*_NX_ < 4.0 Å on θ_CNX_ for consistency with definitions used in MD simulations (vide infra). Adapted with permission from ref (24). Copyright 2022 American Chemical Society.

In a recent report,^24^ we demonstrated that measured nitrile transition dipole moments (TDMs, i.e., the square root of the absorption intensities) vary linearly with electric fields *in both protic and aprotic solvation environments*, overcoming the issues observed for frequencies. Specifically, small molecule electric field-TDM calibrations remain robust when nitrile TDMs and MD electric fields for both aprotic solvents and water are included.^24^ This TDM-based VSE enables the extraction of *F*_C≡N,TDM_ values sensed by the −C≡N via the peak area (analogous to *F*_C≡N,FREQ_ values obtained via an electric field-frequency calibration), circumventing the interpretation issues for the nitrile frequency in H-bonding environments. What is more, a joint analysis of TDMs and frequency shifts enables quantification of the H-bonding blueshift.^24^ To demonstrate the applicability of using the TDM to extract *F*_C≡N,TDM_ values in proteins, we incorporated the nitrile-containing noncanonical amino acid *o*-cyanophenylalanine (oCNF) into photoactive yellow protein (PYP) via amber suppression^34^ at four native phenylalanine sites (positions 28, 62, 92, and 96) and obtained high-resolution crystal structures (Figure 1A,B).^24^ In the structures, F62oCNF and F96oCNF are in nonpolar environments, while F28oCNF and F92oCNF each possess at least one H-bond donor (defined by heavy-atom distances of <4.0 Å for consistency with definitions in MD simulations, vide infra; Figure 1C–G).^1^ Electric field-TDM calibrations were obtained for the model compound *o*-tolunitrile (oTN) with both FC and POL MD. Interpreting the protein −C≡N absorption intensities in IR spectra with the POL MD calibration revealed that the fields are as large as −60 MV/cm (F92oCNF) and as small as −9 MV/cm (F96oCNF; Table 1; Figure 1C–F).^2^ This range of 50 MV/cm is similar to the difference experienced for oTN in hexanes and water,^24^ highlighting the substantially different noncovalent interactions that occur within the protein.

**Table 1:** PYP Variants’ Nitrile TDM-Derived Electric Fields (*F*_C≡N,TDM_ Values) from Electric Field-TDM Calibrations of oTN with FC and POL MD

environment	*F*_C≡N,FC TDM_ (MV/cm)^a^	*F*_C≡N,POL TDM_ (MV/cm)^a^
F28oCNF	–23 ± 2	–39 ± 2
F62oCNF	–4 ± 2	–15 ± 2
F92oCNF	–38 ± 2	–60 ± 2
F96oCNF	1 ± 2	–9 ± 2

a Derived from values in ref (24).

In this study, we utilize this set of PYP variants to revisit the question of how accurately local electric fields are modeled within diverse protein environments by simulating the proteins with FC and POL MD FFs. We compare previously determined experimentally derived *F*_C≡N,TDM_ values^24^ with computationally derived *F*_C≡N,MD_ values and explore the structural and parametric rationales that underlie the observed (dis)agreements. Particular attention is paid to comparisons for F28oCNF and F92oCNF, the nitriles with crystallographic H-bond donors and substantial H-bonding blueshifts,^24^ whose electric fields were not experimentally assessable prior to the new TDM-based VSE.

## Results and Discussion

**Figure 2:**
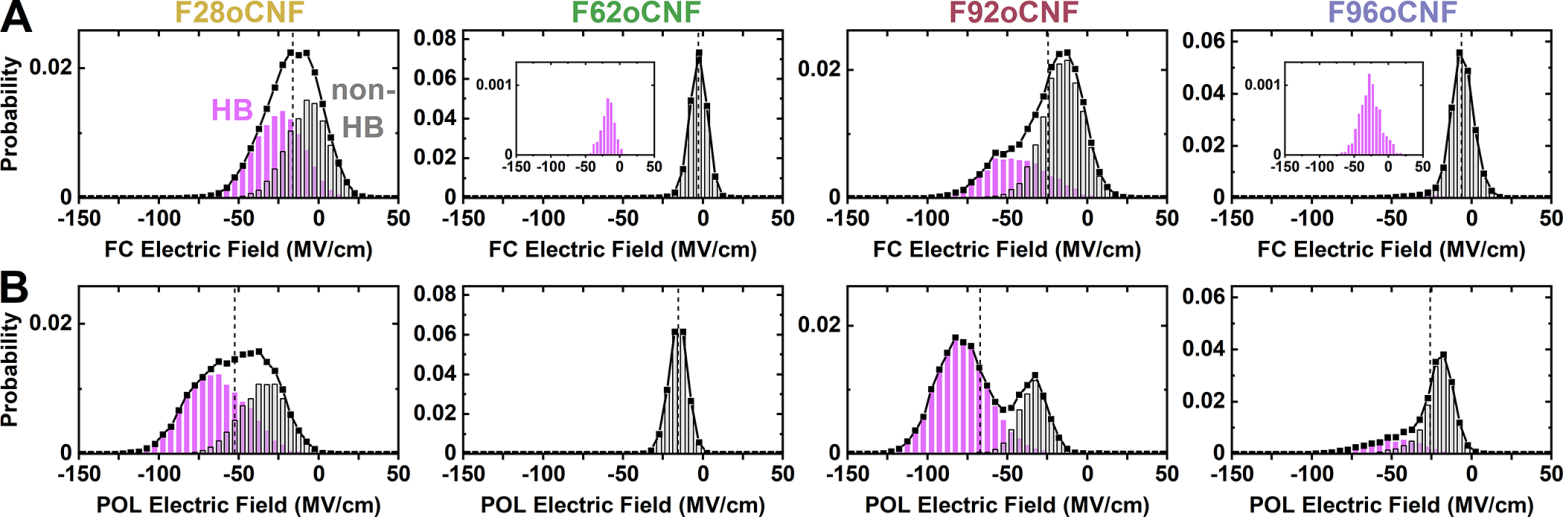
Calculated nitrile electric field (*F*_C≡N,MD_) distributions demonstrate FF dependence and straightforward deconvolution into H-bonding/non-H-bonding populations. Electric field distributions (black traces) are derived from FC MD (A) and POL MD (B) and are decomposed into their H-bonding (magenta) and non-H-bonding populations (gray). Note that the *y*-axes for different variants are on different scales. Insets show H-bonding populations magnified when this contribution is small. Dashed lines indicate the fraction-weighted *F*_C≡N,MD_ values.

MD simulations were performed with the FC AMBER ff99SB-ILDN FF^35^ and the POL AMOEBABIO18 FF,^17^ which differ in the extent of their electrostatic descriptions: while AMBER only considers atomic partial charges, AMOEBA uses charges, dipoles, quadrupoles, and polarizabilities on each atom to describe electrostatic potentials more accurately. Starting with high-resolution crystal structures (Figure 1C–F),^24^ we performed AMBER and AMOEBA simulations to produce 200 and 100 ns trajectories in aggregate per variant, respectively, and extracted *F*_C≡N,MD_ values every 10 ps (Figure 2 black traces; details for extraction of MD electric fields are provided in Section S1). The distributions of the variants’ electric fields demonstrate considerable variety in terms of shape, width, and center positions, and several distributions depend strongly on the choice of FF. Accordingly, we analyzed the origins of the MD *F*_C≡N_ distributions by characterizing the nitrile’s environment as H-bonding or non-H-bonding using a heavy-atom nitrile–H-bond donor–acceptor distance cutoff of 4.0 Å (*d*_NX_ in Figure 1G) and donor–acceptor–hydrogen angle cutoff of 30° (θ_NXH_ in Figure S2; see Figures S2 and S3 for details on H-bond cutoff choices). The non-H-bonding and H-bonding populations separate into distinct, nearly Gaussian electric field distributions that are centered at more positive and more negative *F*_C≡N,MD_ values, respectively (Figure 2 gray and magenta histograms; distributions with a 3.5 Å cutoff are effectively unaltered, shown in Figure S4), as previously observed for FC MD-based subpopulations of a nitrile-containing inhibitor for human aldose reductase.^29^ F62oCNF displays a symmetric MD electric field distribution, and it is accordingly involved in little to no nitrile H-bonding with both FFs (≤1.8%; Table 2). Only F96oCNF’s FC MD distribution is similarly symmetric, and it also had little H-bonding (3.5%; Table 2). All other electric field distributions exhibit either asymmetry or bimodality, and the corresponding nitriles experienced H-bonding in 23–74% of MD frames, with POL MD predicting up to 6.5 times higher H-bonding probability than FC MD (Table 2). Similar to the H-bonding fractions, the median H-bonding/non-H-bonding *F*_C≡N,MD_ values are sensitive to the FF. The median *F*_C≡N,POL MD_ values are consistently more negative than the *F*_C≡N,FC MD_ values by factors of 2–3 and 2–6 for the H-bonding and non-H-bonding populations, respectively (Table 2).

**Table 2:** Calculated Nitrile H-Bonding/non-H-Bonding Fractions, Median Electric Fields, Fraction-Weighted Electric Fields, and Associated Errors from Averaging Two (FC MD) or Four (POL MD) Trajectories^a^

environment/FF	H-bonding fraction (%)	non-H-bonding fraction (%)^b^	H-bonding median *F*_C≡N,MD_ (MV/cm)	non-H-bonding median *F*_C≡N,MD_ (MV/cm)	fraction-weighted *F*_C≡N,MD_ (MV/cm)^c^
F28oCNF/FC	51.5 ± 1.1	48.5	–24.8^d^	–6.9 ± 0.3	–16.1 ± 0.3
F28oCNF/POL	59.8 ± 5.4	40.2	–64.9 ± 1.6	–34.3 ± 1.8	–52.6 ± 0.3
F62oCNF/FC	1.8 ± 1.8	98.2	–16.3^e^	–2.7 ± 0.4	–3.0 ± 0.2
F62oCNF/POL	0	100	N/A	–15.4 ± 1.0	–15.4 ± 1.0
F92oCNF/FC	31.6 ± 3.8	68.4	–44.5 ± 0.6	–15.4 ± 1.1	–24.6 ± 0.2
F92oCNF/POL	74.0 ± 10.6	26.0	–78.6 ± 1.9	–33.5 ± 1.0	–67.0 ± 5.0
F96oCNF/FC	3.7 ± 2.4	96.5	–25.3 ± 1.9	–5.8 ± 0.2	–6.5 ± 0.3
F96oCNF/POL	22.6 ± 7.5	77.4	–49.1 ± 1.5	–19.5 ± 0.4	–26.1 ± 1.8

a An explanation for the origin of the errors is provided in Section S1**.**

b Same error as for the H-bonding fraction.

c Sum of the products of the H-bonding/non-H-bonding fractions and the H-bonding/non-H-bonding median *F*_C≡N,MD_ values.

d Error <0.1 MV/cm.

e No error could be calculated because only one trajectory had H-bonding.

While the MD electric field distributions demonstrate variety in their shape, the observed room temperature IR spectra of all variants were well-fit with a single, symmetric band (spectra shown in Figure S11),^24^ indicating an apparent discrepancy between the calculations and experiment. Since the asymmetries/bimodalities for F28oCNF, F92oCNF, and F96oCNF’s calculated electric field distributions arise from the presence of both H-bonding and non-H-bonding nitrile populations (Figure 2), one possible explanation is that these species are in chemical exchange and that they exchange quickly enough to appear as a single IR band at room temperature. Indeed, linear IR spectra of a nitrile experiencing varying molecular environments–rapid exchange between H-bonding and non-H-bonding interactions, in this case—can demonstrate a single band or multiple bands depending on how quickly the electric fields fluctuate in comparison with the nitrile’s dephasing lifetime.^36,37^ To address this possibility, we analyzed the nitrile’s H-bonding state and electric fields as functions of time in both FC and POL MD (Figure S5) and determined the lifetimes of the H-bonding and non-H-bonding states (Figures S6 and S7; Tables S6 and S7). We then employed a two-state first-order exchange model to describe the transition between these states and found that the exchange rate between the protic and aprotic nitrile populations is >0.1 ps^–1^ (Table S8). This rate translates to a lifetime of <10 ps, which is comparable to the time scale of vibrational dephasing for *p*-cyanophenylalanine (∼4 ps;^38,39^ no lifetime could be found for oCNF) such that a single symmetric IR lineshape is feasible. In addition, the H-bonding/non-H-bonding lifetimes from the exchange model can be used to predict the nitrile H-bonding fractions (Table S11). The exchange model correctly predicts POL MD has more H-bonding, and the results deviate from the H-bonding fractions in Table 2 by an RMSD of only 11%, suggesting that the length of our MD simulations was sufficient to approach equilibrium statistics of the nitriles’ environments.

**Figure 3:**
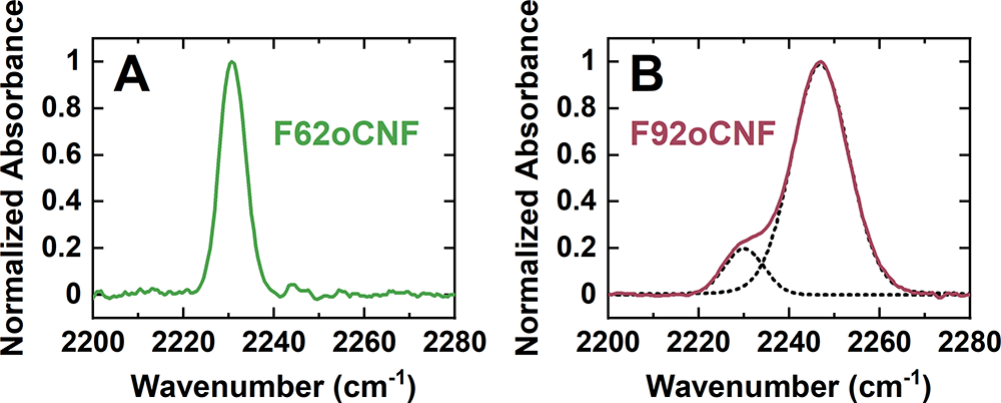
Experimental low-temperature (100 K) FTIR spectra reveal a single nitrile population for F62oCNF but two populations for F92oCNF. Protein samples were buffer exchanged into glass forming 1:1 mixtures of glycerol and aqueous buffer to enable low-temperature studies. (A) F62oCNF’s spectrum displays a single band centered at 2230.8 cm^–1^. At room temperature in the buffer, a single band was also observed (Figure S11).^24^ (B) F92oCNF’s spectrum displays two bands (2230.1 and 2246.9 cm^–1^ for minor and major peaks, respectively; fits are shown as black dotted lines). In contrast, the room temperature spectrum contains a single band at 2241.3 cm^–1^ (Figure S11).^24^ The apparent disparity in the number of IR populations at low and room temperature can be explained by rapid chemical exchange between the species at room temperature, as predicted by simulations. IR spectra for F62oCNF and F92oCNF (in addition to F28oCNF and F96oCNF) at additional temperatures up to 323 K can be found in Section S5.

In order to experimentally test the possibility of multiple underlying and exchanging populations, we acquired an FTIR spectrum for F92oCNF at 100 K in frozen solution (Figure 3B). We also acquired a low-temperature spectrum for F62oCNF (Figure 3A) as a control since the room temperature IR TDM analysis revealed the nitrile is entirely non-H-bonding,^24^ consistent with the MD simulations (Table 2). As predicted, F62oCNF’s spectrum remains as a single band (central frequency of 2230.8 cm^–1^; Figure 3A; Table S12). In contrast, F92oCNF’s spectrum displays one dominant peak at 2246.9 cm^–1^ with a well-resolved shoulder centered at 2230.1 cm^–1^ (Figure 3B; Table S12). By comparing F62oCNF’s frequency with the frequency of F92oCNF’s bluer band, it is clear that this F92oCNF population must be H-bonding to attain such a transition energy. Additionally, the similarity of F92oCNF’s shoulder frequency to F62oCNF’s frequency coupled with the MD results suggests that the shoulder represents a non-H-bonding population. Low-temperature IR spectra were also obtained for F28oCNF and F96oCNF (Figure S12). Like F92oCNF, those nitriles demonstrated bandshapes indicative of multiple populations, though band fitting was not as straightforward as in F92oCNF’s case. Ultimately, the presence of multiple species at low-temperature for F28oCNF, F92oCNF, and F96oCNF (but not F62oCNF) together with the above exchange rate analysis provides strong evidence that the difference in the MD electric field distributions and the room temperature IR arises from a rapid chemical exchange between H-bonding and non-H-bonding nitrile populations. What is more, since the transition energy for exchanging populations appears at the population-weighted value,^40^ our findings also imply that the MD H-bonding/non-H-bonding median *F*_C≡N,MD_ values should be averaged for comparison with *F*_C≡N,TDM_ values.

**Figure 4:**
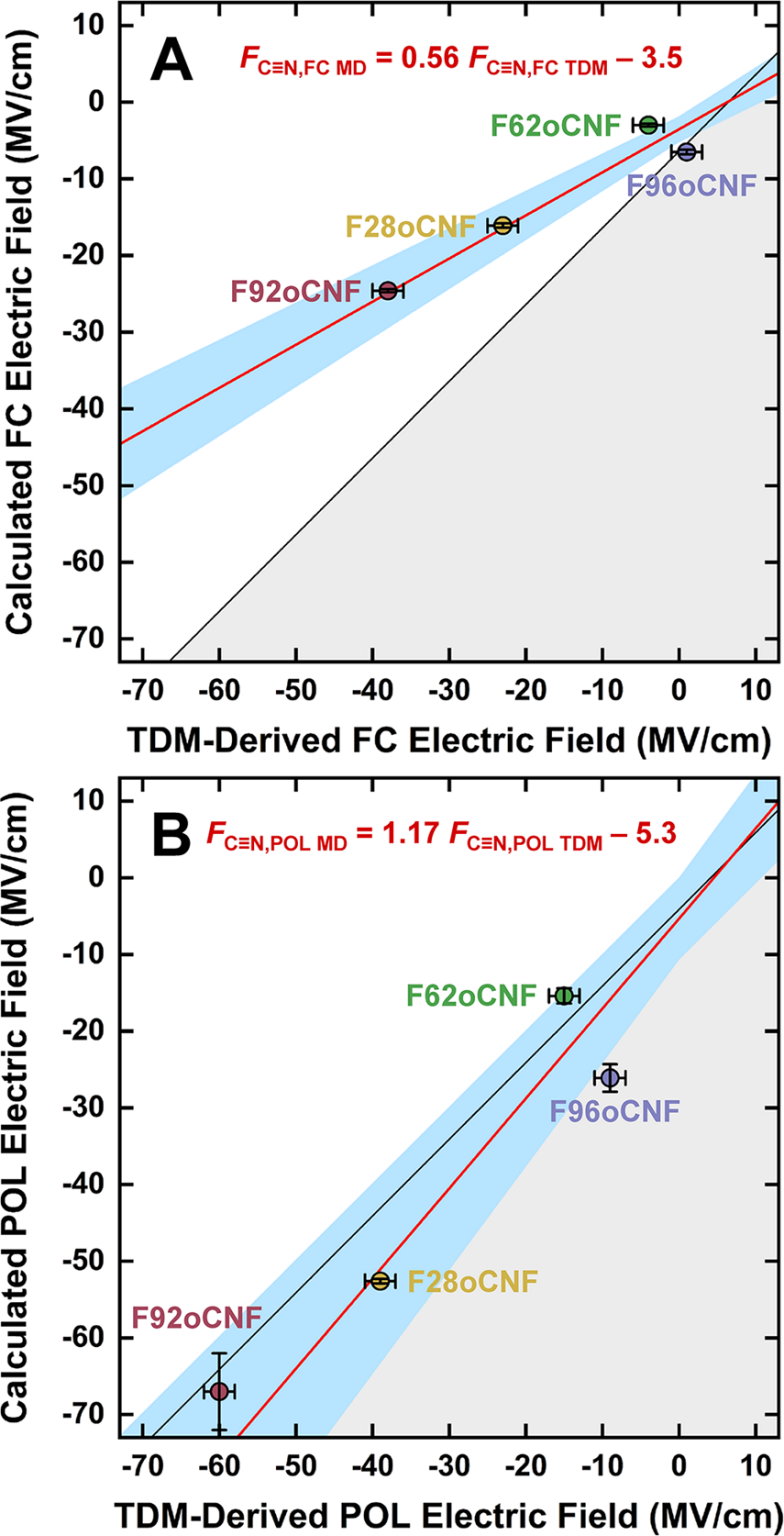
Comparisons of fraction-weighted *F*_C≡N,MD_ and *F*_C≡N,TDM_ values for PYP variants indicate POL MD can better recapitulate TDM-derived electric fields than FC MD. *F*_C≡N,TDM_ values are derived from oTN-based electric field-TDM calibrations using either FC (A) or POL (B) MD;^24^*F*_C≡N,MD_ values come from MD of proteins using the same FFs. Black lines represent perfect agreement between electric fields derived from TDMs and MD simulations: they have unit slope and are shifted from the diagonals due to offsets arising from the imperfect transferability of the small molecule oTN calibrations to the case where oCNF is incorporated into the protein (i.e., *F*_C≡N,FC MD_ = *F*_C≡N,FC TDM_ – 6.4 and *F*_C≡N,POL MD_ = *F*_C≡N,POL TDM_ – 4.1, see Section S1 for details). Points in the gray area below the black line have overestimated *F*_C≡N,MD_ values, while points in the white area above the line have underestimated *F*_C≡N,MD_ values. Weighted linear regressions^45,46^ are shown in red, and their equations are *F*_C≡N,FC MD_ = (0.56 ± 0.04) *F*_C≡N,FC TDM_ – (3.5 ± 0.8) (A) and *F*_C≡N,POL MD_ = (1.17 ± 0.09) *F*_C≡N,POL TDM_ – (5.3 ± 2.7) (B). The blue-shaded regions indicate 2σ CIs for regressions.

In order to examine which FF describes the TDM-derived electric fields more accurately, we determined fraction-weighted *F*_C≡N,MD_ values (Table 2; values with a 3.5 Å H-bond cutoff are nearly identical, see Table S4). The electric field magnitudes have the qualitative trend of F96oCNF ≈ F62oCNF < F28oCNF < F92oCNF for both FFs, which is similar to the ordering of the *F*_C≡N,TDM_ values (Tables 1 and 2). However, the *F*_C≡N,POL MD_ values are consistently larger than the *F*_C≡N,FC MD_ values by up to ∼−40 MV/cm and by factors as big as 5 (a much smaller change in *F*_C≡N,FC MD_ values and *F*_C≡N,POL MD_ values was observed for oTN in solvents, see Figure S19 and discussion). As such, when we quantitatively compare the correlations between *F*_C≡N,FC TDM_/*F*_C≡N,FC MD_ values and *F*_C≡N,POL TDM_/*F*_C≡N,POL MD_ values, substantial differences are observed depending on the FFs (Figure 4). TDM-derived and MD-derived electric fields for F62oCNF and F96oCNF are in reasonable agreement with those of both FFs. In contrast, an analogous comparison for F28oCNF and F92oCNF, the two nitriles with significant H-bonding in both FC and POL MD, suggests the TDM-derived fields are better recapitulated with POL MD. Weighted linear regressions for comparisons using FC MD and POL MD have slopes of 0.56 ± 0.04 and 1.17 ± 0.09, respectively (Figure 4), a significant approximately 2-fold difference; the regressions have intercepts of −3.5 ± 0.8 and −5.3 ± 2.7, an insignificant difference. In considering the 2σ confidence intervals (CIs) for the fits (blue-shaded regions in Figure 4), the correlation of *F*_C≡N,FC TDM_ and *F*_C≡N,FC MD_ values falls markedly outside the ideal correlation with a slope of 1 (black lines in Figure 4; note the lines have y-intercepts of −6.4 and −4.1 for FC MD and POL MD, respectively, which represent the offsets due to the imperfect transferability of oTN’s TDM-field calibrations as a noncovalent species to oCNF, which is covalently linked to the protein; see Section S1 for details), whereas the CI for *F*_C≡N,POL TDM_ and *F*_C≡N,POL MD_ values indicates a good match between experiments and MD simulations (Figure 4). The best-fit line and CI for *F*_C≡N,FC TDM_ and *F*_C≡N,FC MD_ values suggest the FC FF substantially underestimates large electric fields. A related result was documented in calculations of the electric field experienced by a functionally important carbonyl group at the active site of the enzyme ketosteroid isomerase,^41−43^ where the field is <−100 MV/cm.^7,44^ Our results suggest that such underestimation can already occur at moderate electric field strengths of <−10 MV/cm; thus, POL MD is more capable of properly sampling the variants’ diverse noncovalent interactions and environments.

**Figure 5:**
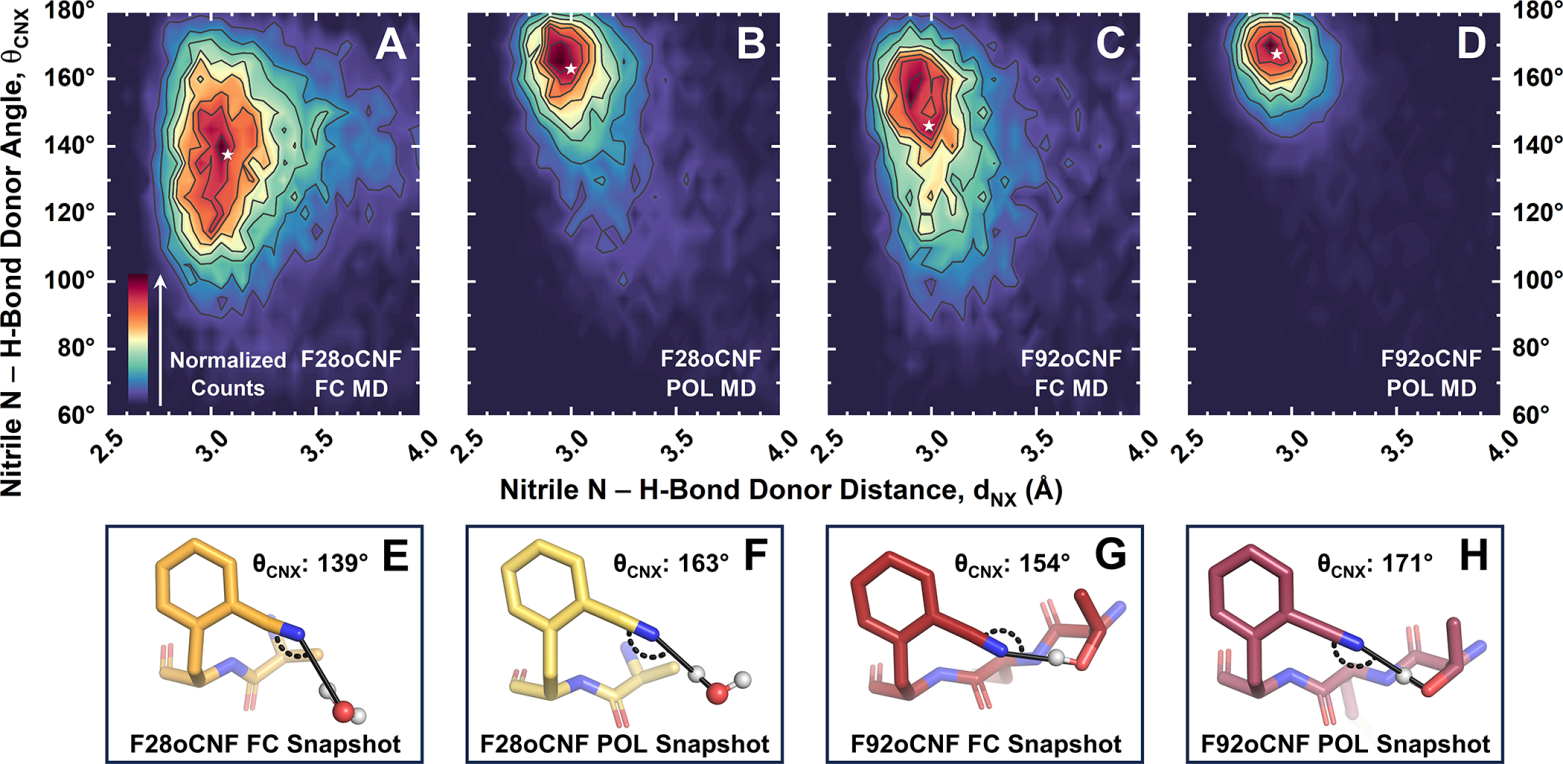
Nitrile H-bond sampling is highly sensitive to FF. A broader distribution of H-bond distances (*d*_NX_) and angles (θ_CNX_) are sampled for F28oCNF and F92oCNF with FC MD (A/C) than with POL MD (B/D), and average H-bond distances and angles decrease and increase with POL MD, respectively (average positions marked with a star; Table S13). Counts in each panel are normalized. Snapshots of F28oCNF H-bonding with water at geometries with the average θ_CNX_ and *d*_NX_ values from FC and POL MD are shown in (E) and (F), respectively. In analogy, representative snapshots at the average H-bond distance and angle for F92oCNF–T90 H-bonds from FC and POL MD are shown in (G) and (H), respectively (Table S13). The snapshots illustrate the FFs’ impacts on H-bonding. Note that an alternate T90 rotamer was also observed in FC MD which adopts very similar average θ_CNX_ and *d*_NX_ values (Figure S27; Table S13). In (E–H), carbon is shown in gold (F28oCNF PYP) or red (F92oCNF PYP), nitrogen is shown in blue, oxygen is shown in bright red, and protons are shown in white.

Since the largest differences in fraction-weighted *F*_C≡N,FC MD_ and *F*_C≡N,POL MD_ values occurred for F28oCNF and F92oCNF, and specifically for the H-bonding populations (Table 2), we analyzed the nitrile H-bond geometries (i.e., the H-bond angles θ_CNX_ and distances d_NX_ shown in Figure 1G) those variants sampled in FC and POL MD (Figure 5A–D; see also Figures S20–S23). The contour plots in Figure 5A–D are strikingly different: the H-bond angles and distances have a much narrower distribution in the POL MD centered at shorter distances and larger angles. These observations are supported by fits with Gaussian surfaces: for both variants, average *d*_NX_’s are shorter by up to 0.1 Å in POL MD (3.08 Å vs 3.00 Å for F28oCNF; 2.99 Å vs 2.93 Å for F92oCNF; for AMBER vs AMOEBA, respectively); at the same time, the H-bonding is better aligned along the −C≡N axis (138° vs 163° for F28oCNF; 146° vs 167° for F92oCNF; Table S13). Additionally, the width of the θ_CNX_ distributions is 1.4 times wider in FC MD for F28oCNF and 2.5 times wider for F92oCNF (Table S13). The fits indicate the H-bond angle sampling is particularly sensitive to the FF. Furthermore, nitriles in POL MD adopt closer, more head-on H-bonds, which aligns with chemical intuition given that the nitrile’s lone pair of electrons reside along the −C≡N axis.

To evaluate the origin(s) of the changes to the contour plots, we identified whether the nitrile H-bonds were with the protein or the solvent (Table S15) and assessed whether the FF affected this. We found that F28oCNF’s predicted H-bond donor identity is not sensitive to the FF while F92oCNF’s is. F28oCNF H-bonds with the solvent 91–95% of the time in both FFs, indicating that the changes to the contour plots are due to altered water geometries around the nitrile (representative snapshots are shown in Figure 5E,F). In contrast, F92oCNF H-bonds with the protein 50% of the time in FC MD but 95% of the time in POL MD (Table S15). More specifically, F92oCNF H-bonds with T90’s hydroxyl, the closest crystallographic H-bond donor (Figure 1E),^24^ in 41% of the FC MD H-bonding frames compared to 90% for POL MD, a larger than 2-fold difference (Table S15). Since F28oCNF–water H-bond sampling was FF-dependent, we investigated the geometries of F92oCNF’s H-bonds with either water or T90 to see if altered sampling contributes to the differences in Figure 5C,D in addition to the changes in H-bond donor identities. The average H-bond distance and angle for F92oCNF H-bonding with water in FC MD (Figure S24) are 3.07 Å and 129°, quite similar to the values for F28oCNF (Table S13). Unfortunately, we cannot provide a comparison with POL MD due to the sparsity of H-bonding. Focusing on the F92oCNF–T90 H-bond, this interaction in FC MD has an average θ_CNX_ value of 154° (and average *d*_NX_ values of 2.91–2.95 Å for two observed rotamers, shown in Figure 5G and Figure S27; see Figure S25 for contour plots; Table S13). The fit to the contour plot for F92oCNF–T90 H-bonds in POL MD (Figure S26) shows that the average H-bond distance is the same (2.93 Å) as in FC MD but the average angle is larger (170°; Figure 5H), indicating an analogous change to the H-bond angle as for F28oCNF (Table S13). However, F28oCNF predominately H-bonds with the labile solvent, while F92oCNF and T90 are covalently linked, making the change in the F92oCNF–T90 H-bond angle particularly striking. This analysis indicates that both H-bond partner fractions and H-bond geometries can be altered by the FF, as in the case of F92oCNF, and that in silico descriptions of nitrile H-bonding can be affected regardless of donor identity.

**Figure 6:**
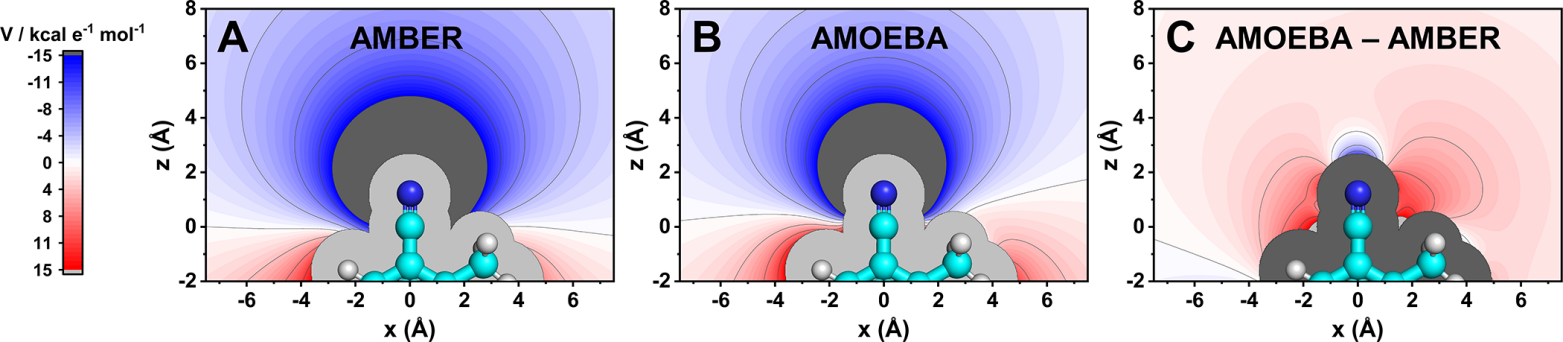
ESP maps for the oCNF model compound oTN indicate that negative charge density is more concentrated along the −C≡N axis in the AMOEBA FF than the AMBER FF. (A) and (B) are the ESP maps for oTN in the *xz* plane (Figure S28) with the AMBER and AMOEBA FFs, respectively, where negative and positive potentials are colored blue and red, respectively. The −C≡N C atom is located at the origin. The difference map between the AMOEBA and AMBER FFs (C) shows a more negative potential, or more negative charge density, at positive *z* values with zero *x*-component, i.e., along the −C≡N bond axis. Additionally, a positive potential is observed at intermediate angles in the *xz* plane, i.e., not along the −C≡N bond axis. In a chemical sense, these differences indicate the nitrile’s electron density is more focused along the bond axis, specifically in front of the N atom in the AMOEBA FF, while negative charge density is more diffusely distributed around the nitrile in the AMBER FF (analogous results are observed in the *yz* plane in Figure S29). Further, ESP difference maps between AMBER or AMOEBA with QM indicate substantially closer agreement between AMOEBA and QM (Figures S30 and S31), further validating the AMOEBA potential distributions.

The consistently larger nitrile average H-bond angles observed in POL MD versus FC MD motivated us to consider how the nitrile is described in both FFs. The AMOEBA FF possesses two significant changes to the electrostatic description from FC MD, i.e., multipoles and polarizability, so we used the model compound oTN to assess whether the electrostatic potential (ESP) maps around the nitrile reflect these differences (Figure 6). We observed that the negative potential around the N atom is narrower in the off-axis directions of the nitrile bond in AMOEBA compared to AMBER (Figure 6A,B). This is more clearly shown in the difference map for the ESPs between the AMOEBA and AMBER FFs, showing more negative potentials located along the −C≡N bond axis and more positive potentials at angles off the bond axis (Figure 6C). This indicates that the inclusion of dipoles and quadrupoles in the AMOEBA FF leads to a nitrile with more negative charge density concentrated in front of the −C≡N axis, modeling the nitrile’s lone pair, while the purely monopolar nitrile description with the AMBER FF results in a nitrile with a more angularly diffuse negative charge distribution. These results rationalize the strong FF dependence of the average H-bond angles for F28oCNF and F92oCNF’s H-bonding populations: more negative potentials along the nitrile bond axis in POL MD leads AMOEBA to a more head-on modeling of the nitriles’ H-bond donors’ O–H bond dipoles, while more negative potentials off the nitrile bond axis in FC MD leads AMBER to model those same interactions at smaller θ_CNX_’s.

The investigation of the nitriles’ H-bonding behavior in MD led us to consider the FF-dependent H-bond sampling of other moieties in the simulations. We characterized the H-bond geometries for backbone carbonyls that either participate in secondary structural elements or are located on loops (Figures S32–S41 and Section S9 for details). We found that the average carbonyl H-bond angles and distances (with angles and distances now defined as θ_COX_ and *d*_OX_, respectively, in analogy to the definitions in Figure 1G) in beta sheets are largely invariant to the FF, while the values for carbonyls in alpha helices are *smaller* and *longer* with POL MD than FC MD (Table S16), the opposite of what was observed for nitrile H-bonds (Table S13). For carbonyls on loops, comparing the contour plots in Figures S39–S41 indicates that a broader range of d_OX_’s are sampled in POL MD, and the POL MD distributions are consequently worse fit by Gaussian surfaces (Table S16). This initial assessment suggests backbone carbonyls are a good target for future benchmarking, particularly since their H-bonding is a ubiquitous feature of proteins and their electric fields can be inferred via the VSE on the frequencies.^4,9,47^

## Conclusions

In summary, enabled by the new TDM-based VSE analysis, we overcame the issues of nitriles as vibrational electric field probes and directly compared experimentally derived and MD-based electric fields (*F*_C≡N,TDM_ and *F*_C≡N,MD_ values, respectively) in H-bonding protein environments. Better agreement was observed with the POL AMOEBA FF than with the FC AMBER FF: this is justified by the inclusion of higher order multipoles in AMOEBA, which led to more ordered and head-on H-bonding geometries consistent with stronger solvation electrostatics. Nitriles are commonly found on drugs,^48^ and since electrostatic interactions like H-bonding influence ligand binding,^4,6^ our results suggest that computational screens of nitrile-containing compounds with methods like docking would be benefitted by using AMOEBA,^49^ albeit at a higher computational cost. More broadly, this work complements previous electric field tests of nitriles and carbonyls. The nitrile in F62oCNF participates in almost no H-bonds and the MD-derived and TDM-derived electric fields match well with both FFs, consistent with observations that FC MD could recapitulate nitrile and carbonyl electric fields in aprotic protein environments.^4,26^ In contrast, we observed that *F*_C≡N,MD_ values for nitriles in H-bonding environments need POL MD to successfully recapitulate *F*_C≡N,TDM_ values, consistent with Welborn and Head-Gordon’s demonstration that AMOEBA was necessary to recapitulate a carbonyl electric field in an H-bonding environment like the active site of ketosteroid isomerase.^43^ The electric fields experienced by H-bonded nitriles as studied herein are likely to be encountered routinely in and around proteins, unlike the bespoke fields found at enzyme active sites, bringing into question the necessary and sufficient conditions to accurately sample protein environments in silico. Performing POL MD has traditionally come at a substantial computational expense, but new force fields and platforms to run them are constantly under development to ameliorate cost while maintaining (or improving) accuracy.^50−52^ Recent advances in the OpenMM and Tinker platforms which run the AMOEBA FF allow for integration of GPUs,^53−55^ for example, which enabled our simulations (and others)^22,56,57^ to progress well into the ns regime. Since these technological advances make simulations of proteins with POL MD more feasible, there is an increased need for benchmarking to determine the contexts in which POL MD becomes essential. This work provides some guidance toward that goal, and in future work, further engineered local perturbations to the nitriles’ environments will be reported and analyzed within the framework outlined here to continue exploring AMOEBA’s capabilities.

## Supporting Information Available

Materials and methods on MD simulations with the AMBER and AMOEBA force fields, ESP map calculations, calculation of MD electric fields, calculation of nitrile H-bonding/non-H-bonding residence times, and temperature-dependent FTIR spectroscopy; additional discussion on assessment of the oCNF “self-field,” the transferability problem, nitrile H-bond cutoffs, characterization of MD electric fields and H-bond fractions, time-dependent MD quantities, nitrile H-bonding/non-H-bonding lifetime analysis, first-order exchange model, characterization of temperature-dependent IR, correlation of FC and POL MD electric fields, characterization of nitrile H-bonding in MD, additional oTN ESP maps, MD carbonyl H-bonding characterization, and assessment of the variants’ structural stability throughout the MD simulations (PDF)

## Abbreviations

MD: molecular dynamics
FF: force field
VSE: vibrational Stark effect
FC: fixed-charge
POL: polarizable
TDM: transition dipole moment
oCNF: *o*-cyanophenylalanine
oTN: *o*-tolunitrile
d_NX_: heavy-atom nitrile H-bond distance
θ_CNX_: nitrile H-bond angle
*F*_C≡N, FC MD_: electric field obtained from fixed-charge MD
*F*_C≡N, POL MD_: electric field obtained from polarizable MD
*F*_C≡N, FC FREQ_: electric field extracted using a frequency-field calibration with fixed-charge MD
*F*_C≡N, POL FREQ_: electric field extracted using a frequency-field calibration with polarizable MD
*F*_C≡N, FC TDM_: electric field extracted using a TDM-field calibration with fixed-charge MD
*F*_C≡N, POL TDM_: electric field extracted using a TDM-field calibration with polarizable MD.

## Footnotes

1 “FXXoCNF” (where XX = 28, 62, 92, or 96) is used as shorthand for “the nitrile of FXXoCNF.”

2 By convention, a negative electric field indicates an energetically stabilizing interaction.^4^
