# Supplementary material for: Critical Evaluation of Polarizable and Nonpolarizable Force Fields for Proteins Using Experimentally Derived Nitrile Electric Fields
Source: J Am Chem Soc. 2024 Feb 28;146(10):6983–91. doi: 10.1021/jacs.3c14775 (PMC10941190; doi:10.1021/jacs.3c14775)
Supplement: Supplementary file 1 — ja3c14775_si_001.pdf [file ja3c14775_si_001.pdf]

# Supporting Information for

# "Critical Evaluation of Polarizable and Nonpolarizable Force Fields for Proteins using Experimentally-Derived Nitrile Electric Fields"

Jacob M. Kirsh,[1] Jared Bryce Weaver,[1,#] Steven G. Boxer,[1,*] and Jacek Kozuch[2,*]

[1]Department of Chemistry, Stanford University, Stanford, California 94305, United States.

[2]Department of Physics, Freie Universität Berlin, D-14195 Berlin, Germany

[#]Present Address: Drug Discovery, Insitro, Inc. 279 E. Grand Ave., South San Francisco, California 94080, United States.

**Table of Contents**

**S1 Molecular Dynamics (MD) Simulations and Analysis** .......... S3

Starting Geometries for Molecular Dynamics (MD) Simulations of PYP Variants .......... S3

Fixed-Charge MD Calculations .......... S3

Polarizable MD Calculations .......... S3

Parameterization of o-Cyanophenylalanine Sidechain and p-Coumaric Acid-Cysteine 69 Covalent Adduct S3

Electrostatic Potential (ESP) Map Calculations .......... S4

Calculation of MD-based Electric Fields .......... S4

Assessment of the oCNF "Self-Field" when Incorporated into PYP for Comparisons of Experimental, VSE-based and MD-based Electric Fields .......... S5

The Transferability Problem: Issues About Using the oTN TDM-Field Calibration for oCNF .......... S7

Calculation of Nitrile Non-H-Bonding/H-Bonding State Residence Times .......... S7

Origin of Errors Associated with MD-Derived Quantities .......... S8

Data Availability .......... S8

**S2 Temperature-Dependent FTIR Spectroscopy** .......... S9

**S3 Characterization of MD Electric Fields for Entire Distributions and H-bonding/non-H-bonding Populations** .......... S10

MD Electric Field and Experimental IR Distributions .......... S10

Defining Nitrile H-Bond Geometric Cutoffs .......... S11

H-Bonding/Non-H-Bonding Populations .......... S14

**S4 Assessment of Time-Dependent MD Quantities** ........ S16
Time-Dependence of Electric Fields and H-Bonding ........ S16
Non-H-Bonding/H-Bonding State Residence Times ........ S17
Fitting Non-H-Bonding/H-Bonding State Lifetimes ........ S20
Two-State First Order Exchange Model of Nitrile H-Bonding ........ S22
Predicting H-Bonding Fractions from the Exchange Model ........ S25
Assessment of the H-Bonded vs Non-H-Bonded Nitrile Vibrational Stark Tuning Rates ........ S26
**S5 Temperature-Dependent FTIR Spectra Characterization** ........ S28
PYP Variants' Room Temperature Spectra in Buffer ........ S28
100 K IR Spectra for F28oCNF and F96oCNF ........ S28
IR Spectra Obtained Between 100 K and 323 K ........ S30
Characterization of Temperature-Dependent IR Spectra ........ S35
**S6 FC MD vs POL MD Electric Field Correlations** ........ S36
**S7 MD Nitrile H-Bonding Characterization** ........ S37
Nitrile H-Bond Contour Plots ........ S37
Nitrile H-bond Distances and Angles Extracted from Contour Plots ........ S44
MD H-Bond Donor Identities ........ S46
Average MD Structures for F92oCNF H-Bonding with T90 ........ S47
**S8 oTN Electrostatic Potential (ESP) Maps** ........ S48
**S9 MD Carbonyl H-Bonding Characterization** ........ S50
Carbonyl H-Bond Contour Plots ........ S51
Carbonyl H-bond Distances and Angles Extracted from Contour Plots ........ S61
**S10 Assessment of the Variants' Structural Stability During MD Simulations** ........ S62
**S11 References** ........ S64

## S1 Molecular Dynamics (MD) Simulations and Analysis

### *Starting Geometries for Molecular Dynamics (MD) Simulations of PYP Variants*

MD simulations for F28oCNF, F62oCNF, F92oCNF, and F96oCNF Photoactive Yellow Proteins (PYP) were started from X-ray crystal structures with PDB IDs 7SPX, 7SPW, 7SPV, and 7SJJ, respectively,[1] and performed and analyzed as described previously.[1–4]

### *Fixed-Charge MD Calculations*

Fixed-charge (FC) protein MD simulations were performed using the ff99SB-ILDN force field (FF)[5] with GROMACS 2020.[6] The proteins were placed in the center of a cubic box with 7.5 nm long edges, filled with TIP3P water and NaCl at a concentration of 50 mM such that the system is net charge neutral. The systems were minimized (steepest descent algorithm until all forces were below 1000 kJ $mol^{-1}$ $nm^{-1}$), equilibrated using several NVT and NPT ensembles for 500 ps each in periodic boundary conditions (first all heavy atoms restrained, then $C_\alpha$ atoms, and no restrains in the final NPT). The velocity-rescaling thermostat and Berendsen barostat were used at 300 K and 1 bar with cutoffs set to 1.2 nm, long-range electrostatics treated with Particle Mesh Ewald, and LINCS restraints on X-H atoms (where X are heavy atoms). MD production runs used the Parrinello-Rahman barostat and were performed under NPT conditions, with time steps of 1 fs. Each system was simulated two times for 100 ns, generating a 200 ns aggregate MD trajectory for each protein variant. An assessment of the PYP variants' structural stability over the course of the simulations is provided in **Section S10**.

### *Polarizable MD Calculations*

Polarizable (POL) protein MD simulations were performed with Tinker9[7] using the AMOEBABIO18 FF,[8] and simulations were performed in an analogous manner to the FC simulations. A cubic box with 7.5 nm edges was filled with water and NaCl at 50 mM and minimized (steepest decent until all forces were below 1 kcal $mol^{-1}$ $Å^{-1}$) with electrostatics and van der Waals cutoffs of 7 and 9 Å, respectively. The dipole convergence criterion was set to 0.01 D with a mutual polarization scheme. During NVT and NPT equilibrations (restraints as specified for FC MD), the van der Waals cutoff was increased to 12 Å, and the RESPA integrator, the Bussi thermostat, and Monte-Carlo barostat (300 K and 1 bar) were employed. MD production runs were performed using a similar setting as in the NPT equilibration with 1 fs time steps for a total time of 25 ns. Runs were repeated four times to produce 100 ns aggregate MD trajectories for each variant. An assessment of the PYP variants' structural stability over the course of the simulations is provided in **Section S10**.

### *Parameterization of o-Cyanophenylalanine Sidechain and p-Coumaric Acid-Cysteine 69 Covalent Adduct*

Within the simulations, two different "noncanonical" amino acids needed to be parameterized: nitrile-containing *o*-cyanophenylalanine (oCNF) and *p*-coumaric acid (pCA) covalently bound to Cys 69 (*i.e.,* the chromophore for PYP).[9,10] Initial parameters for the capped oCNF amino acid and the capped pCA-Cys69 amino acid adduct were generated using AmberTools18[11] to be compatible with the general AMBER force field[12] (with

AM1-BCC charges) using a structure optimized with density functional theory (b3lyp/6-311++g**) in Gaussian16.[13] To incorporate the new residues into the ff99SB-ILDN FF,[5] the backbone partial charges were changed to match those of phenylalanine or cysteine, respectively, and a correct overall charge of zero for the amino acids was ensured by minimal adjustment of the partial charges of the $C_\beta$ and $H_\beta$ atoms. These parameters were appended into the original ff99SB-ILDN FF such that the PDB2GMX module in GROMACS automatically generated input files for the MD simulations. An analogous strategy was followed to obtain AMOEBA parameters. First, the capped amino acids were parameterized using Poltype2[14] (electrostatic parameters were based on MP2/6-311++G** calculations). Then, parameters of the backbone were set to match the ones in the AMOEBABIO18 FF.[8] With backbone electrostatic parameters fixed, the remaining electrostatic parameters and torsion parameters were refined using the POTENTIAL function in TINKER 8[15] and using Poltype2, respectively. Note that the PDBXYZ function of TINKER (which converts TINKER XYZ files to standard XYZ files) did not recognize the new amino acids, so the oCNF and pCA-Cys residues were manually changed to Phe and Cys in the PDB structures, respectively, as input for PDBXYZ and then converted back to the oCNF and pCA-Cys amino acids.

### *Electrostatic Potential (ESP) Map Calculations*

Electrostatic potential (ESP) maps of *o*-tolunitrile (oTN) as shown in main text **Figure 6** and **Figures S29-S31** were generated using the POTENTIAL function of TINKER 8[15] with a grid size of 0.1 Å. Overall, a strategy was adapted which is utilized in Poltype2[14] to generate electrostatic parameters. Accordingly, quantum mechanical maps were based on an optimized structure using the MP2/6-311++G** level of theory and calculated with Gaussian16.[13] The keyword "density=MP2" was used to obtain the electrostatic potential as output in the _.chk file, which was translated into a formatted _.fchk file using Gaussian's FORMCHK utility (command: formchk _.chk _.fchk). Next, using Gaussian's CUBEGEN program, a manually generated grid file (grid size of 0.1 Å) was used to obtain a _.cube file with electrostatic potentials on the plane specified by the grid (command: cubegen 0 potential=mp2 _.fchk _.cube -5 h < _.grid), which served as input for TINKER's POTENTIAL utility (option 2) providing the _.pot file displayed as ESP maps. ESPs for FC MD were generated by transferring AMBER electrostatic parameters into the TINKER format (polarizabilities, dipoles and quadrupoles were set to zero), and AMOEBA parameters were used as obtained from Poltype2.[14] The QM-based_.pot file was used as template for the POTENTIAL utility (option 5) to obtain the AMBER and AMOEBA ESP maps.

### *Calculation of MD-based Electric Fields*

MD-based electric fields ($F_{C≡N,MD}$'s) were determined as the mean electric field between the C and N atoms of the nitrile group along the –C≡N bond axis. In GROMACS, full coordinates and forces at the C and N atoms were saved every 10 ps for analysis, and the electrostatic forces of the entire system acting on the –C≡N group were obtained by, first, determining the sum of all forces and, second, recalculating the forces with all partial charges set to zero (using the -rerun keywork in mdrun). The difference resulted in the electrostatic forces, which were divided by the atomic partial charges of C and N to yield the atomic electric field vectors. These were dotted into the unit –C≡N bond vector to produce scalar electric fields, which were then averaged to produce the reported $F_{C≡N,FC\ MD}$ values.

In TINKER, the induced dipoles on the C and N atoms were saved every 10 ps and were divided by their polarizability parameters to provide the atomic electric fields; these values were again dotted into the unit –C≡N

bond vector and averaged to produce the reported $F_{C≡N,POL\ MD}$ values. Note that the same procedures were used to extract electric fields for oTN in FC and POL MD.[1]

*Assessment of the oCNF "Self-Field" when Incorporated into PYP for Comparisons of Experimental, VSE-based and MD-based Electric Fields*

In this work, we report absolute MD electric fields on the –C≡N group and do not correct for *intramolecular* contributions to the electric fields (referred to loosely as a "self-field")[16] from the oCNF side chain. We do this because we are considering a *covalent* probe molecule, where the way to define the self-field is not as obvious as the case of *noncovalent* probe molecules used in our previous work[1] and where attempts to remove it are likely to introduce systematic errors. The validity of this approach is supported by the small electric fields obtained for one estimate of the self-fields and the fact our conclusions are drawn from the changes in the *slopes* in main text **Figure 4**, not the changes in the *intercepts*. The following discussion expounds on these topics.

**Self-field for noncovalent ligand VSE probes**: The VSE for TDMs (to first order) is[1]

$$\vec{m} = \vec{m}_{ref} - \underline{A} \cdot \vec{F}$$

where $\vec{m}$ is the TDM, $\vec{F}$ is the electric field, $\underline{A}$ is the transition polarizability, and $\vec{m}_0$ is the TDM in its reference state. When calibrating a probe's electrooptic parameters via solvatochromism,[17] the reference state is defined where the probe experiences no *intermolecular (environmental)* electric field, *i.e.*, the probe is in vacuum. In vacuum, while no *environmental* electric field is present, the probe still experiences an *intramolecular* electric field: this field is referred to as the "self-field." In this work, where the (nitrile of the) small molecule oTN was used for the TDM-field solvatochromic calibration, oTN in vacuum therefore defines the experimental reference state, referred to as the "*zero-field*" condition. When the *zero-field* TDM and the transition polarizability are known, the *environmental* electric field can be determined by measuring a TDM in an environment of interest.

In MD simulations, the most straightforward electric field to extract is the *absolute* electric field (*i.e.*, the field due to the electrostatic forces exerted by *all* atoms in the simulation box). The MD *absolute* electric field contains the contribution from *intramolecular* and *intermolecular* interactions, while electric fields measured by experiments report only on the *intermolecular* interactions. Therefore, to maximize consistency between TDM-derived and MD-derived electric fields, an MD assessment of the field from *intramolecular* interactions (the self-field) is needed. The self-field can be determined (i) via an *in vacuo* MD simulation of the molecule of interest which serves as the *zero-field* reference or (ii) by rerunning MD simulations with a neutralized environment to obtain the electrostatic forces on the probe due to the ligand itself.[1,16] When the self-field is removed from the *absolute* MD electric fields, the *environmental* fields are obtained. Previous calculations used these strategies to determine *environmental* MD electric fields in solvents, and these MD fields were used in frequency-field or TDM-field solvatochromic calibrations for noncovalent species.[1–3,16,18]

**Self-field of covalent adduct VSE probes and inherent limitations**: The above approach works well for noncovalent probes because there is an intuitive distinction between the system (the probe molecule) and the surroundings (the environment). For nitriles introduced into the PYP variants, the –C≡N probe is introduced as part of the covalently bound oCNF residue. This covalency makes calculations of the self-field experienced by the –C≡N less straightforward, since the distinction between system and surroundings is less intuitive. We see two reasonable ways the self-field could be approximated in this case:

1. To remain consistent with our previous TDM-field calibration,[1] oTN's self-field can be used as a proxy for oCNF's self-field. The advantage of this approach is that it is a direct extension of the strategy for noncovalent probes. However, differences between oTN's and oCNF's FF parameters will not be accounted for and will therefore introduce an unphysical offset in the MD electric fields, suggesting the strategy for assessing self-fields in noncovalent probes does not transfer well to the case of covalent probes.

2. Another choice is to "cut" oCNF at a specific bond to create an artificial system-surroundings distinction. Logical cut sites would be at the $C_\beta$-$C_\gamma$ bond or at the $C_\alpha$-$C_\beta$ bond, where the latter choice would "recreate" the oTN molecule (**Figure S1**). By choosing a cut site, the self-field of the severed side chain could serve as the zero-field reference, or the electrostatic parameters could be switched off according to an appropriate strategy. However, one must consider that AMBER and AMOEBA FFs are designed according to specific exclusion principles, meaning that 1-2 and 1-3 interactions are excluded and 1-4 interactions are scaled during the calculation of electrostatic forces.[8,19] Additionally, AMOEBA introduces polarization groups separated by rotatable single bonds which can mutually induce dipoles between (but not within) groups via atomic polarizabilities.[8] So, cutting at a bond will alter the AMBER and AMOEBA parameters from what were used to produce the MD trajectories, and the remaining electrostatic parameters will create a fictitious electric field.

Unfortunately, we find that both approaches have limitations and may introduce errors on a scale that is (at least) of a similar magnitude as the self-field value we are trying to evaluate. As such, the schemes to evaluate the self-field for covalent probes carry with them intrinsic issues that will be observed as systematic offsets to $F_{C\equiv N,MD}$ values. Therefore, we chose not to explicitly correct the calculated $F_{C\equiv N,MD}$ values with a self-field value and instead to work with the *absolute* MD electric fields.

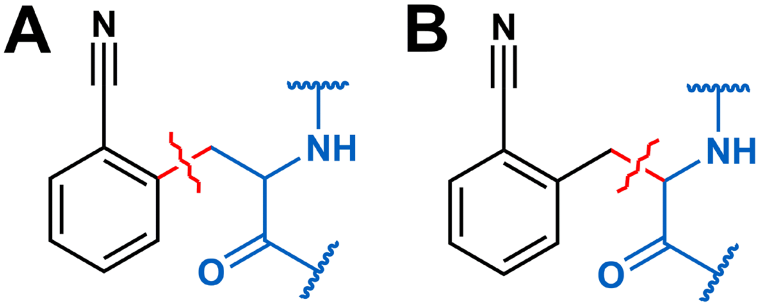


**Figure S1.** oCNF molecule with cuts at the (A) $C_\beta$-$C_\gamma$ or (B) $C_\alpha$-$C_\beta$ bond.

**Further justification for the choice not to apply a self-field correction to oCNF's $F_{C\equiv N,MD}$'s**: Even though we decided not to apply a self-field correction to the MD electric field values, we were still curious to test one of the two proposed self-field methods for covalent probes to estimate the self-field's magnitude. We chose the first strategy, and we performed *in vacuo* MD simulations for oTN under NVT conditions for 10 ns without periodic boundary conditions. The simulations yielded average AMBER and AMOEBA electric fields for oTN's intramolecular interactions with its –C≡N group of +6.4 and +4.1 MV/cm, respectively. These are small values compared to the magnitudes of the MD electric fields for H-bonding nitriles F28oCNF and F92oCNF, and they are on the order of the accuracy of the methods we employed for calculating electric fields, suggesting that leaving the $F_{C\equiv N,MD}$'s unaltered does not fundamentally alter our conclusions.

In addition, we note that the AMOEBA FF defines the cyanophenyl group of oCNF as one polarization group (*i.e.*, it *cannot* induce dipoles on the –C≡N), and the $C_\beta$ and beyond as further polarization groups that *can* induce a dipole on the –C≡N. Since the cyanophenyl group cannot polarize itself and we determine the $F_{C≡N,MD}$'s via the induced dipoles for POL MD, there is an "intrinsic" self-field correction in this case. As such, further corrections may not be necessary (at least for the POL MD electric fields), and they may even compound $F_{C≡N,MD}$ with additional errors.

Finally, we conclude by reiterating that the self-field is a *constant* offset to the MD-derived electric fields of *all* PYP variants; these offsets would affect the intercepts in main text **Figure 4**, but not the slopes, which are the basis for our most significant conclusions.

## *The Transferability Problem: Issues About Using the oTN TDM-Field Calibration for oCNF*

In this study, we use the TDM-field calibration for oTN and directly apply it to measurements of nitriles on oCNF. This begs the question: "are the electrooptic parameters calibrated for oTN the same as for oCNF in the protein setting?" Since oCNF is covalently incorporated into the protein while oTN was calibrated as a noncovalent species in solvents, changes to the parameters are plausible: we call this issue the "transferability problem." In considering this problem, we do not expect the changes to be large given the distal location of oCNF's covalent amide bonds relative to the nitrile. The changes to oTN's vs oCNF's electrooptic parameters would manifest as slightly different TDM-derived electric fields for measurements with oCNF; for simplicity, we approximate any change as a constant offset applicable to all PYP variants. In the last subsection, we detailed how we chose to use *absolute* electric fields (*i.e.*, we did not remove the oCNF self-field), which introduces an offset to the MD-derived electric fields compared with what is experimentally measured (*i.e.*, *environmental* fields). We estimated the offset from the transferability problem – and in reality from both the transferability problem and the self-field, since both create offsets – with the (negative of the) oTN self-field values described in the last subsection (since removing any offset requires its negation). We were motivated to estimate the value in this way by considering the *intramolecular* interactions in oTN that may be altered when oCNF is a covalent adduct. To assess our estimates, we compared the (negative of the) self-fields with the intercepts of the linear regressions in main text **Figure 4**. The intercepts are empirical assessments of the offsets between the experimentally- and computationally-derived nitrile electric field values. In FC MD, the intercept is -3.5 MV/cm and the self-field correction is -6.4 MV/cm; in POL MD, the intercept is -5.3 MV/cm and the self-field correction is -4.1 MV/cm. The values are quite similar in both cases, suggesting that our self-field estimates adequately account for the subtle transferability problem in this case.

## *Calculation of Nitrile Non-H-Bonding/H-Bonding State Residence Times*

MD-based nitrile state residence times were determined by calculating how long the nitrile maintained a non-H-bonding/H-bonding state before switching to a H-bonding/non-H-bonding state. MD coordinates were saved every 10 ps (and as such, the time resolution for this analysis is 10 ps), and from these a given timepoint could be classified as H-bonding (with respect to the nitrile) or non-H-bonding (subject to the distance and angular constraints described in the subsection "*Defining Nitrile H-Bond Geometric Cutoffs*"). Starting with the first timepoint (or any timepoint at which the state had flipped), the residence time was defined as "0 ps." Then, the subsequent timepoint's nitrile state was checked. If the subsequent timepoint was in a different state, the

residence time was reset to "0 ps" since the switch occurred faster than we could resolve with our 10 ps temporal resolution; if instead the same state was observed, the residence time was defined as "10 ps," and the next timepoint was checked. This process continued until a timepoint was found in which the state had flipped. In that event, if the state flipped at timepoint *x*, the residence time from the *x-1*$^{th}$ timepoint was used as the total residence time in that particular state. The residence time from the *x-1*$^{th}$ timepoint was recorded into a master list which cataloged the final residence time after a state flipped and whether the state had been H-bonding or non-H-bonding. Then, the residence time for timepoint *x* was redefined as "0 ps," and the process began anew. This process was repeated until every timepoint's state had been checked. The master list was split into two lists depending on the state, and the residence times in each of the two lists were histogrammed to determine how frequently a particular residence time was observed throughout the aggregate trajectories. Plots of residence time vs counts for both nitrile states are displayed in **Figures S6-S8**.

### *Origin of Errors Associated with MD-Derived Quantities*

Two or four independent replicates were performed for FC and POL MD simulations, respectively. Quantities of interest such as H-bonding fractions and median electric fields were quantified for each replicate. For each variable, the average value across the replicates is used as the reported value, and the errors are the standard deviation in the variable across the replicates.

### *Data Availability*

All parameters and Python scripts used in this work (for the extraction of MD electric fields and H-bonding related quantities, in addition to the generation of average MD structures) can be found at:

https://github.com/KozuchLab/Publications/tree/main/oCNPhe_GROMACS_TINKER

## S2 Temperature-Dependent FTIR Spectroscopy

Details for PYP expression and purification can be found in our previous publication.[1] F28oCNF, F62oCNF, F92oCNF, F96oCNF, or WT PYP were exchanged into a buffer containing 20 mM Tris HCl (Fisher Scientific) and 10 mM NaCl (Fisher Scientific) at pH 8.0. PYP was concentrated on 0.5 mL 10 kDa Amicon centrifugal filter units to approximately 20 µL, at which point it was further concentrated for an additional hour at 14,000 G at 4°C. Samples were transferred to Eppendorfs and the volume was recorded. An equal volume of glycerol was added to bring the samples to 50:50 aqueous buffer:glycerol (v/v), at which point each sample was spun down for one hour at 21,000 G at 4°C.

The protocol for sample assembly and temperature-dependent IR measurements was adapted from ref [20]. Two 3 mm thick calcium fluoride ($CaF_2$) windows were preconditioned (*i.e.*, washed) with methanol, and approximately 17 µL of sample was drop cast by pipette onto one window and sandwiched by the other window with 25 µm and 50 µm Teflon spacers in between. The assembly was inserted into a custom-built copper cell and fastened into place by tightening four screws on the copper cell; care was taken not to overtighten the screws to ensure the windows would not crack upon dunking the assembly into liquid nitrogen (described below). The copper cell was attached to the bottom of the cold finger of a Janis non-immersion, continuous-flow cryostat (a Lake Shore ST-100 Series by the current nomenclature), with a thin indium piece in between to promote thermal conduction.

The cold finger harboring the sample was dunked into a dewar of liquid nitrogen and held there until bubbling subsided to bring the sample to 77 K. The cold finger was slotted into the cryostat (which was inserted into a Bruker Vertex 70 spectrometer via an attached base plate), and a pump was attached to the cryostat to bring the sample under vacuum. A transfer line attached to a dewar (Janis) filled with liquid nitrogen was inserted into the cold finger and the line opened to blow liquid nitrogen over the sample, and a pump was attached to create a vacuum down through the cold finger. A Lakeshore 330 autotuning temperature controller was attached to a thermocoupling port from the cryostat to set the desired temperature. Over the temperature range used (100 K to 323 K), the transfer line opening and the strength of the pump attached to the cold finger were adjusted to assist with expedient thermalization. The temperature of the sample reached equilibrium at a set value with minimal fluctuations (< 0.2°C).

Spectra were recorded at eight temperatures: 100 K, 123 K, 160 K, 200 K, 233 K, 283 K, 303 K, and 323 K. Each spectrum resulted from averaging 512 scans, and 1 $cm^{-1}$ resolution was used. Spectra were acquired for all nitrile containing PYPs, and spectra were acquired for WT PYP to serve as a blank.

## S3 Characterization of MD Electric Fields for Entire Distributions and H-bonding/non-H-bonding Populations

*MD Electric Field and Experimental IR Distributions*

**Table S1.** Average and median calculated nitrile electric fields for fixed charge (FC) and polarizable (POL) MD simulations.

| PYP Construct | Average $F_{C\equiv N,FC\ MD}$ (MV/cm) | Median $F_{C\equiv N,FC\ MD}$ (MV/cm) | Average $F_{C\equiv N,POL\ MD}$ (MV/cm) | Median $F_{C\equiv N,POL\ MD}$ (MV/cm) |
|---|---|---|---|---|
| F28oCNF | -16.7 | -15.7 | -52.6 | -51.4 |
| F62oCNF | -3.1 | -2.8 | -15.6 | -15.4 |
| F92oCNF | -24.6 | -21.0 | -66.6 | -71.3 |
| F96oCNF | -7.5 | -6.0 | -27.3 | -22.3 |

**Table S2.** Standard deviations (s.d.'s) of FC and POL MD nitrile electric field distributions.

| PYP Construct | s.d. $F_{C\equiv N,FC\ MD}$ (MV/cm) | s.d. $F_{C\equiv N,POL\ MD}$ (MV/cm)[a] |
|---|---|---|
| F28oCNF | 16.9 | 22.5 |
| F62oCNF | 5.7 | 5.8 |
| F92oCNF | 20.0 | 24.6 |
| F96oCNF | 8.6 | 17.0 |

[a] All POL MD s.d.'s are larger than the analogous FC MD values. This is the opposite of what was found in simulations of an electric field at the active site of peptidyl-prolyl isomerase cyclophilin A, where electric field distributions with AMBER and CHARMM (FC FFs) were broader than with AMOEBA (POL FF).[21]

**Table S3.** Standard deviations (s.d.'s) of experimental nitrile IR bands derived from FWHM assuming Gaussian bandshapes.[1]

| PYP Construct | s.d., in $cm^{-1}$ | s.d., MV/cm[a,b] |
|---|---|---|
| F28oCNF | 5.4 | 28.4 |
| F62oCNF | 2.8 | 14.5 |
| F92oCNF | 6.1 | 32.2 |
| F96oCNF | 3.6 | 18.8 |

[a] Converted from $cm^{-1}$ to MV/cm using the POL MD Stark tuning rate[1] of 0.19 $cm^{-1}$/(MV/cm)

[b] Comparing the s.d.'s with those for nitrile electric field distributions from FC and POL MD (**Table S2**) indicates POL MD s.d.'s are closer to the experimentally-derived values for every PYP variant.

*Defining Nitrile H-Bond Geometric Cutoffs*

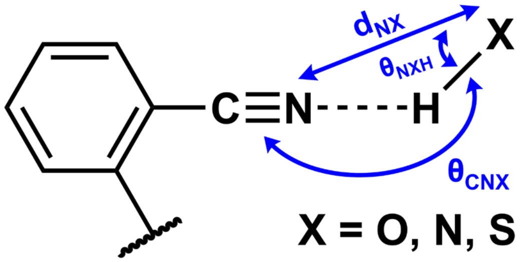


**Figure S2.** Geometric definitions for oCNF's nitrile's H-bonds. X refers to a heavy-atom H-bond donor. The dashed line indicates a potential H-bonding interaction. $\theta_{CNX}$ is the CNX angle, $\theta_{NXH}$ is the NXH angle, and $d_{NX}$ is the distance from the nitrile nitrogen (N) to the heavy-atom H-bond donor (X). In the text, $\theta_{CNX}$ is referred to as the "H-bond angle" and $d_{NX}$ is referred to as the "H-bond distance." This figure is similar to that in main text **Figure 1G** (which does not contain the $\theta_{NXH}$ definition).

To decide whether a given MD frame was H-bonding or not with respect to the oCNF nitrile, several geometric cutoffs needed to be established (with definitions for the relevant distances/angles provided in **Figure S2**). H-bond distances and angles between the heavy atoms (*i.e.*, distance or angle from the nitrile nitrogen to the donor heavy atom) are used throughout. We chose a cutoff for $\theta_{NXH}$ of 30° based on angles observed for nitriles engaged in C≡N···H–O interactions in small molecule crystallography (see Figure 4b in ref. [22]). To choose cutoffs for $d_{NX}$ (H-bond distance) and $\theta_{CNX}$ (H-bond angle), we used statistics from the 100 ns POL MD aggregate trajectories. To determine the cutoff distance, we calculated the simulated radial distribution function $g_{NX}(r)$ between the nitrogen atom of the nitrile (N) and the heavy atom of an H-bond donor (X) for each variant subject to the constraint $\theta_{NXH} \leq 30°$:

$$g_{NX}(r) = \frac{n}{N_F\left(\frac{4}{3}\pi(r+dr)^3 - \frac{4}{3}\pi r^3\right)}$$

where $r$ is $d_{NX}$, $dr$ is a (small) change in $d_{NX}$, $n$ is the number of particles observed between $r$ and $r+dr$ over the entire trajectory, and $N_F$ is the number of MD frames sampled, which accounts for averaging over time.[23] Note that the only constraint for the radial distribution functions is the $\theta_{NXH}$ criterion, so nitrile interactions with protein residues and solvent waters both contribute to g($r$)'s. **Figure S3A** shows g($r$) for each variant when $d_{NX}$ was binned every 0.05 Å (i.e., $dr$ = 0.05). At this stage, no cutoff was used for $\theta_{CNX}$. Maxima in g($r$) are observed at ∼ 3 Å for F28oCNF, F92oCNF, and F96oCNF, while no such maxima is observed for F62oCNF. Additionally, the profiles of the first interaction shell (*i.e.*, the "first solvation shell" in the protein setting) for F28oCNF, F92oCNF, and F96oCNF approximately decay to zero between 4 and 5 Å. As such, **Figure S3B** shows g($r$)'s for F28oCNF, F92oCNF, and F96oCNF as histograms with 0.05 Å bins in order to choose the H-bond cutoff distance with a finer perspective. We find 4.0 Å to be a good cutoff for the H-bond distance ($d_{NX}$) that maximizes the inclusion of the first interaction shell and minimizes the inclusion of the second shell.

To determine the cutoff for the H-bond angle ($\theta_{CNX}$), we examined the angle from the nitrile nitrogen to any potential H-bond donor's heavy atom that was between 60° and 180° and that was also less than or equal to 4.0 Å (the $d_{NX}$ criterion) and met our angle criterion for $\theta_{NXH}$; the angles were binned every 5° to generate histograms for F28oCNF (**Figure S20B**), F92oCNF (**Figure S22B**), and F96oCNF (**Figure S23B**), which are the left histogram in the figures. Previous definitions for the H-bond angle cutoff were influenced by histogramming the

angles observed in a data set of small organic molecules and identifying the shallowest angle for which interactions were observed (see Figure 3a in ref. [24]). In F28oCNF, potential H-bond angles were observed for interactions as shallow as ~ 65° (**Figure S20B**), so we chose not to set a cutoff for $\theta_{CNX}$. By combining the H-bond angle histograms with the histograms for the H-bond distance, we generated contour plots to visualize the nitrile H-bond angle/distance sampling space (B panels in **Figure S20**, **Figure S22,** and **Figure S23**; generated in OriginPro 2021b).

H-bond distance/angle histograms and contour plots were also created for all variants with FC MD (**Figure S21** and A panels in **Figure S20**, **Figure S22,** and **Figure S23**). The FC MD H-bond distance/angle histograms for F28oCNF and F92oCNF (*i.e.*, the variants with > 5% HB populations in FC MD; main text **Table 2**) significantly decay by 4.0 Å and have angles as shallow as 60°, indicating the cutoffs chosen from POL MD apply equally well.

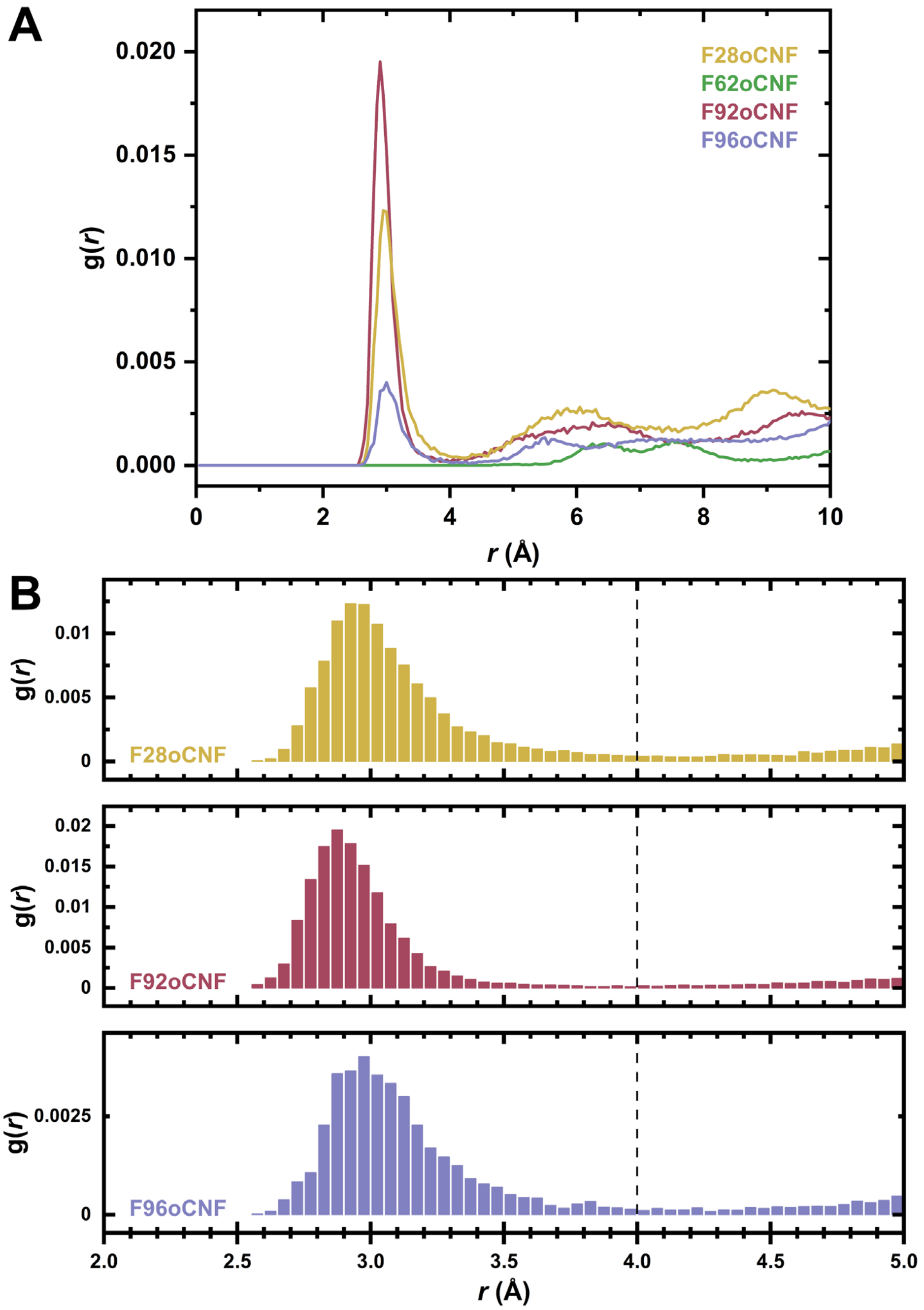


**Figure S3.** Radial distribution functions $g_{NX}(r)$ for the nitrile nitrogen (N) and the H-bond donor heavy atom (X). Only interactions satisfying the constraint $\theta_{NXH} \leq 30°$ were considered. In (A) and (B), nitriles in F28oCNF, F92oCNF, and F96oCNF show a clear peak in $g(r)$ around 2.9 – 3.1 Å followed by a decrease in intensity at larger distances. Histograms in (B) make clear that the first interaction shell ends for these nitrile groups at ~ 4.0 Å, marked by a vertical dashed line. As such, 4.0 Å was chosen for our H-bond distance cutoff. In contrast, $g(r)$ for F62oCNF does not show features in (A) until $r$ is 5 – 6 Å. Profiles for F28oCNF, F92oCNF, and F96oCNF in (A) are similar to those for the solvent exposed cinnamonitrile arm of the drug rilpivirine bound to the HIV-1 reverse transcriptase in another computational study;[25] the profile of F62oCNF is more analogous to that for the buried benzonitrile arm of rilpivirine complexed with mutants of HIV-1 reverse transcriptase (see Figure 4 in ref. [25]). Relative $g$(r) values at distances larger than 4 Å in this study are smaller than those observed in ref. [25] due to our constraint $\theta_{NXH} \leq 30°$.

*H-Bonding/Non-H-Bonding Populations*

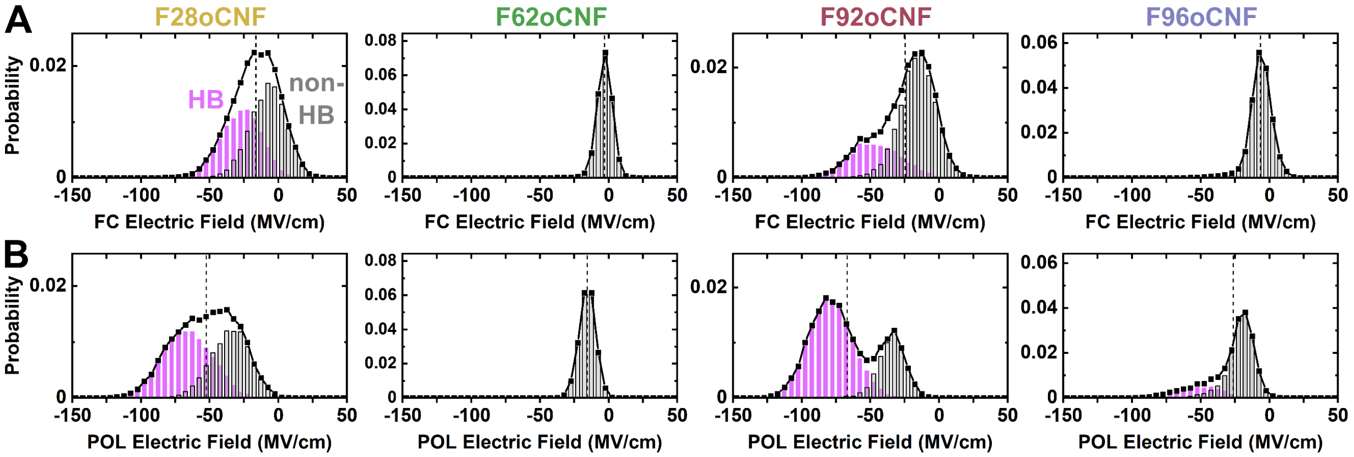


**Figure S4.** Calculated nitrile electric field H-bonding/non-H-bonding distributions subject to a 3.5 Å H-bond cutoff distance for (A) FC MD and (B) POL MD. Choice of H-bond distance cutoff negligibly alters the nitrile electric field distributions (compare with main text **Figure 2**) nor any properties derived from them (**Table S4**)**.** Each species with a population > 5% (**Table S4**) has an approximately symmetric distribution here or in main text **Figure 2** except F96oCNF's non-H-bonding population with POL MD, which is consistently skewed towards more negative electric fields; this behavior was previously observed for acetophenone's carbonyl in water with the generalized AMBER force field.[16] This may be because electric fields that are not sampled have a positive sign, meaning the electric field interaction with the nitrile would be *destabilizing*.

**Table S4.** Calculated nitrile (non-)H-bonding fractions, electric fields, and fraction-weighted electric fields using a 3.5 Å H-bond cutoff distance. Means and standard deviations were obtained by analyzing four individual FC MD runs and two individual POL MD runs. In contrast to the increasing H-bond fractions with POL MD displayed here and in main text **Table 2**, a recent report found MD with AMBER produced approximately 15 times more H-bonding for a protein-incorporated nitrile than AMOEBA MD,[26] indicating H-boding (when present) does not always increase with AMOEBA MD.

| Environment/ Force Field | H-bonding fraction (%) | non-H-bonding fraction (%)[a] | H-bonding median $F_{C≡N,MD}$ (MV/cm) | non-H-bonding median $F_{C≡N,MD}$ (MV/cm) | Fraction-weighted $F_{C≡N,MD}$ (MV/cm) |
|---|---|---|---|---|---|
| F28oCNF/FC | 45.8 ± 1.1 | 54.2 | -26.6[b] | -7.2 ± 0.2 | -16.1 ± 0.3 |
| F28oCNF/POL | 55.7 ± 5.4 | 44.3 | -66.6 ± 1.8 | -34.6 ± 1.8 | -52.4 ± 0.4 |
| F62oCNF/FC | 1.4 ± 1.4 | 98.6 | -18.5[c] | -2.7 ± 0.4 | -3.0 ± 0.2 |
| F62oCNF/POL | 0 | 100 | N/A | -15.4 ± 1.0 | -15.4 ± 1.0 |
| F92oCNF/FC | 29.3 ± 3.7 | 70.7 | -46.1 ± 0.7 | -15.5 ± 1.1 | -24.5 ± 0.1 |
| F92oCNF/POL | 72.3 ± 11.0 | 27.7 | -79.2 ± 1.8 | -33.9 ± 1.0 | -66.8 ± 5.0 |
| F96oCNF/FC | 2.9 ± 2.1 | 97.1 | -30.1 ± 0.5 | -5.8 ± 0.2 | -6.5 ± 0.3 |
| F96oCNF/POL | 20.3 ± 6.9 | 79.7 | -52.1 ± 3.2 | -19.7 ± 0.3 | -26.2 ± 1.8 |

[a] Same error as for H-bonding fraction

[b] Error <0.1 MV/cm

[c] No error could be determined because only one trajectory had H-bonding

**Table S5.** Standard deviations (s.d.'s) of the electric field distributions of calculated nitrile H-bonding/non-H-bonding populations (with a 4.0 Å H-bond cutoff distance) for fixed charge (FC) and polarizable (POL) MD simulations.

| PYP Construct | s.d. H-bonding $F_{C\equiv N,FC\ MD}$ (MV/cm) | s.d. non-H-bonding $F_{C\equiv N,FC\ MD}$ (MV/cm) | s.d. H-bonding $F_{C\equiv N,POL\ MD}$ (MV/cm) | s.d. non-H-bonding $F_{C\equiv N,POL\ MD}$ (MV/cm) |
|---|---|---|---|---|
| F28oCNF | 15.5 | 13.1 | 19.1 | 14.5 |
| F62oCNF | 9.6 | 5.2 | N/A | 5.8 |
| F92oCNF | 19.6 | 12.9 | 16.9 | 10.6 |
| F96oCNF | 15.2 | 7.2 | 17.2 | 10.0 |

## S4 Assessment of Time-Dependent MD Quantities

*Time-Dependence of Electric Fields and H-Bonding*

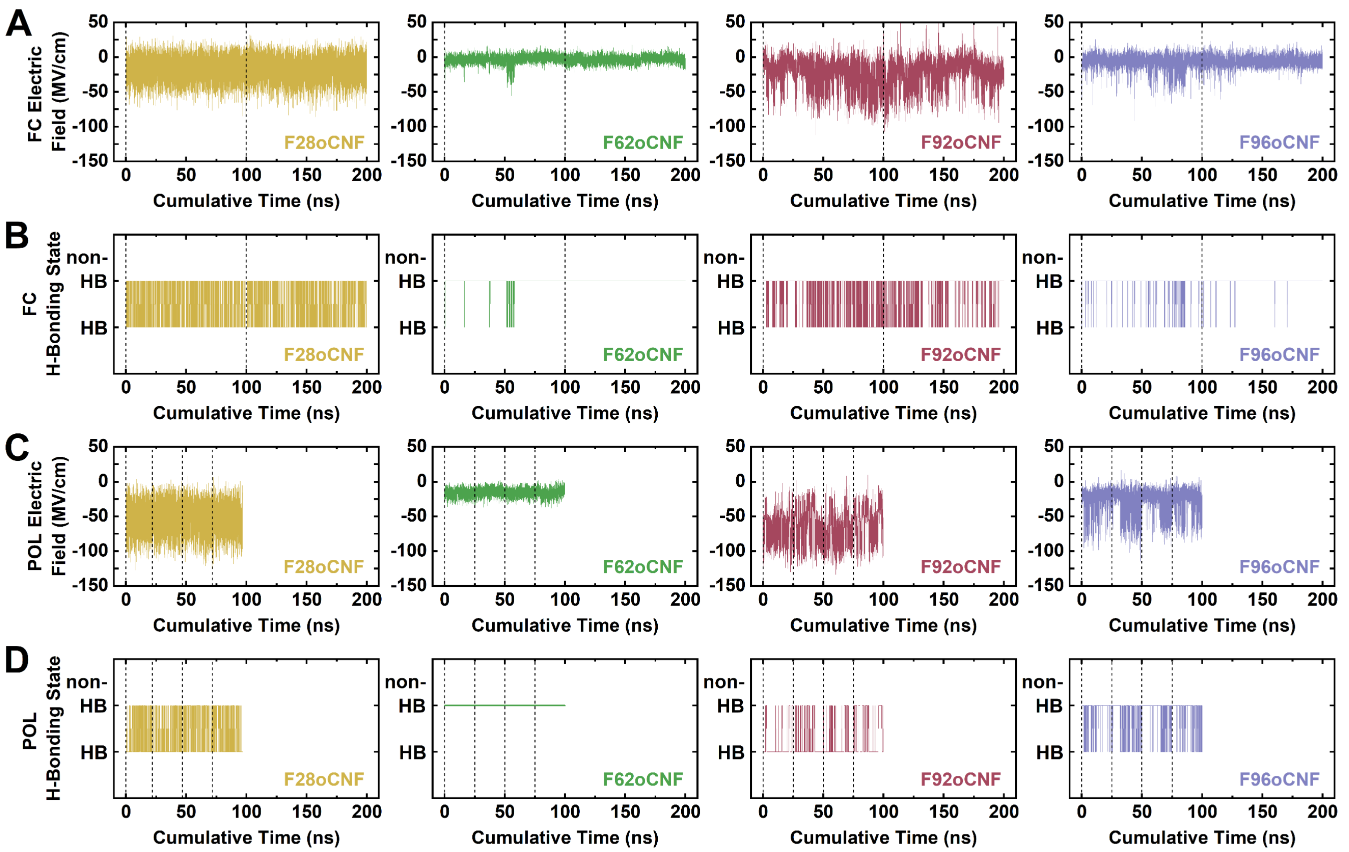


**Figure S5.** Simulated time-dependence of nitrile electric field and H-bonding state for PYP variants. (A) and (B) are derived from FC MD and (C) and (D) are derived from POL MD. In (B) and (D), one in every ten states is displayed for the sake of visibility. Dashed lines indicate the start of an independent replicate. Frequencies of shorter timescale H-bonding/non-H-bonding residences (< 250 ps) are displayed in **Figure S6** (FC MD) and **Figure S7** (POL MD) and frequencies of longer timescale residences (250+ ps) are displayed in **Figure S8**. Note that POL MD for F28oCNF was run for slightly less than 100 ns (97 ns).

*Non-H-Bonding/H-Bonding State Residence Times*

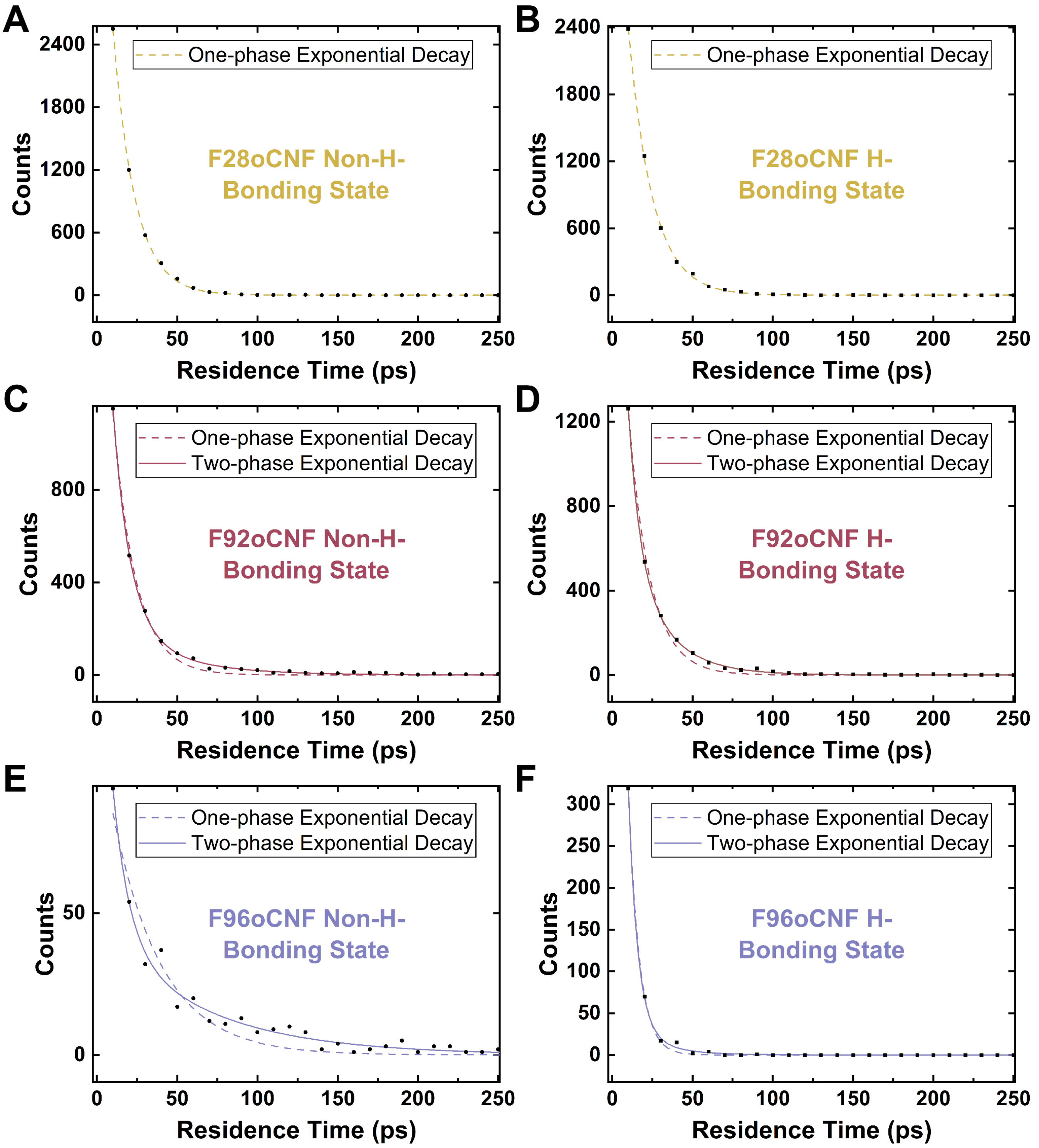


**Figure S6.** Frequency of residence times in FC MD of the non-H-bonding (circles) and H-bonding states (squares) for (A/B) F28oCNF, (C/D), F92oCNF, and (E/F) F96oCNF. Fits were performed with either one or two decaying exponential functions (**Table S6**).

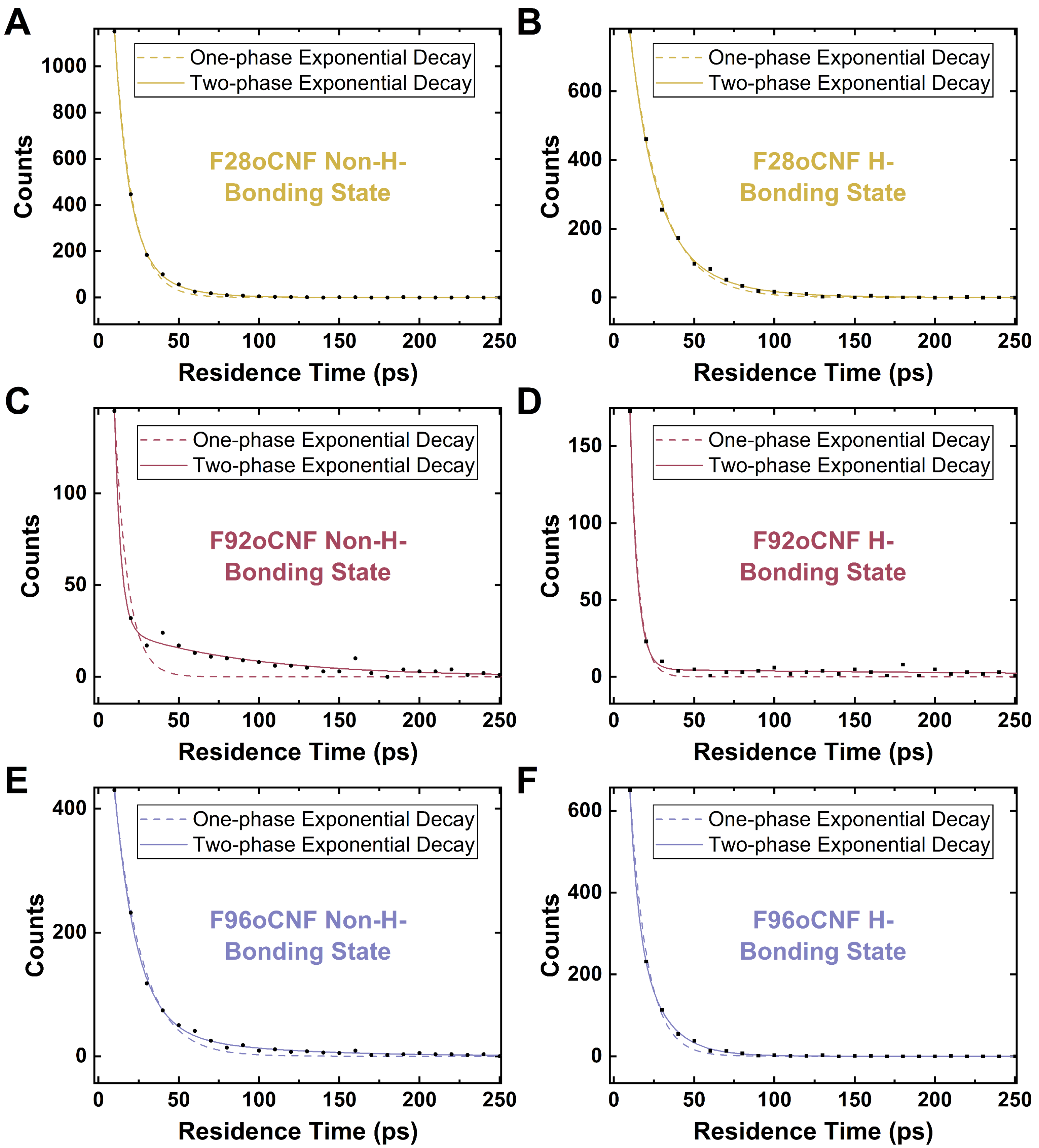


**Figure S7.** Frequency of residence times in POL MD of the non-H-bonding (circles) and H-bonding states (squares) for (A/B) F28oCNF, (C/D), F92oCNF, and (E/F) F96oCNF. Fits were performed with either one or two decaying exponential functions (**Table S7)**.

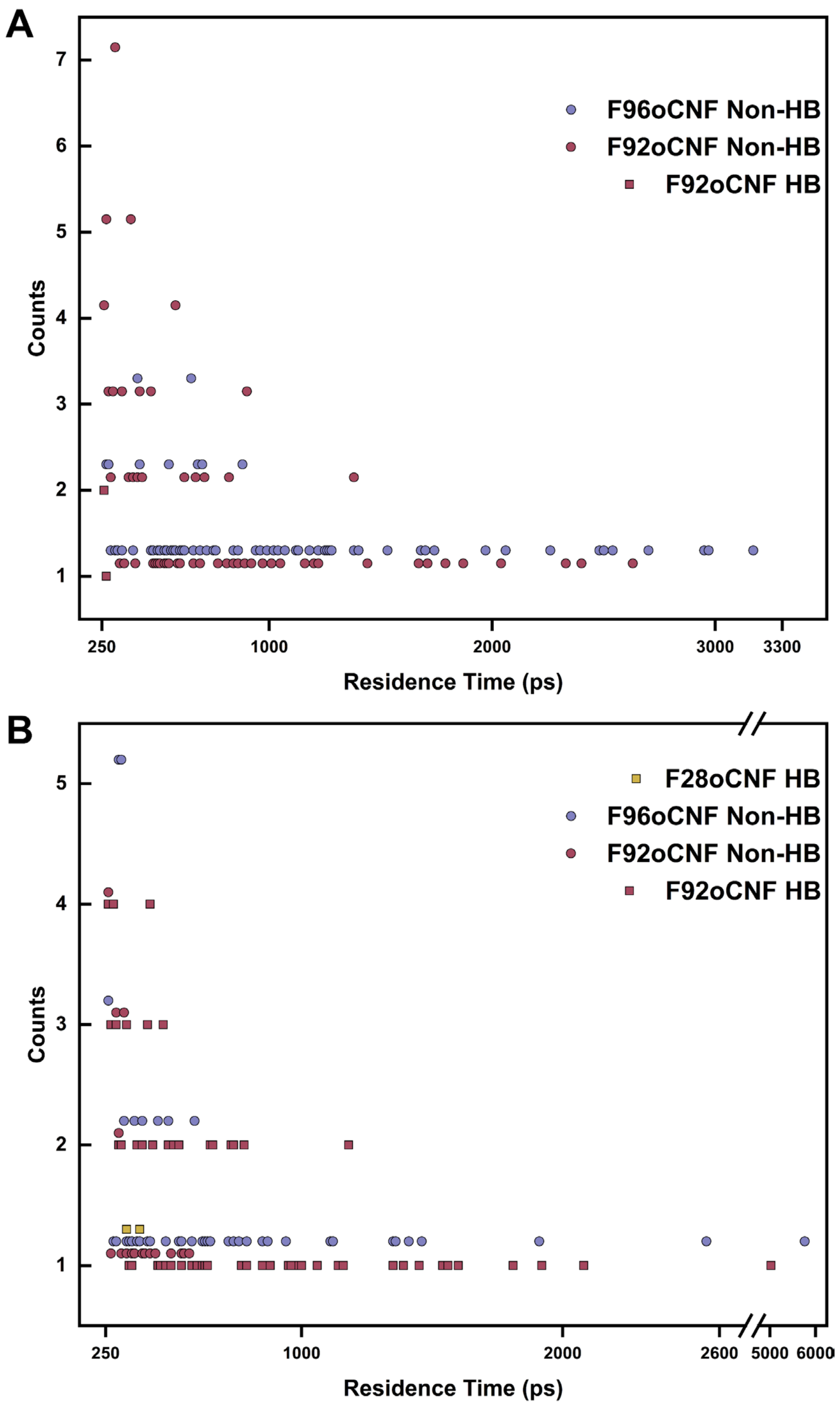


**Figure S8.** Frequency of residence times longer than 250 ps in (A) FC and (B) POL MD for the nitrile non-H-bonding (circles) and H-bonding states (squares). Different variant and H-bonding/non-H-bonding combinations are offset such that any value with counts between *x* and *x+1* had *x* counts observed. In (A), F92oCNF's non-H-bonding state and F96oCNF's H-bonding state demonstrate many long residence times. In (B), F92oCNF's H-bonding state and F96oCNF's H-bonding state demonstrate many long residence times. The biggest difference between FC and POL MD occurs for F92oCNF, where the predominance of long residence times switches from the non-H-bonding state to the H-bonding state. Too few counts were observed for any species to extract lifetimes.

*Fitting Non-H-Bonding/H-Bonding State Lifetimes*

Single and double decaying exponentials fits to the plots in **Figure S6** and **Figure S7** have the form:

$$c = A_1 exp\left(\frac{-t_r}{t_1}\right)$$

or

$$c = A_1 exp\left(\frac{-t_r}{\tau_1}\right) + A_2 exp\left(\frac{-t_r}{\tau_2}\right)$$

where $c$ is the number of counts, $t_r$ is the residence time, and $A_1/A_2$ and $\tau_1/\tau_2$ are the amplitudes and lifetimes, respectively, of the first and second exponentials. For fits with two exponentials, the amplitude-weighted average lifetime ($\tau_A$) is[27]

$$\tau_A = \frac{A_1\tau_1 + A_2\tau_2}{A_1 + A_2}$$

The fitting coefficients for **Figure S6** (FC MD) and **Figure S7** (POL MD) are provided in **Table S6** and **Table S7**, respectively. Note that MD frames were output every 10 ps and several lifetimes from fitting are < 10 ps. A more precise determination of these values would require frames to be output more frequently than every 10 ps. Despite this, we treat these values quantitatively in the following analysis.

**Table S6.** Parameters from exponential fits for non-H-bonding and H-bonding nitrile populations from FC MD.

| PYP Construct | State | $A_1$ | $\tau_1$ (ps) | $A_2$ | $\tau_2$ (ps) | $\tau_A$ (ps) |
|---|---|---|---|---|---|---|
| F28oCNF | Non-H-Bonding | 5307 ± 19 | 13.6 ± 0.1 | | | |
| F28oCNF | H-Bonding | 4676 ± 20 | 14.9 ± 0.1 | | | |
| F92oCNF | Non-H-Bonding | 2305 ± 34 | 14.1 ± 0.2 | | | |
| F92oCNF | Non-H-Bonding | 2421 ± 29 | 10.5 ± 0.3 | 275 ± 41 | 38 ± 3 | 13.3 ± 0.6 |
| F92oCNF | H-Bonding | 2613 ± 41 | 13.5 ± 0.2 | | | |
| F92oCNF | H-Bonding | 2850 ± 51 | 7.0 ± 0.3 | 889 ± 53 | 23.0 ± 0.6 | 10.8 ± 0.3 |
| F96oCNF | Non-H-Bonding | 147 ± 14 | 10 ± 1 | 456 ± 6 | 64 ± 6 | 51 ± 5 |
| F96oCNF | H-Bonding | 1408 ± 24 | 6.7 ± 0.1 | | | |
| F96oCNF | H-Bonding | 1671 ± 58 | 5.7 ± 0.2 | 56 ± 22 | 20 ± 3 | 6.2 ± 0.3 |

**Table S7.** Parameters from exponential fits for non-H-bonding and H-bonding nitrile populations from POL MD.

| PYP Construct | State | $A_1$ | $\tau_1$ (ps) | $A_2$ | $\tau_2$ (ps) | $\tau_A$ (ps) |
|---|---|---|---|---|---|---|
| F28oCNF | Non-H-Bonding | 2828 ± 27 | 11.1 ± 0.1 | | | |
| F28oCNF | Non-H-Bonding | 2868 ± 32 | 8.6 ± 0.2 | 400 ± 54 | 22 ± 1 | 10.2 ± 0.3 |
| F28oCNF | H-Bonding | 1270 ± 11 | 19.8 ± 0.2 | | | |
| F28oCNF | H-Bonding | 1068 ± 88 | 15 ± 1 | 309 ± 101 | 34 ± 4 | 19 ± 2 |
| F92oCNF | Non-H-Bonding | 1636 ± 304 | 3.8 ± 0.3 | 29 ± 2 | 81 ± 5 | 5.1 ± 0.4 |
| F92oCNF | H-Bonding | 1490 ± 101 | 4.6 ± 0.3 | 5.0 ± 0.5 | 343 ± 46 | 5.7 ± 0.4 |
| F96oCNF | Non-H-Bonding | 758 ± 12 | 17.3 ± 0.3 | | | |
| F96oCNF | Non-H-Bonding | 790 ± 8 | 13.8 ± 0.3 | 55 ± 8 | 68 ± 6 | 17.3 ± 0.7 |
| F96oCNF | H-Bonding | 1625 ± 26 | 10.8 ± 0.1 | | | |
| F96oCNF | H-Bonding | 2262 ± 134 | 5.0 ± 0.3 | 607 ± 38 | 17 ± 1 | 7.5 ± 0.4 |

*Two-State First Order Exchange Model of Nitrile H-Bonding*

Nitrile populations experiencing H-bonding (as assessed by the TDM VSE analysis)[1] had two populations in the electric field distributions from MD (main text **Figure 2**). The appearance of two populations contrasts with the room temperature IR spectra, where all spectra were well-fit with a single, symmetric band[1] (**Figure S11**; similarly, a recent paper performed MD of benzonitrile in water in order to calculate vibrational spectra and predicted two IR bands, while only one is experimentally observed).[28] To address this inconsistency, we compared the calculated lifetimes of the nitrile's H-bonding/non-H-bonding states with the experimental nitrile dephasing lifetime, since peak averaging can occur if the state lifetimes are faster than or the same order of magnitude as the probe's lifetime.[29] H-bonding and non-H-bonding lifetimes were predominately in the range of 5 – 20 ps (**Tables S6-S7**), while aromatic nitrile vibrational lifetimes are typically on the order of several ps.[30] While we were unable to find a reported vibrational lifetime for oCNF, the lifetime for the nitrile of *p*-cyanophenylalnine (pCNF) has been previously measured as 4.0 ± 0.2 ps.[31] Additionally, pCNF incorporated into a peptide has a vibrational lifetime of 4.3 ± 0.2 ps whether the peptide is free in solution or complexed with a protein where the nitrile is inserted into the protein interior.[32] We therefore assume oCNF's nitrile has a vibrational lifetime of ~ 4 ps. This value is approximately the same order of magnitude as the lifetimes observed in **Tables S6-S7**, raising the possibility that nitrile H-bonding and non-H-bonding populations are averaged in the room temperature linear IR experiments.

To further test this possibility, we compared the calculated chemical exchange rates from MD with predicted energy differences between the H-bonding/non-H-bonding states (defined as $|\Delta\bar{\nu}|$) to determine which chemical exchange regime (a concept well known in the NMR[33,34] and 2D IR[29] communities) the nitrile H-bonding is in.[34] For the following analysis, we consider a simple two state model of H-bonded and non-H-bonded nitriles:

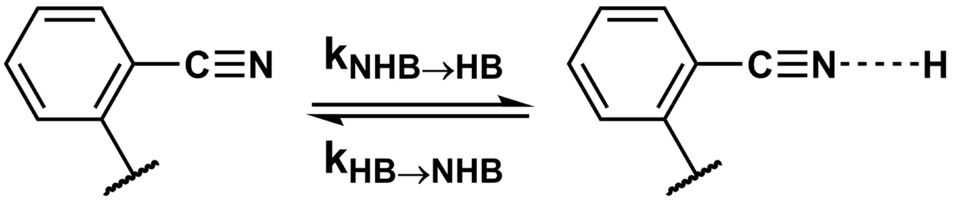


**Scheme S1**. Two-state first order exchange model for nitrile H-bonding.

Lifetimes of H-bonding and non-H-bonding states in **Tables S6-S7** were converted to rate constants, which were summed to obtain an exchange rate ($k_{ex}$; **Table S8**).[34] To obtain the predicted difference in energy between the H-bonding and non-H-bonding states ($|\Delta\bar{\nu}|$), we recall that the nitrile frequency is described by

$$\bar{\nu}_{\mathrm{obs}} = \bar{\nu}_0 - \Delta\vec{\mu} \cdot \vec{F} + \Delta\bar{\nu}_{\mathrm{HB}}$$

where $\bar{\nu}_{\mathrm{obs}}$ is the observed frequency, $\bar{\nu}_0$ is the gas-phase frequency, $\Delta\vec{\mu}$ is the vibrational Stark tuning rate, $\vec{F}$ is the electric field, and $\Delta\bar{\nu}_{\mathrm{HB}}$ is the H-bond contribution to the frequency not captured by the VSE, *i.e.*, the H-bonding blueshift.[1] We first used the FC MD or POL MD solvatochromic oTN Stark tuning rates[1] with the median H-bonding/non-H-bonding electric fields from main text **Table 2** to predict the contribution to the nitrile frequency tuning by the VSE; the predicted differences between the H-bonding and non-H-bonding nitrile frequencies range from -4 to -9 $cm^{-1}$ (**Table S9**). Note that the values in **Table S9** are obtained using the assumption that H-bonded and non-H-bonded nitriles have the same intrinsic Stark tuning rate; this was verified by vibrational Stark spectroscopy, described in the subsection titled "Assessment of the H-Bonded vs Non-H-Bonded Nitrile Vibrational Stark Tuning Rates."

The predicted frequencies for the non-H-bonding populations in **Table S9** account for all relevant frequency tuning mechanisms, but the frequencies for the H-bonding populations still require an estimate of the $\Delta\bar{\nu}_{\mathrm{HB}}$ values. Unfortunately, a quantitative prediction of this term is challenging. Prior estimates of $\Delta\bar{\nu}_{\mathrm{HB}}$ suggest it can be as large as $\sim$ 20 cm$^{-1}$.[1,35] Using the purely VSE-based predicted nitrile frequency differences in **Table S9** and considering $\Delta\bar{\nu}_{\mathrm{HB}}$'s of the extrema (0 and 20 cm$^{-1}$), we may then expect $|\Delta\bar{\nu}|$ to be in the range of -10 to +15 cm$^{-1}$. In prior work, a room temperature nitrile frequency difference between H-bonding and non-H-bonding nitrile populations was 13 cm$^{-1}$,[36] while the difference in F92oCNF's populations in 50:50 water:glycerol at 303 K is 14 cm$^{-1}$ (**Table S12**). Both observed values for $|\Delta\bar{\nu}|$ suggest our computational estimate on the upper, positive end is reasonable.

If we convert the exchange rates from **Table S8** to units of cm$^{-1}$ (**Table S10**), we find $|\Delta\bar{\nu}|$ and $k_{\mathrm{ex}}$ derived from the MD simulations are predicted to be of the same order of magnitude. This is defined as the intermediate exchange regime (in contrast to fast or slow exchange, where $k_{\mathrm{ex}}$ is significantly larger or smaller than $|\Delta\bar{\nu}|$, respectively).[34] In the intermediate exchange regime, one signal is observed as the individual populations begin to coalesce.[34] This exchange regime was previously observed for the temperature-dependent IR of pCNF in water, wherein the nitrile peaks broadened (but did not fully resolve) when going from 87 to 3°C (*i.e.,* transition from fast to intermediate regime),[37] and in studies of acetonitrile in methanol, where varying the temperature from 50 to -17°C resolved one broad peak into two distinct peaks (*i.e.,* transition from intermediate to slow regime).[29,38] These previous results suggest that nitrile H-bonding can indeed exist near the intermediate chemical exchange regime. Therefore, this analysis suggests the nitriles with experimental H-bonding shifts have H-bonding/non-H-bonding kinetics in the intermediate exchange regime, which can explain why the MD simulations and the IR experiments give rise to two populations or one population, respectively. (On a final note, if $\Delta\bar{\nu}_{\mathrm{HB}}$ were in the range of 5 – 10 cm$^{-1}$, then $|\Delta\bar{\nu}|$ may be close to zero (**Table S9**). In this case, the frequencies of the underlying populations may be too close to be spectrally resolved at room temperature. Recent calibrations suggest these values of $\Delta\bar{\nu}_{\mathrm{HB}}$ are attainable,[1,35] meaning there are (at least) two viable explanations for the apparent inconsistency in the number of IR and MD populations.)

**Table S8.** Individual rate constants (derived from **Tables S6-S7**) and exchange rates for non-H-bonding and H-bonding nitriles. In cases where lifetimes were fit with two exponential decays (**Figures S6-S7**), the amplitude-weighted lifetimes were used to derive rates. Exchange rates are the sum of the individual rate constants.

| Environment/ Force Field | Non-H-Bonding to H-Bonding Rate Constant ($k_{NHB \rightarrow HB}$; $ps^{-1}$) | H-Bonding to Non-H-Bonding Rate Constant ($k_{HB \rightarrow NHB}$; $ps^{-1}$) | Exchange Rate ($k_{ex}$; $ps^{-1}$) |
|---|---|---|---|
| F28oCNF/FC | 0.0735 ± 0.0005 | 0.0671 ± 0.0005 | 0.1406 ± 0.0007 |
| F28oCNF/POL | 0.098 ± 0.003 | 0.053 ± 0.006 | 0.151 ± 0.007 |
| F92oCNF/FC | 0.075 ± 0.003 | 0.093 ± 0.003 | 0.168 ± 0.004 |
| F92oCNF/POL | 0.20 ± 0.02 | 0.18 ± 0.01 | 0.38 ± 0.02 |
| F96oCNF/FC | 0.020 ± 0.002 | 0.161 ± 0.008 | 0.181 ± 0.008 |
| F96oCNF/POL | 0.058 ± 0.002 | 0.133 ± 0.007 | 0.191 ± 0.007 |

**Table S9.** Predicted nitrile frequencies if the VSE were the only mechanism of frequency tuning.

| Environment/ Force Field | H-bonding ($cm^{-1}$) | Non-H-Bonding ($cm^{-1}$) | H-bonding – Non-H-Bonding Difference ($cm^{-1}$) |
|---|---|---|---|
| F28oCNF/FC | 2223.3 ± 0.1 | 2227.9 ± 0.1 | -4.6 ± 0.1 |
| F28oCNF/POL | 2219.1 ± 0.3 | 2224.9 ± 0.3 | -5.8 ± 0.4 |
| F92oCNF/FC | 2218.1 ± 0.1 | 2225.7 ± 0.2 | -7.6 ± 0.2 |
| F92oCNF/POL | 2216.5 ± 0.4 | 2225.0 ± 0.2 | -8.5 ± 0.4 |
| F96oCNF/FC | 2223.1 ± 0.2 | 2228.2 ± 0.4 | -5.1 ± 0.4 |
| F96oCNF/POL | 2222.1 ± 0.3 | 2227.7 ± 0.1 | -5.6 ± 0.3 |

**Table S10.** Energy associated with nitrile exchange rates in **Table S8**.

| Environment/ Force Field | $k_{ex}$ in $cm^{-1}$ |
|---|---|
| F28oCNF/FC | 4.69 ± 0.02 |
| F28oCNF/POL | 5.0 ± 0.2 |
| F92oCNF/FC | 5.6 ± 0.1 |
| F92oCNF/POL | 12.7 ± 0.7 |
| F96oCNF/FC | 6.0 ± 0.3 |
| F96oCNF/POL | 6.4 ± 0.2 |

*Predicting H-Bonding Fractions from the Exchange Model*

The exchange model in **Scheme S1** allows for prediction of an H-bonding fraction by taking the quotient of the rate constant for the conversion of the non-H-bonding to H-bonding state ($k_{NHB \rightarrow HB}$) and the corresponding chemical exchange rate:[34] these was obtained for F28oCNF, F92oCNF, and F96oCNF with FC and POL MD, as were predictions of the non-H-bonding fractions by consideration of the H-bonding to non-H-bonding rate constants ($k_{HB \rightarrow NHB}$; **Table S11**). All predicted H-bonding fractions are larger for POL MD than FC MD, matching the results obtained by assigning each MD frame as H-bonding or non-H-bonding using H-bonding cutoffs (**Table 2**). More quantitatively, the predicted values from the exchange model compare quite well with fractions in the main text for F28oCNF with both FFs (within 50.4 – 52.6% for FC MD and 54.4 – 65.2% for POL MD, the 1σ confidence intervals obtained by assigning frames) and do reasonably well for F92oCNF POL MD and F96oCNF, as the predicted H-bonding fractions are all (effectively) within the main text 2σ confidence intervals (52.8 – 95.2% and 0 – 8.5%/7.6 – 37.6% for F92oCNF POL MD and F96oNCF with FC MD/POL MD, respectively). F92oCNF and F96oCNF simulated with FC MD require going beyond the 2σ CIs before the predicted values from the exchange model are within the intervals, though this may not be surprising for F96oCNF since the F96oCNF FC MD had the fewest observed H-bonding events for a nitrile with an experimental H-bonding shift. The origin of the disagreement for the H-bonding fraction between the two methods for F92oCNF FC MD is less clear, though we already know there are sampling issues for the FC MD given the MD-derived electric field differences observed in the main text. Nevertheless, the ability for the predicted H-bonding fractions from the exchange model to largely recapitulate the H-bonding fractions obtained by assigning frames, particularly for the values from POL MD, indicates our use of **Scheme S1** in the above analysis is sufficient to describe nitrile exchange reasonably well.

**Table S11.** Predicted nitrile H-bonding and non-H-bonding fraction from the quotient of the rate constants to the exchange rate (**Table S8**).

| Environment/ Force Field | H-bonding (%) | Non-H-Bonding (%) |
|---|---|---|
| F28oCNF/FC | 52.3 | 47.7 |
| F28oCNF/POL | 64.9 | 35.1 |
| F92oCNF/FC | 44.6 | 55.4 |
| F92oCNF/POL | 52.6 | 47.4 |
| F96oCNF/FC | 11.1 | 88.9 |
| F96oCNF/POL | 30.4 | 69.6 |

*Assessment of the H-Bonded vs Non-H-Bonded Nitrile Vibrational Stark Tuning Rates*

As a justification for the analysis presented in the subsection "Two-State First Order Exchange Model of Nitrile H-Bonding," we wished to address whether H-bonding alters the intrinsic sensitivity of nitrile groups to the electric field, called the linear Stark tuning rate. Because it is technically challenging to measure the vibrational Stark tuning rates of probes incorporated into a protein *in situ* using vibrational Stark spectroscopy (VSS),[17] a model system was employed. The measurements and much of the analysis in this subsection were performed and initially described by Drs. Nick Levinson and Sayan Bagchi. Note that a description of the methods used here can be found in ref. [1], Section S9.

Using benzonitrile as our model nitrile H-bond acceptor, we found that benzonitrile forms a H-bond with phenol in toluene, a glass forming solvent that is convenient for performing VSS. In mixtures of benzonitrile and phenol, a Fermi resonance confounds the precise assignment of the free and hydrogen bonded nitrile bands, so we used benzonitrile in which the nitrile group was isotopically labeled with $^{13}$C carbon, which redshifts the nitrile vibrational frequency by ~ 50 cm$^{-1}$, removing the overlap with the Fermi resonance. The experiments described below were performed with this isotopically labeled benzonitrile.

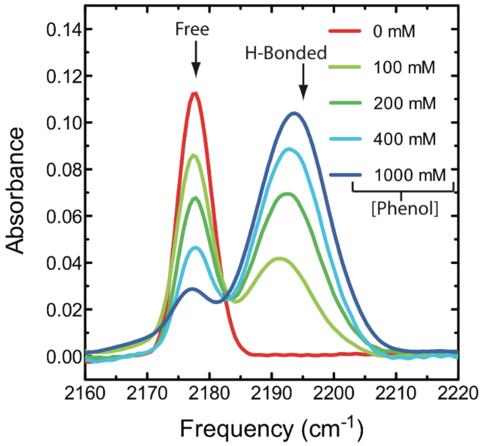


**Figure S9.** IR spectra of mixtures of 200 mM benzonitrile ($^{13}$C≡N) at different concentrations of phenol in toluene, measured at 77 K.

Titrating phenol into 200 mM benzonitrile in toluene results in the gradual appearance of a broad absorbance band 16 cm$^{-1}$ to the blue of the free benzonitrile frequency, consistent with the formation of a H-bond (**Figure S9**). To assess whether the formation of this H-bonding interaction alters the intrinsic sensitivity of the nitrile group to an electric field, we measured the vibrational Stark spectrum of a benzonitrile/phenol/toluene mixture. In this experiment, an external electric field is applied across the frozen sample and the effect of the field on the absorbance spectrum is measured. The linear Stark tuning rate ($f|\Delta\vec{\mu}|$, where $f$ is the local field factor)[1,18] of the probe can be determined by fitting the Stark spectrum (the difference between the field on and field off absorbance spectra) with a sum of the derivatives of the absorbance spectrum; the linear Stark tuning rate is proportional to the square root of the 2nd-derivative component of the fit.[1,18,39] In the case of overlapping bands arising from different chemical species, this fitting procedure will not suffice unless all underlying species have identical – or very similar – linear Stark tuning rates. In the case where one set of electrooptic parameters does not suffice, it would be necessary to model the absorbance spectra with basis functions representing the spectrum of each underlying feature and attempt to fit the Stark spectrum with numerical derivatives of these basis functions. Thus, the ability to fit the Stark spectrum of a mixture of species with the derivatives of the experimental

absorption spectrum constitutes a sensitive test of whether the Stark tuning rates of the underlying species are similar.

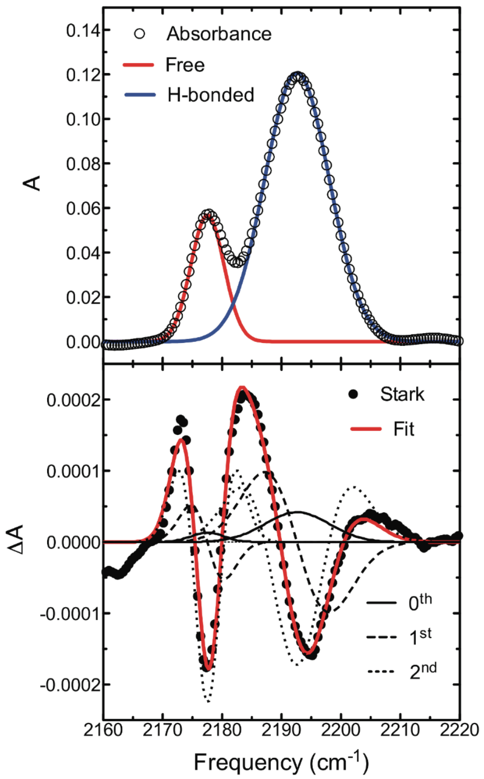


**Figure S10.** Absorbance (top) and Stark (bottom) spectra of 200 mM benzonitrile $^{13}$C in toluene with 400 mM phenol. Fitting was performed by modeling the absorbance as two Gaussian basis functions, corresponding to the free benzonitrile (red) and H-bonded benzonitrile (blue). The Stark spectrum was fit to the sum of the derivatives of these basis functions assuming a single set of electrooptic parameters. In the Stark spectrum, the 0$^{th}$, 1$^{st}$, and 2$^{nd}$ derivative components are shown as black solid, dashed, and dotted lines, respectively, and the overall fit is shown in red.

We chose a benzonitrile/phenol/toluene mixture in which the free and H-bonded peaks were roughly equally represented in the Stark spectrum, to maximize the discriminatory power of this test. We found that the simple numerical fitting procedure described above *using a single set of electrooptic parameters* produced a good fit to the Stark spectrum, adequately accounting for both prominent features arising from the free and H-bonded benzonitrile molecules (**Figure S10**). The quality of the fit shown in **Figure S10** provides experimental evidence that H-bonded and non-H-bonded nitriles have the same Stark tuning rate, justifying this assumption in our prior analysis.

## S5 Temperature-Dependent FTIR Spectra Characterization

*PYP Variants' Room Temperature Spectra in Buffer*

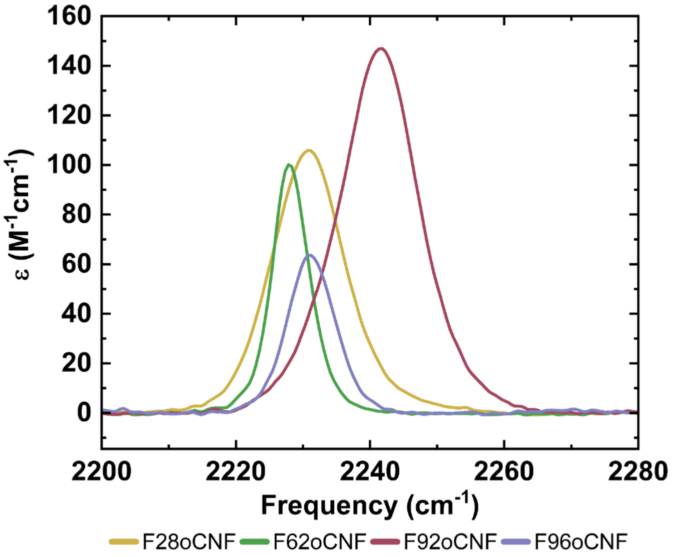


**Figure S11.** IR spectra at room temperature in buffer for F28oCNF, F62oCNF, F92oCNF, and F96oCNF PYP. Reproduced from ref. [1].

*100 K IR Spectra for F28oCNF and F96oCNF*

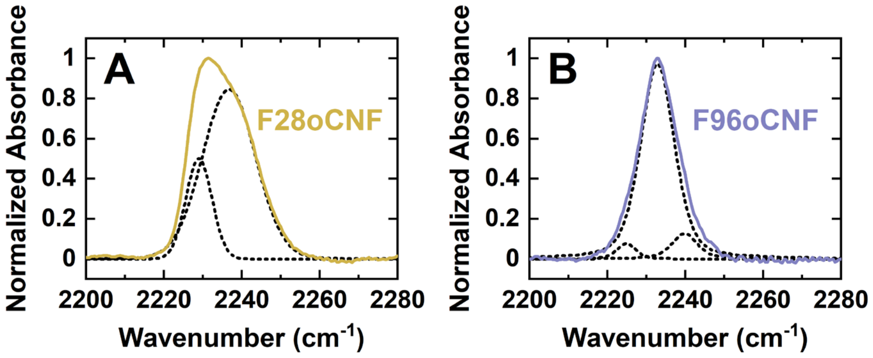


**Figure S12.** IR spectra at 100 K for (A) F28oCNF and (B) F96oCNF PYP. The solid lines are the experimental data, while dashed lines are the result of fitting the spectra. Band fitting for F28oCNF was reasonably straightforward, but fitting F96oCNF presented a challenge, described in the succeeding discussion.

The F96oCNF band at 100 K demonstrates a unique shape that made band fitting challenging. It has an especially broad base that is not typically observed in IR bands. When we attempted to fit it with a single pseudo-Voigt bandshape (**Figure S13A**), the resulting band had an 80 – 90% Lorentzian contribution, which is unusual for an IR band since they typically have larger Gaussian contributions due to inhomogeneous broadening. Additionally, when we simultaneously fit the absorption and the second derivative of the absorption in order to constrain the fit, the second derivative fit did not adequately reproduce the experimentally-derived second derivative (red curve in **Figure S13B**). **Figure S13B** makes clear that the second derivative derived from the absorption (in black) has features in the wings that suggest small populations at the high and low energy sides of the band. These second derivative features were not satisfactorily reproduced until three populations were included, as indicated in **Figure S13C,D**. On a final note, in **Figures S17-S18**, it can be seen that the F96oCNF spectra narrow from 233 K to 323 K (FWHM are provided in **Table S12**). This is the *opposite* of what is typically observed as temperature increases for a liquid solution (and is often observed when species pass from the intermediate to fast chemical exchange regimes), further supporting the notion that the 100 K spectrum contains multiple underlying populations.

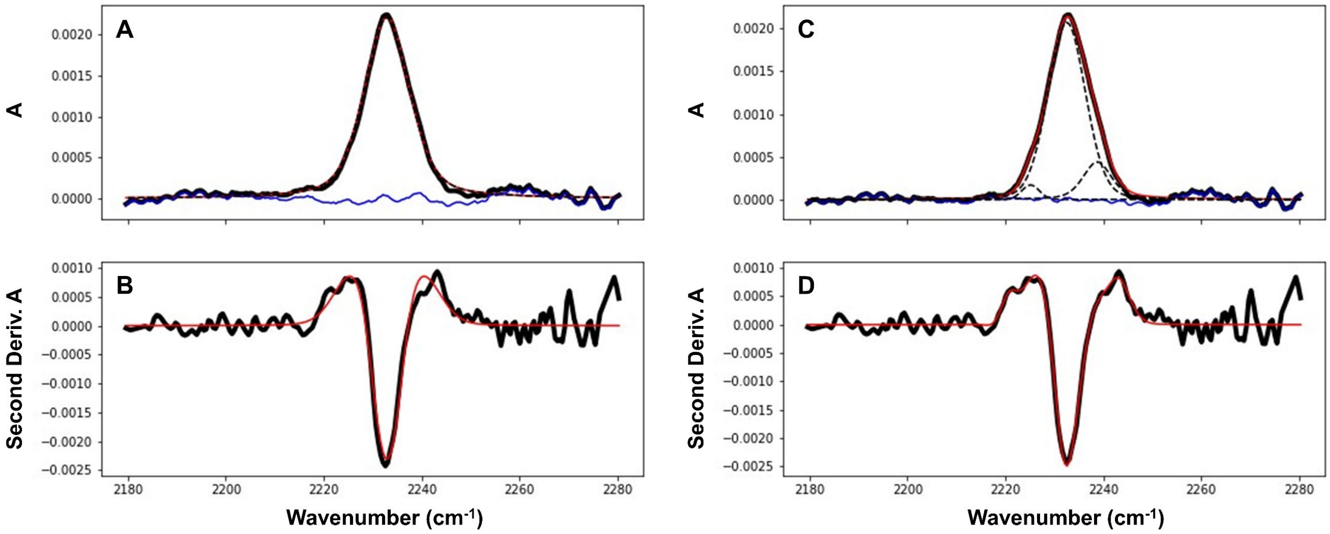


**Figure S13.** F96oCNF absorption spectra at 100 K can be successfully fit with one (A) or three bands (C), but the second derivatives (B/D) can only be recapitulated with three bands. Blue lines indicate the baseline, solid black lines indicate the experimental data, and black dashed lines/red lines indicate fits.

*IR Spectra Obtained Between 100 K and 323 K*

IR spectra were obtained not just at 100 K as shown in main text **Figure 3** and **Figure S12** but also at seven additional temperatures: 123 K, 160 K, 200 K, 233 K, 283 K, 303 K, and 323 K. Spectra at those temperatures are shown below.

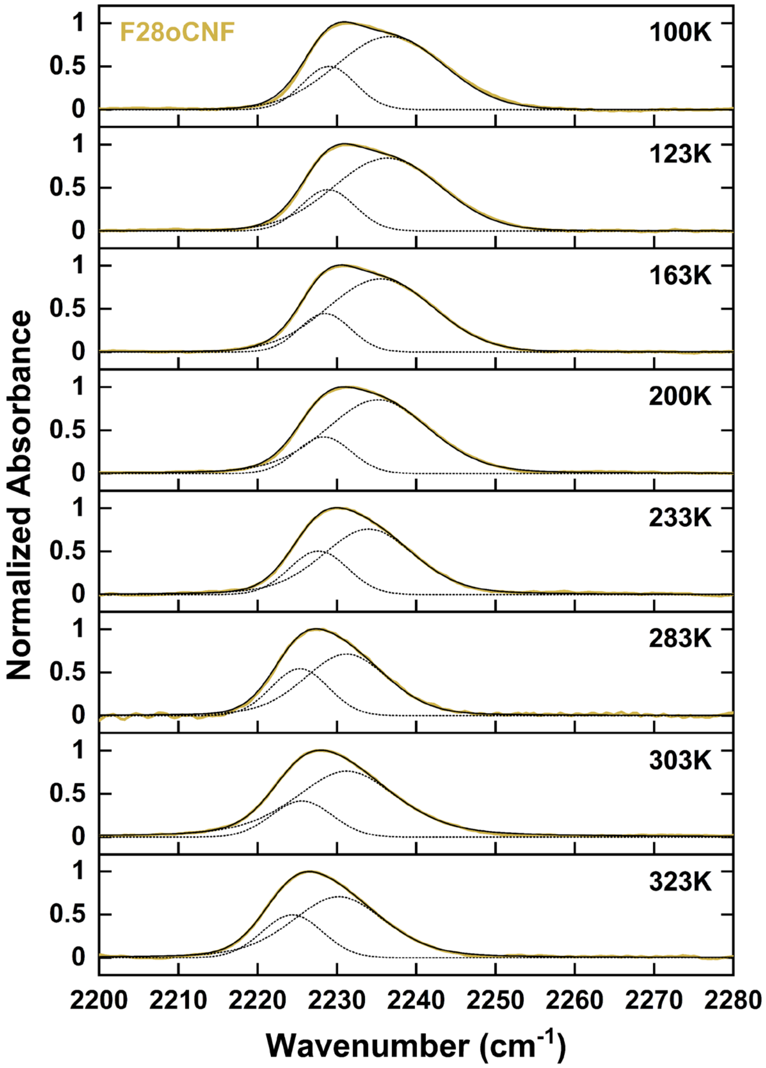


**Figure S14.** F28oCNF PYP spectra in 1:1 mixtures of glycerol and aqueous buffer for temperatures ranging from 100 K to 323 K. Experimental data are shown in gold, total fits are shown as solid black lines, and fits to individual populations are shown as dotted black lines. Unlike in the room temperature IR in buffer (**Figure S11**),[1] two bands are observed at 303 K (our closest temperature to room temperature). The high glycerol content will alter water's dynamics, and since water is F28oCNF's H-bond partner (in the crystal structure[1] and in the MD, see **Table S15**), one explanation for this difference is that the exchange of the nitrile's H-bonding/non-H-bonding populations is sufficiently slowed so that the individual populations are observable.

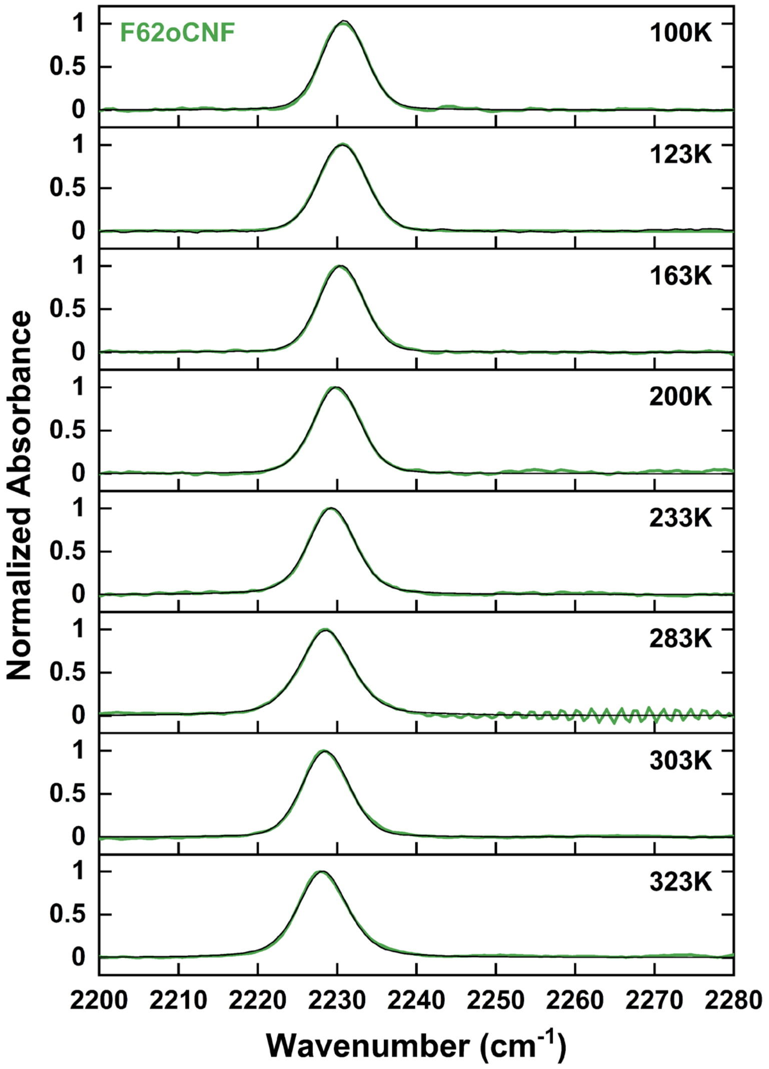


**Figure S15.** F62oCNF PYP spectra in 1:1 mixtures of glycerol and aqueous buffer for temperatures ranging from 100 K to 323 K. Experimental data are shown in green and fits are shown as solid black lines. Spectra across the entire temperature range are well fit by a single band.

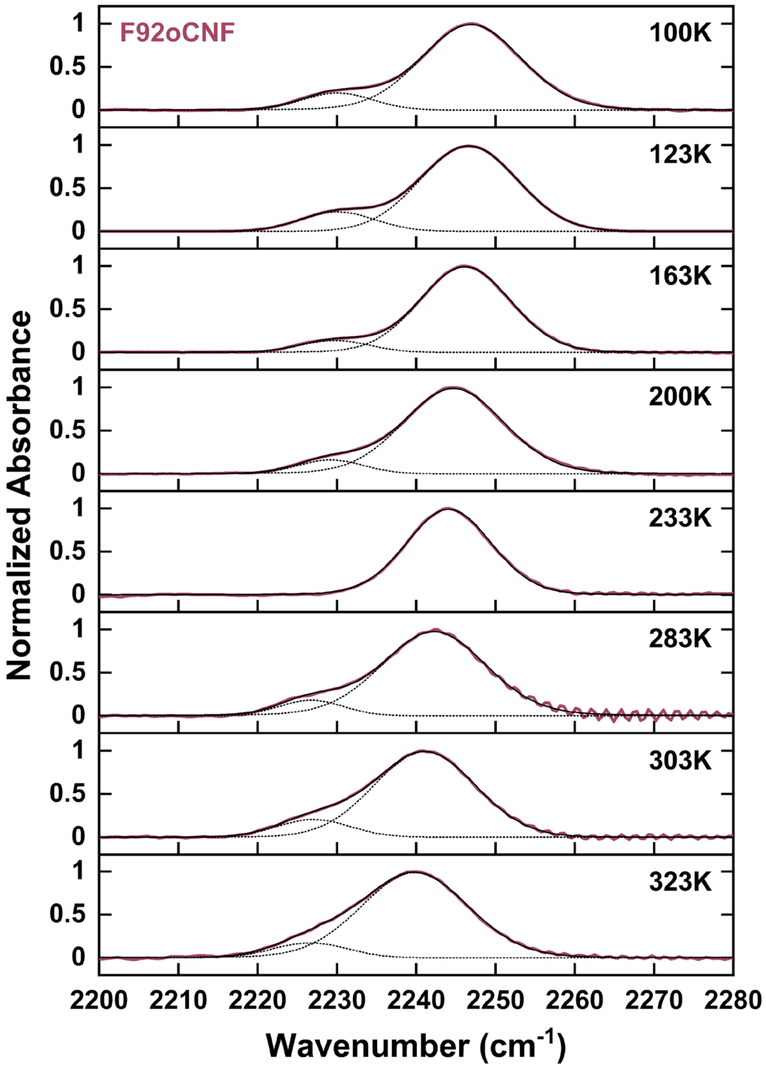


**Figure S16.** F92oCNF PYP spectra in 1:1 mixtures of glycerol and aqueous buffer for temperatures ranging from 100 K to 323 K. Experimental data are shown in red, total fits are shown as solid black lines, and fits to individual populations are shown as dotted black lines. Just as for F28oCNF (**Figure S14**), two bands are observed at 303 K (our closest temperature to room temperature), while a single band was observed for the room temperature IR of F92oCNF in buffer (**Figure S11**).[1] However, unlike F28oCNF, T90 (*i.e.*, the protein) appears to be the main H-bond donor for F92oCNF in the crystal structure[1] and in the POL MD (**Table S15**). It was observed in the MD simulations that when F92oCNF and T90 were not H-bonding, T90 would H-bond with the solvent. In that case, one hypothesis for the appearance of two bands in the 303 K (and 283 K and 323 K) spectra is that T90 has longer residence times with F92oCNF and with the solvent due to the presence of glycerol such that the two underlying populations are no longer averaged.

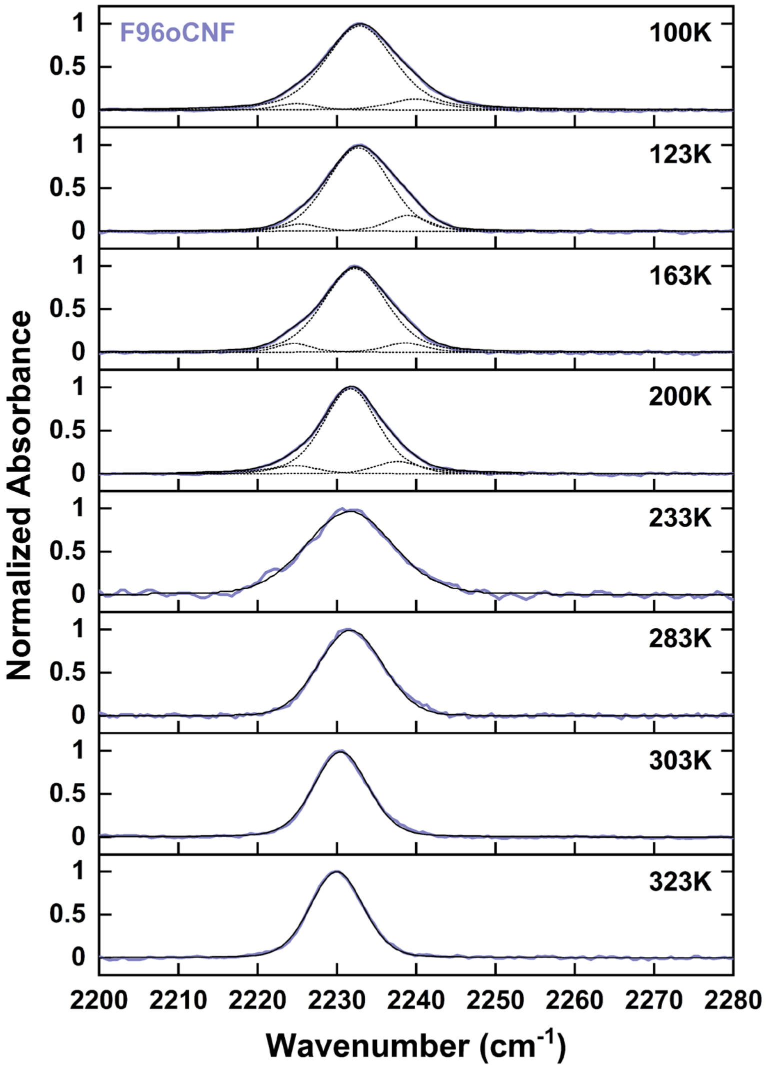


**Figure S17.** F96oCNF PYP spectra in 1:1 mixtures of glycerol and aqueous buffer for temperatures ranging from 100 K to 323 K. Experimental data are shown in blue, total fits are shown as solid black lines, and fits to individual populations are shown as dotted black lines. Unlike F28oCNF and F92oCNF (and like F96oCNF's room temperature IR in **Figure S11**),[1] a single band is observed for spectra near room temperature. However, the spectra narrow from 233 K to 323 K (**Table S12**), which is consistent with the spectral behavior for species passing from the intermediate to fast exchange regimes.

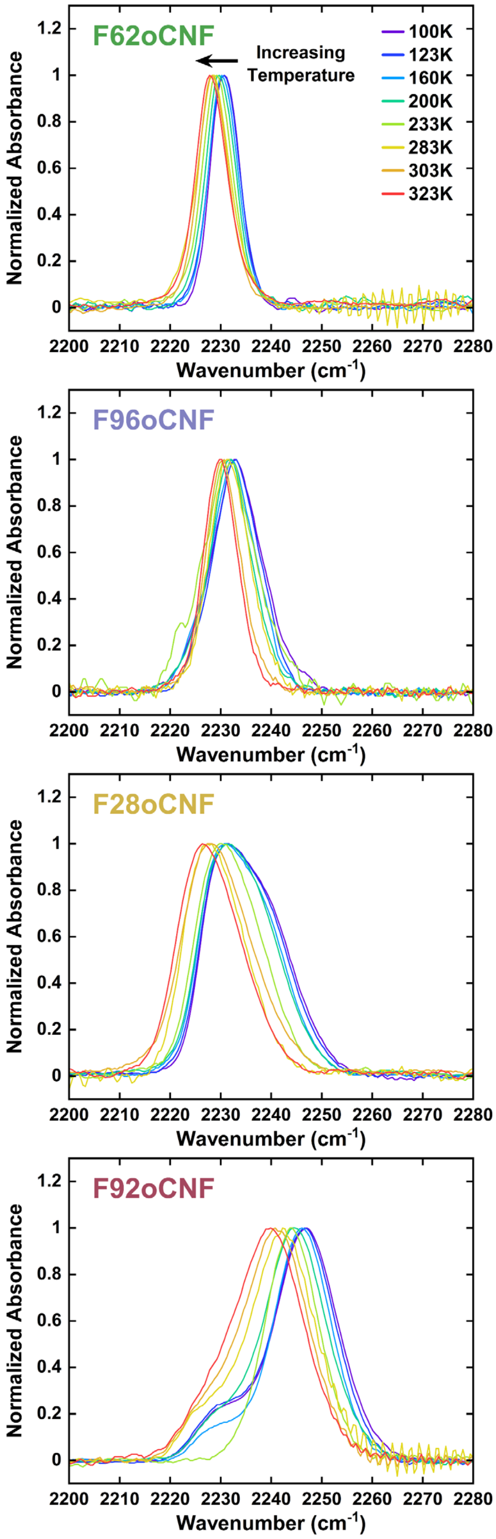


**Figure S18.** Temperature-dependent IR spectra for all nitrile containing PYP variants in 1:1 mixtures of glycerol and aqueous buffer. Spectra are shown in order of increasing H-bonding blueshifts (for values obtained at room temperature in buffer)[1] from top to bottom.

*Characterization of Temperature-Dependent IR Spectra*

**Table S12.** Extracted IR peak parameters (frequencies and FWHM) observed for F28oCNF, F62oCNF, F92oCNF, and F96oCNF nitriles measured at various temperatures in 1:1 mixtures of glycerol and aqueous buffer.

| PYP Construct | 100 K Freq. ($cm^{-1}$)/ FWHM ($cm^{-1}$) | 123 K Freq. ($cm^{-1}$)/ FWHM ($cm^{-1}$) | 160 K Freq. ($cm^{-1}$)/ FWHM ($cm^{-1}$) | 200 K Freq. ($cm^{-1}$)/ FWHM ($cm^{-1}$) | 233 K Freq. ($cm^{-1}$)/ FWHM ($cm^{-1}$) | 283 K Freq. ($cm^{-1}$)/ FWHM ($cm^{-1}$) | 303 K Freq. ($cm^{-1}$)/ FWHM ($cm^{-1}$) | 323 K Freq. ($cm^{-1}$)/ FWHM ($cm^{-1}$) |
|---|---|---|---|---|---|---|---|---|
| F28oCNF (low energy) | 2229.0/ 7.9 | 2228.9/ 8.0 | 2228.5/ 7.7 | 2228.3/ 8.1 | 2227.7/ 8.6 | 2225.4/ 8.5 | 2225.6/ 9.0 | 2224.4/ 9.0 |
| F28oCNF (high energy) | 2236.7/ 16.1 | 2236.4/ 16.0 | 2235.5/ 15.9 | 2235.2/ 15.4 | 2234.0/ 13.8 | 2231.5/ 12.6 | 2231.2/ 15.4 | 2230.2/ 14.0 |
| F62oCNF | 2230.8/ 6.6 | 2230.7/ 7.1 | 2230.5/ 6.7 | 2229.9/ 6.9 | 2229.3/ 7.0 | 2228.6/ 7.7 | 2228.5/ 7.0 | 2228.2/ 7.3 |
| F92oCNF (low energy) | 2230.1/ 10.4 | 2230.1/ 11.0 | 2229.7/ 10.1 | 2229.3/ 9.7 | – | 2226.7/ 9.5 | 2227.1/ 10.8 | 2226.6/ 11.0 |
| F92oCNF (high energy) | 2246.9/ 14.9 | 2246.6/ 14.4 | 2246.2/ 13.5 | 2244.7/ 14.9 | 2244.1/ 12.6 | 2242.2/ 15.5 | 2241.1/ 15.0 | 2239.8/ 15.9 |
| F96oCNF (lowest energy) | 2224.9/ 5.7 | 2225.4/ 5.8 | 2224.6/ 5.3 | 2224.7/ 6.7 | – | – | – | – |
| F96oCNF (medium energy) | 2232.9/ 10.3 | 2232.7/ 9.3 | 2232.2/ 9.5 | 2231.7/ 8.1 | 2231.6/ 12.9 | 2231.7/ 9.5 | 2230.5/ 8.1 | 2230.0/ 7.9 |
| F96oCNF (highest energy) | 2239.9/ 7.7 | 2239.0/ 6.3 | 2238.6/ 6.2 | 2237.6/ 6.5 | – | – | – | – |

## S6 FC MD vs POL MD Electric Field Correlations

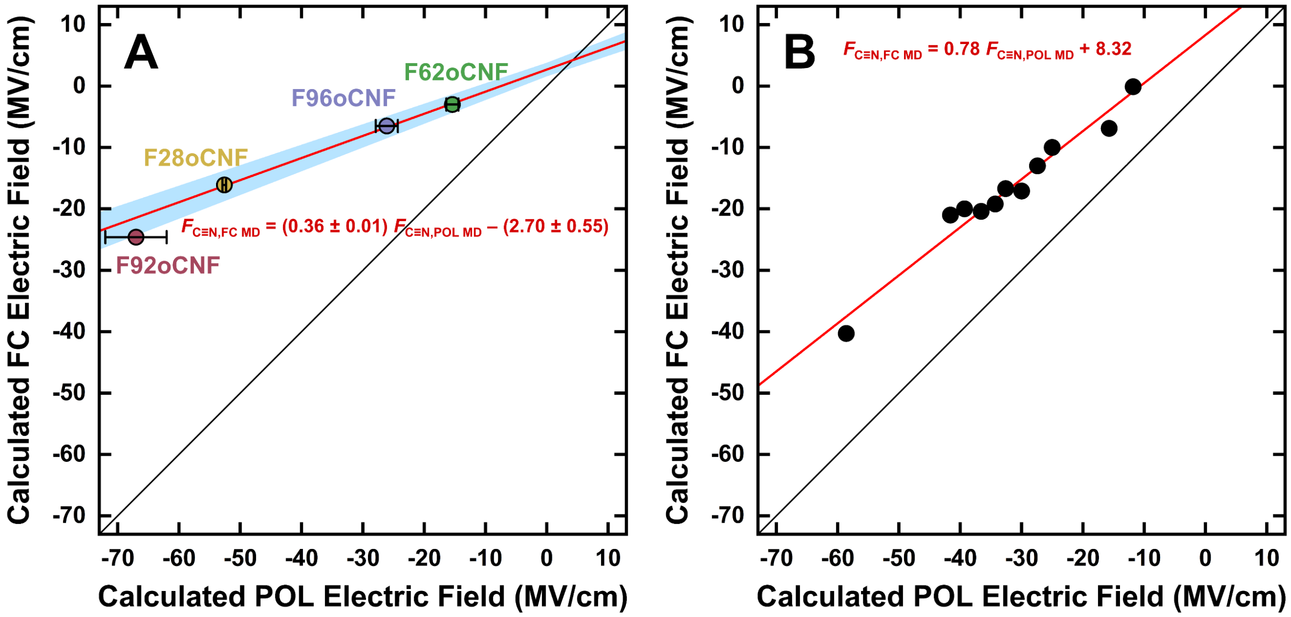


**Figure S19.** Nitrile electric fields from FC MD vs electric fields from POL MD for (A) PYP variants and (B) oTN in solvents.[1] The red lines are the best fits, and the fit in (A) uses an error-weighted linear regression.[40,41] The blue region in (A) is the 2σ confidence interval for the regression. The black lines indicate perfect correlation. In (A), error bars for FC MD are smaller than the size of the symbol and are therefore suppressed for visualization purposes.

Linear regression of the FC vs POL MD fraction-weighted electric fields ($F_{C≡N,FC\ MD}$'s vs $F_{C≡N,POL\ MD}$'s) for the PYP variants yields a 2σ confidence interval for the slope of 0.34 – 0.38 (**Figure S19A**); this is in the range of the ratio of fields calculated with nonpolarizable and polarizable methods for the carbonyl in the active site of ketosteroid isomerase and mutants (0.3 – 0.7),[42–44] even though fields sampled here are much smaller and the vibrational probe is different. In contrast, the values for the 2σ confidence interval in **Figure S19A** are approximately half the value of the analogous regression slope of 0.78 for $F_{C≡N,FC\ MD}$'s vs $F_{C≡N,POL\ MD}$'s of oTN in solvents (**Figure S19B**).[1] To provide some context for this value, we compared the ratios of FC and POL MD fields for all carbonyl- or nitrile-bearing small molecules in solvents that we are aware of, and we found the range of ratios to be 0.75 – 1.0.[1,2,16,45] Thus, the FC to POL MD electric field ratios are consistently smaller in proteins than in solvents.

## S7 MD Nitrile H-Bonding Characterization

*Nitrile H-Bond Contour Plots*

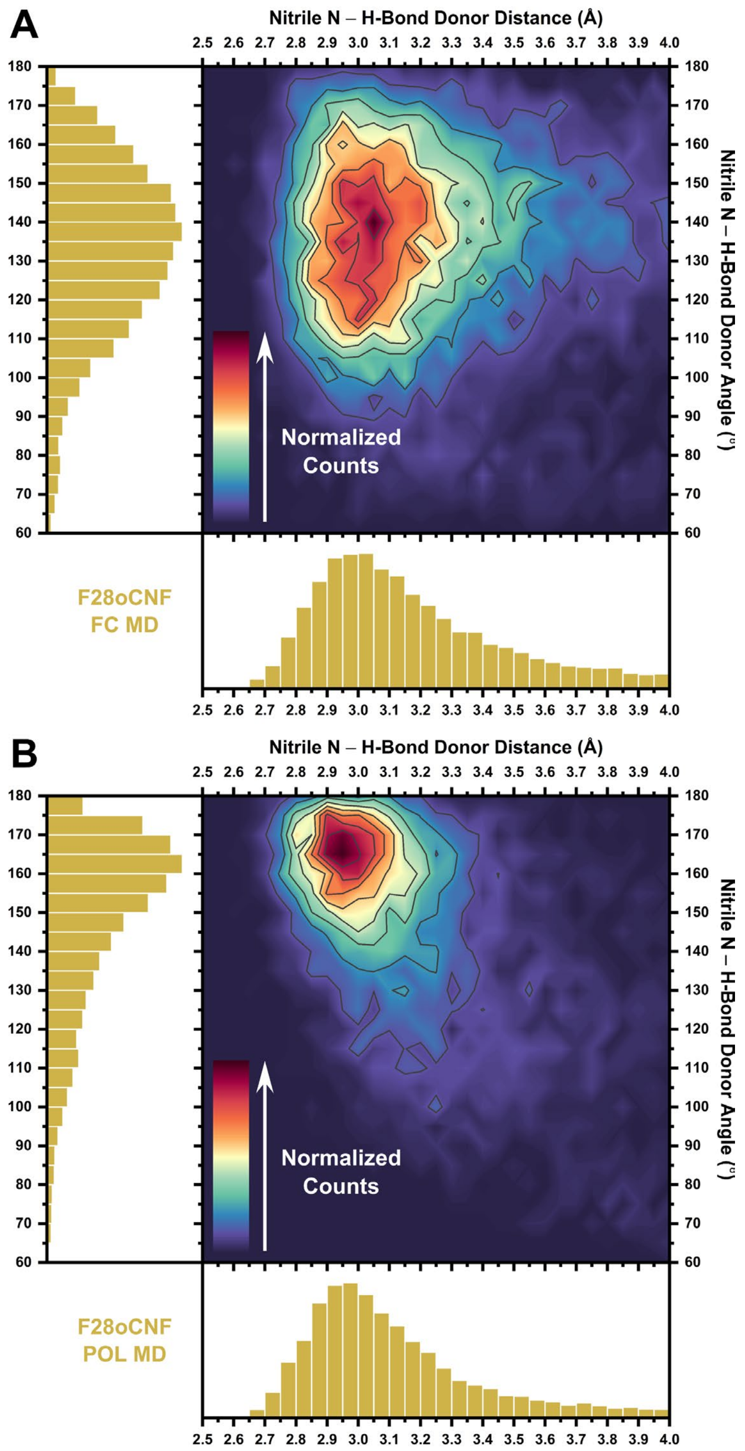


**Figure S20.** Nitrile H-bond angle vs distance in F28oCNF PYP (A) FC MD or (B) POL MD depicted as a contour plot describing H-bond angle/distance sampling space. 1D histograms show the sampling space projected along the individual variables.

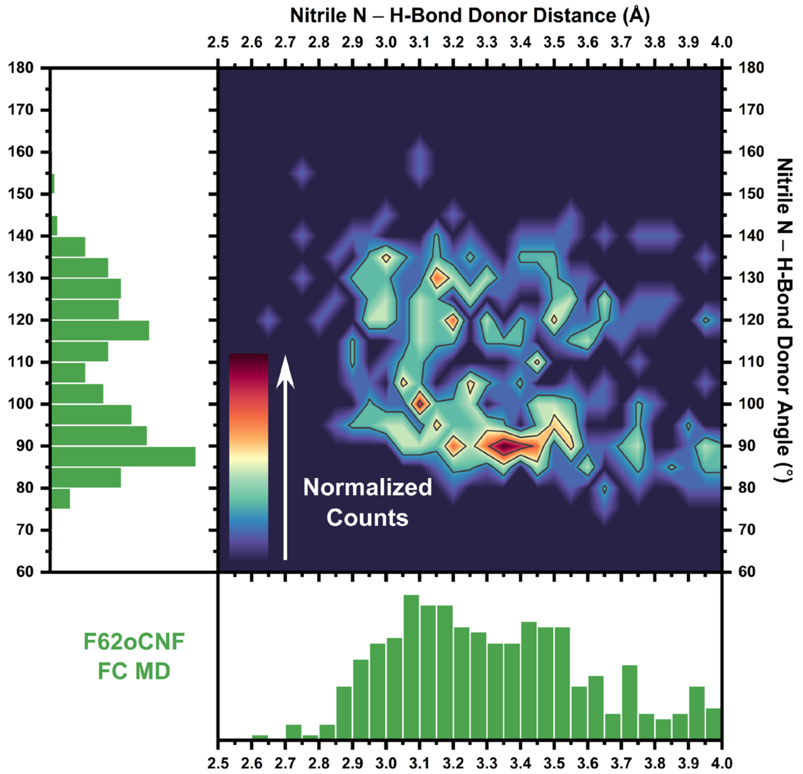


**Figure S21.** Nitrile H-bond angle vs distance in F62oCNF PYP FC MD depicted as a contour plot describing H-bond angle/distance sampling space; no H-bonds were observed for the nitrile in POL MD. 1D histograms show the sampling space projected along the individual variables.

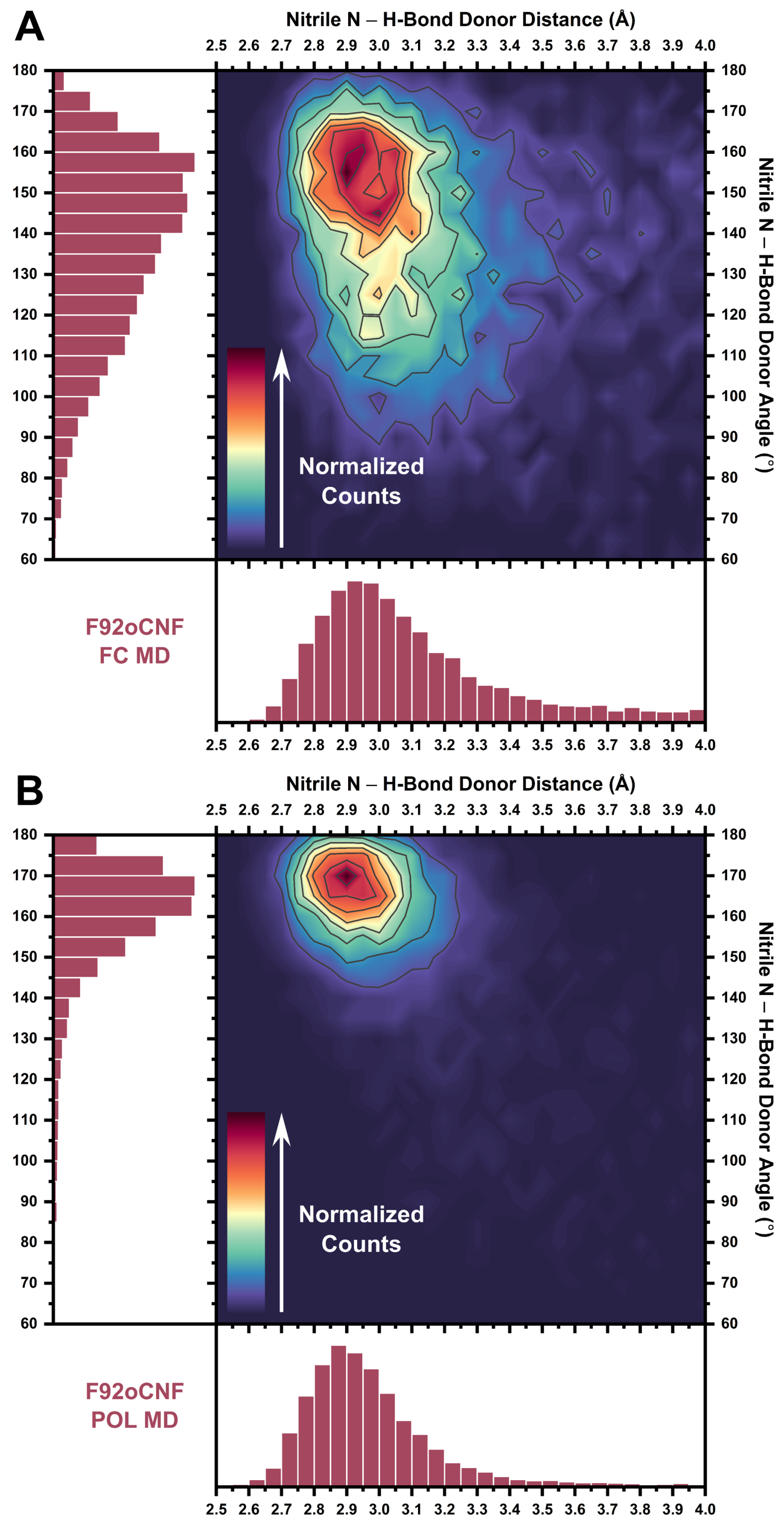


**Figure S22.** Nitrile H-bond angle vs distance in F92oCNF PYP (A) FC MD or (B) POL MD depicted as a contour plot describing H-bond angle/distance sampling space. 1D histograms show the sampling space projected along the individual variables.

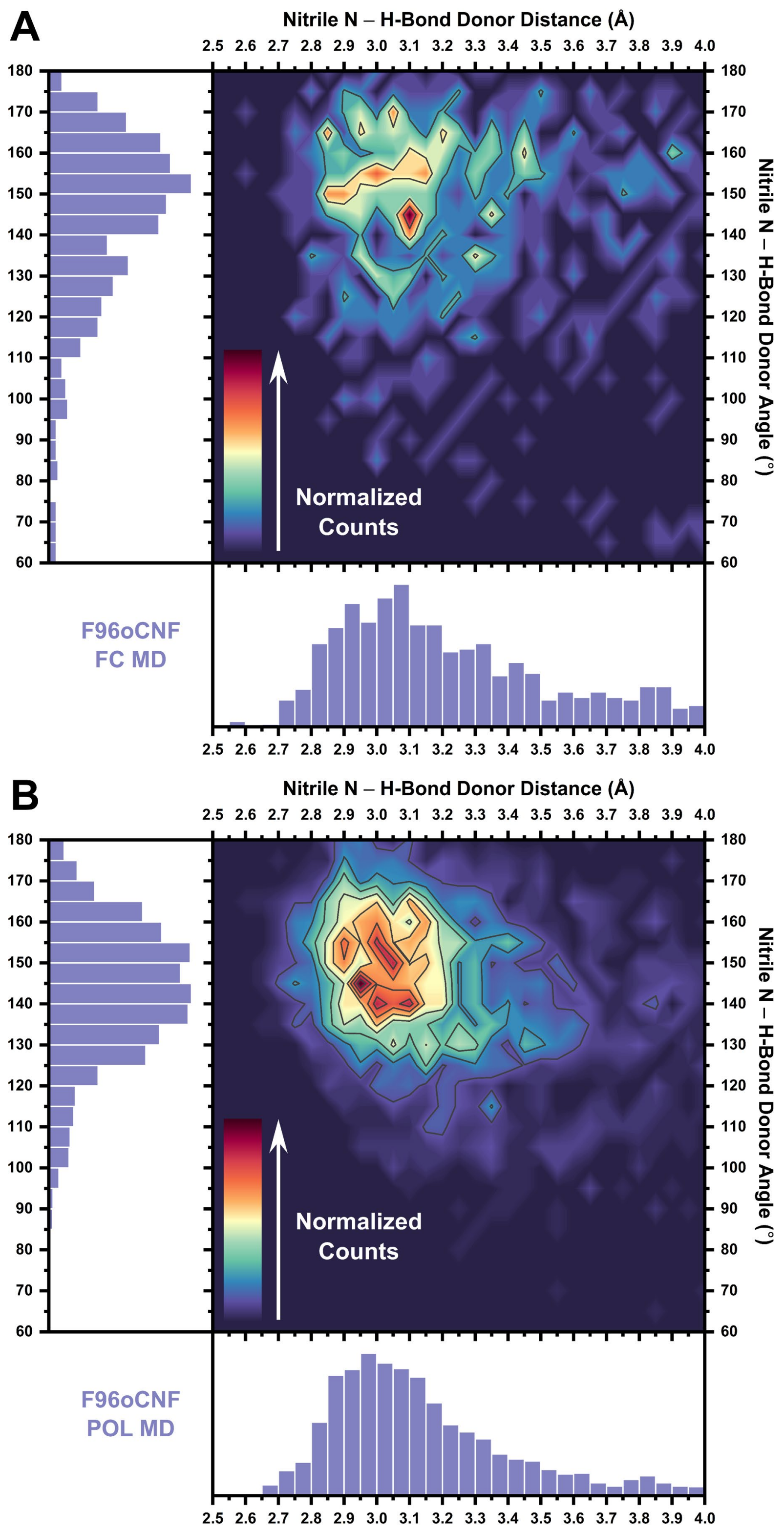


**Figure S23.** Nitrile H-bond angle vs distance in F96oCNF PYP (A) FC MD or (B) POL MD depicted as a contour plot describing H-bond angle/distance sampling space. 1D histograms show the sampling space projected along the individual variables.

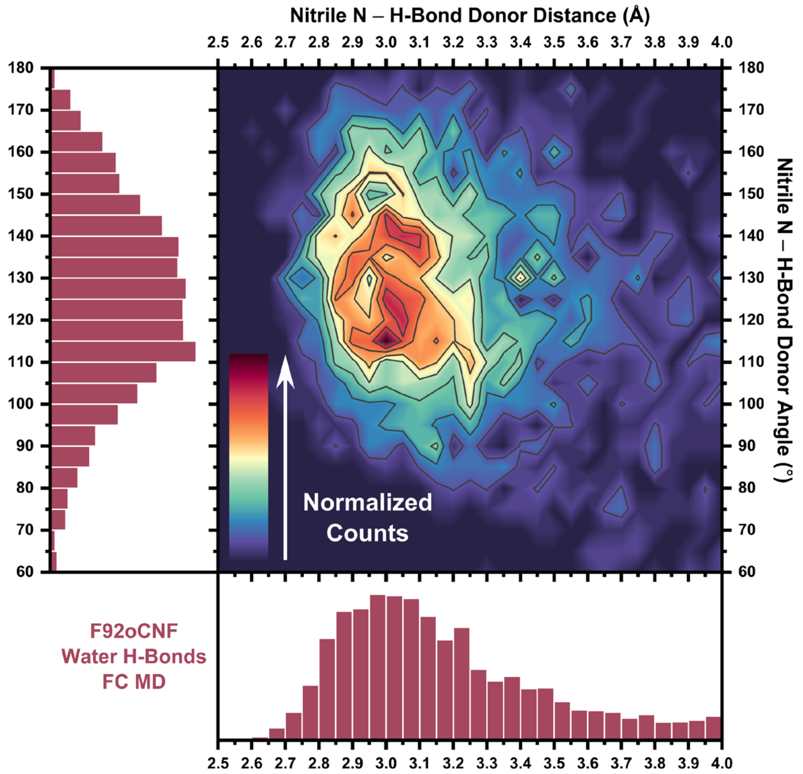


**Figure S24.** Nitrile H-bond angle vs distance for F92oCNF's H-bonds with water in F92oCNF PYP FC MD depicted as a contour plot describing H-bond angle/distance sampling space. 1D histograms show the sampling space projected along the individual variables.

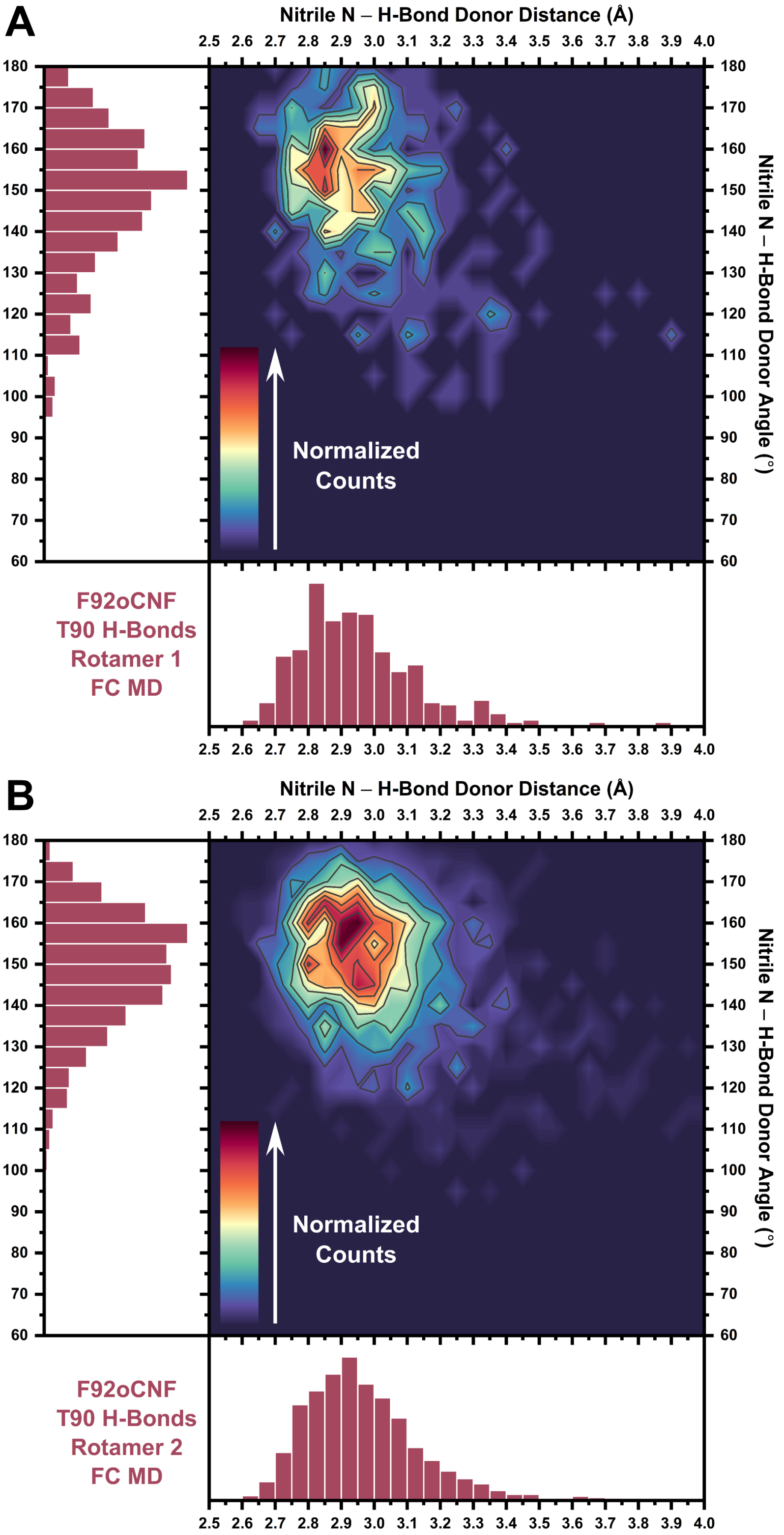


**Figure S25.** Nitrile H-bond angle vs distance for F92oCNF's H-bonds with T90's sidechain hydroxyl in F92oCNF PYP FC MD depicted as contour plots describing H-bond angle/distance sampling space. 1D histograms show the sampling space projected along the individual variables. Two plots were generated to account for the distinct rotamers in the average MD structures for F92oCNF H-bonding with T90 (**Figure S27**).

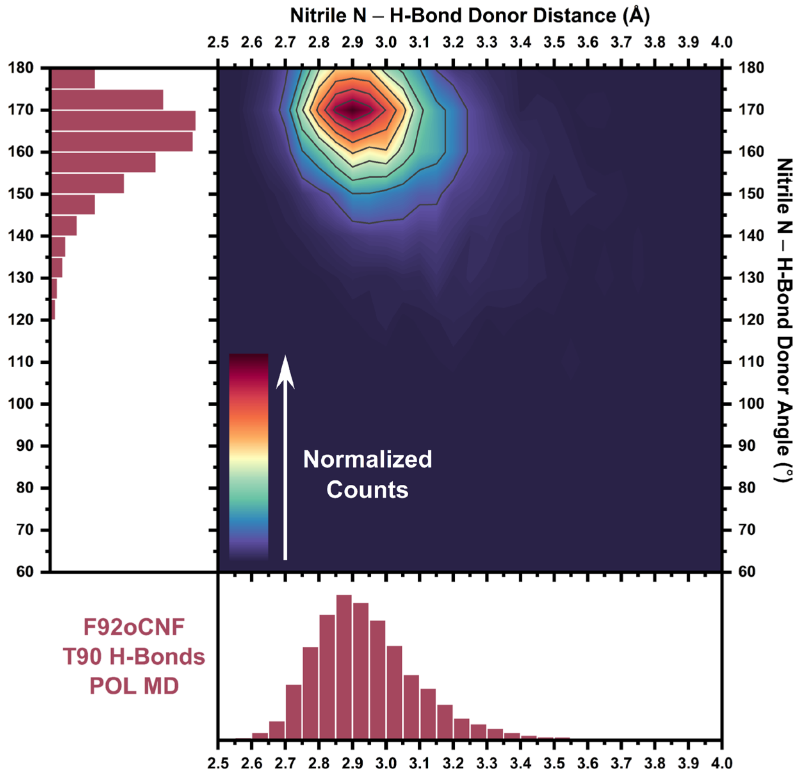


**Figure S26.** Nitrile H-bond angle vs distance for F92oCNF's H-bonds with T90's sidechain hydroxyl in F92oCNF PYP POL MD depicted as a contour plot describing H-bond angle/distance sampling space. 1D histograms show the sampling space projected along the individual variables.

*Nitrile H-bond Distances and Angles Extracted from Contour Plots*

To extract average H-bond donor distances and angles for the contour plots in **Figures S20-S25**, the plots were fit with a rotated Gaussian surface (*i.e.*, 2D Gaussian):

$$C = A\,exp\left\{-\frac{1}{2}\left(\frac{d_{NX}cos(\varphi) + \theta_{CNX}sin(\varphi) - \mu_{d_{NX}}cos(\varphi) + \mu_{\theta_{CNX}}sin(\varphi)}{\sigma_{d_{NX}}}\right)^2 - \frac{1}{2}\left(\frac{-d_{NX}sin(\varphi) + \theta_{CNX}cos(\varphi) - \mu_{d_{NX}}sin(\varphi) + \mu_{\theta_{CNX}}cos(\varphi)}{\sigma_{\theta_{CNX}}}\right)^2\right\}$$

where *C* is the relative number of counts, *A* is a scaling factor, $d_{NX}$ is the H-bond donor distance, $\theta_{CNX}$ is the H-bond donor angle, $\varphi$ is the rotation angle in the plane of the contour plot, $\mu_{d_{NX}}$ is the H-bond distance mean, $\mu_{\theta_{CNX}}$ is the H-bond angle mean, $\sigma_{d_{NX}}$ is the H-bond donor standard deviation, and $\sigma_{\theta_{CNX}}$ is the H-bond angle standard deviation (**Table S13**). Values for fits to F62oCNF's and F96oCNF's contour plots with FC MD were unreliable due to low counts and are not reported.

**Table S13.** Average nitrile H-bond distances and angles from fits with rotated Gaussian surfaces. Errors provided are 1σ standard errors of the fit.

| Environment/ Force Field | Avg. H-Bond Distance, $\mu_{d_{NX}}$ (Å) | Avg. H-Bond Angle, $\mu_{\theta_{CNX}}$ (°) | H-Bond Distance s.d., $\sigma_{d_{NX}}$ (Å) | H-Bond Angle s.d., $\sigma_{\theta_{CNX}}$ (°) | $R^2$ |
|---|---|---|---|---|---|
| F28oCNF/FC | 3.08 ± 0.003 | 137.4 ± 0.3 | 0.22 ± 0.003 | 23.2 ± 0.4 | 0.88 |
| F28oCNF/POL | 3.00 ± 0.003 | 162.9 ± 0.3 | 0.15 ± 0.003 | 16.4 ± 0.4 | 0.88 |
| F92oCNF/FC | 2.99 ± 0.003 | 146.0 ± 0.5 | 0.17 ± 0.003 | 24.3 ± 0.5 | 0.85 |
| F92oCNF/POL | 2.93 ± 0.001 | 167.2 ± 0.1 | 0.13 ± 0.001 | 9.9 ± 0.1 | 0.96 |
| F96oCNF/POL | 3.05 ± 0.003 | 148.4 ± 0.3 | 0.18 ± 0.007 | 15.2 ± 0.3 | 0.86 |
| $F92oCNF_{Water}$/FC | 3.07 ± 0.004 | 128.8 ± 0.5 | 0.21 ± 0.004 | 23.9 ± 0.5 | 0.81 |
| $F92oCNF_{T90, Rotamer 1}$/FC[a,b] | 2.91 ± 0.003 | 154.3 ± 0.4 | 0.14 ± 0.003 | 14.6 ± 0.4 | 0.75 |
| $F92oCNF_{T90, Rotamer 2}$/FC[a] | 2.95 ± 0.002 | 153.6 ± 0.2 | 0.15 ± 0.002 | 13.4 ± 0.2 | 0.91 |
| $F92oCNF_{T90}$/POL | 2.93 ± 0.001 | 169.6 ± 0.1 | 0.13 ± 0.001 | 10.5 ± 0.1 | 0.98 |

[a] Rotamer identities are defined in **Figure S27**

[b] Rotamer 1 is the rotamer displayed in main text **Figure 5G**

We also calculated the average, standard deviation of the average, and median of the individual H-bond distance and angle variables for comparison with values from simultaneous fitting (*i.e.*, fits with the 2D Gaussians). We find average H-bond distances and angles are over- and underestimated, respectively, compared to the values from simultaneous fitting, while all standard deviations are overestimated. Median values are closer to values from simultaneous fitting but still over- and under-estimated; they are likely closer than the averages to the values from the rotated Gaussian fits because of the asymmetries to the H-bonding variables' histograms from tails at longer distances and shallower angles (**Figures S20-S23**).

**Table S14.** Average, standard deviation of the average, and median nitrile H-bond distances and angles from statistics on individual variables (*i.e.*, not simultaneously characterized).

| Environment/ Force Field | Avg. H-Bond Distance (Å) | Avg. H-Bond Angle (°) | H-Bond Distance s.d. (Å) | H-Bond Angle s.d. (°) | Med. H-Bond Distance (Å) | Med. H-Bond Angle (°) |
|---|---|---|---|---|---|---|
| F28oCNF/POL | 3.10 | 146.5 | 0.27 | 23.8 | 3.04 | 153.4 |
| F28oCNF/FC | 3.20 | 132.4 | 0.29 | 22.5 | 3.09 | 134.3 |
| F62oCNF/FC | 3.31 | 106.2 | 0.29 | 18.4 | 3.27 | 103.3 |
| F92oCNF/FC | 3.08 | 135.9 | 0.29 | 23.4 | 3.02 | 139.9 |
| F92oCNF/POL | 2.98 | 157.2 | 0.21 | 18.3 | 2.93 | 162.0 |
| F96oCNF/FC | 3.21 | 141.9 | 0.32 | 22.7 | 3.13 | 146.8 |
| F96oCNF/POL | 3.13 | 142.4 | 0.27 | 17.2 | 3.08 | 143.5 |

*MD H-Bond Donor Identities*

**Table S15.** Fraction of nitrile H-bonds to either a protein residue or solvent from MD simulations.

| Environment/ Force Field | Protein (%) | Solvent (%) |
|---|---|---|
| F28oCNF/FC | 9.1 | 90.9 |
| F28oCNF/POL | 4.9 | 95.1 |
| F62oCNF/FC | < 0.1 | > 99.9 |
| F62oCNF/POL | N/A | N/A |
| F92oCNF/FC[a] | 50.2 (81.3) | 49.8 |
| F92oCNF/POL[a] | 95.2 (94.3) | 4.8 |
| F96oCNF/FC | 4.7 | 95.3 |
| F96oCNF/POL | 2.0 | 98.0 |

[a] In F92oCNF PYP, T90's sidechain hydroxyl was observed as the closest H-bond donor in the X-ray crystal structure (main text **Figure 1E**);[1] as such, the fraction of nitrile–protein H-bonds with T90 is shown in parentheses.

*Average MD Structures for F92oCNF H-Bonding with T90*

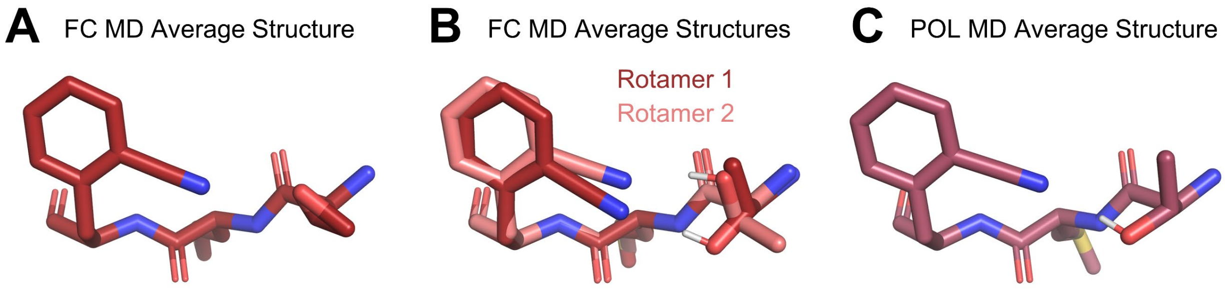


**Figure S27.** Average MD structures for the F92oCNF–T90 H-bond. (A) Average structure from FC MD assuming a single T90 rotamer. (B) Average structures from FC MD assuming two T90 rotamers. (C) Average structure from POL MD assuming a single T90 rotamer.

Average MD structures for the F92oCNF–T90 H-bond were generated by a script which aligned and averaged all frames in which F92oCNF and T90 form a hydrogen bond. At the outset, the script assumes all residues adopt a single orientation. As can be seen in **Figure S27A**, when this is not the case, residues adopt an obviously incorrect "squished" shape due to the averaging of categorically distinct poses. In this case, a second script was run which analyzed T90's methyl sidechain dihedral (where the dihedral χ is defined by carbonyl C, $C_\alpha$, $C_\beta$, and $C_\gamma$), and we observed an approximately bimodal distribution for χ, with distinct distributions observed from -180° to 10° and from 10° to 180°. The same script then separated the MD trajectory into two trajectories according to the distribution over χ. The average MD structure script was run on each sub-trajectory to generate the average structures for the two rotamer classes, termed "Rotamer 1" and "Rotamer 2" and shown **Figure S27B**. When the average MD structure script was run for POL MD, a single T90 orientation was observed, displayed in **Figure S27C**.

The rotamer observed for POL MD (corresponding with Rotamer 1 in **Figure S27B**) is the same one adopted in the crystal structure (compare with main text **Figure 1E**).[1] In FC MD, Rotamer 1 and Rotamer 2 are observed at an approximately 1:4 ratio, indicating T90 frequently moves away from its starting structure in the crystal configuration with the AMBER FF. Interestingly, contour plots for F92oCNF–T90 H-bond sampling spaces with FC MD (**Figure S25**) indicate the two rotamers possess highly similar average H-bond distances and angles (2.91 Å and 154° for Rotamer 1 and 2.95 Å and 154° for Rotamer 2; **Table S13**), though it should be noted Rotamer 1 is observed somewhat infrequently over the course of the simulations, indicated by the roughness of the distribution in **Figure S25A**. Both rotamers adopt a smaller average H-bond angle than the POL MD rotamer (**Table S13**; also, illustrated in main text **Figure 5G,H**), the origins for which are discussed in the main text.

Note, the scripts to generate average MD structures and analyze dihedrals/split MD trajectories can be found at: https://github.com/KozuchLab/Publications/tree/main/oCNPhe_GROMACS_TINKER

## S8 oTN Electrostatic Potential (ESP) Maps

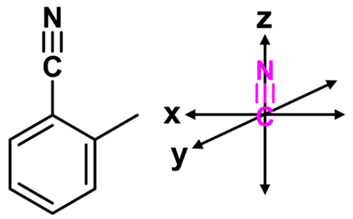


**Figure S28.** oTN model compound and its relative orientation along x, y, and z axes used in main text **Figure 6** and **Figures S29-S31**. The nitrile C is located at the origin.

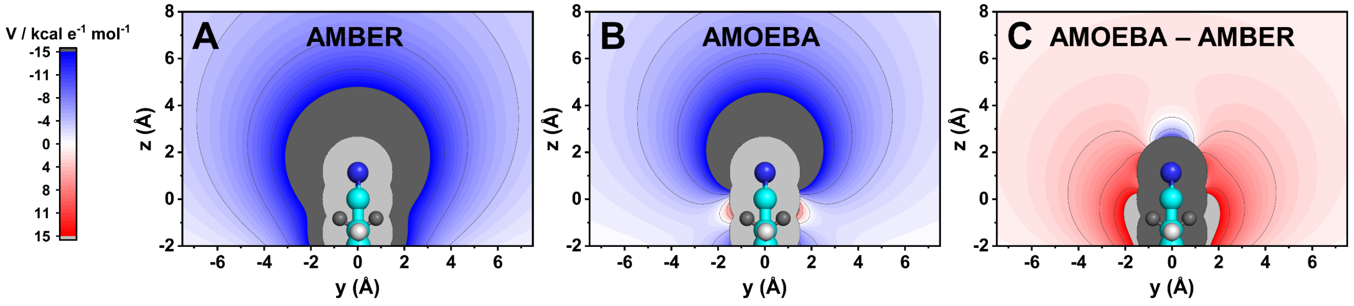


**Figure S29.** ESP maps for oTN in the yz plane. (A) and (B) show the maps for the AMBER FF and the AMOEBA FF, respectively. The AMBER ESP only shows negative potentials in the yz plane, while the AMOEBA ESP shows negative potentials concentrated in front of the nitrile and some positive potentials near the nitrile carbon along the y axis. The AMOEBA minus AMBER difference is shown in (C), where, analogous to main text **Figure 6,** AMOEBA has additional negative potential in front of the nitrile nitrogen and more positive potentials in the rest of the yz plane; in a chemical sense, these differences correspond with additional electron density along the nitrile bond axis.

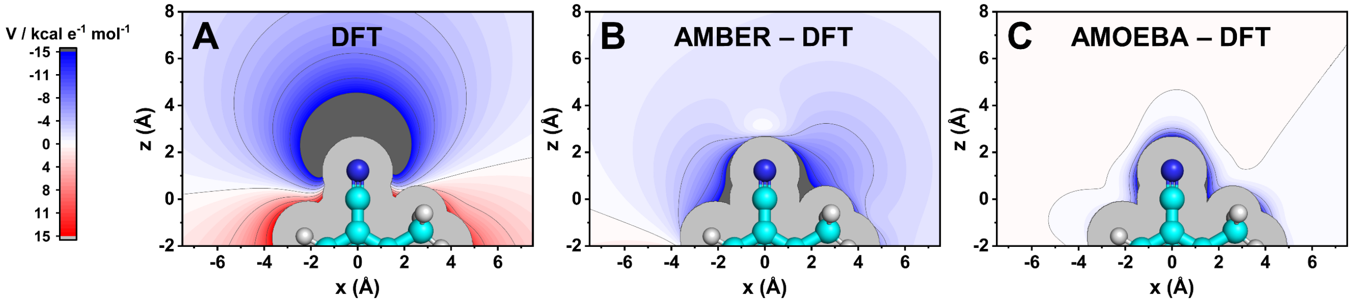


**Figure S30.** (A) ESP map with DFT in the xz plane for oTN. (B) and (C) show difference maps between either AMBER and DFT or AMOEBA and DFT, respectively (AMBER and AMOEBA ESPs in the xz plane are shown in main text **Figure 6**). The difference maps indicate AMOEBA ESPs are significantly closer to DFT potentials than AMBER ESPs. A related result was previously observed that calculated DFT electric fields for an electric field in the active site of the enzyme peptidyl-prolyl isomerase cyclophilin A were better recapitulated with the AMOEBA FF than either the AMBER or CHARMM FFs.[21]

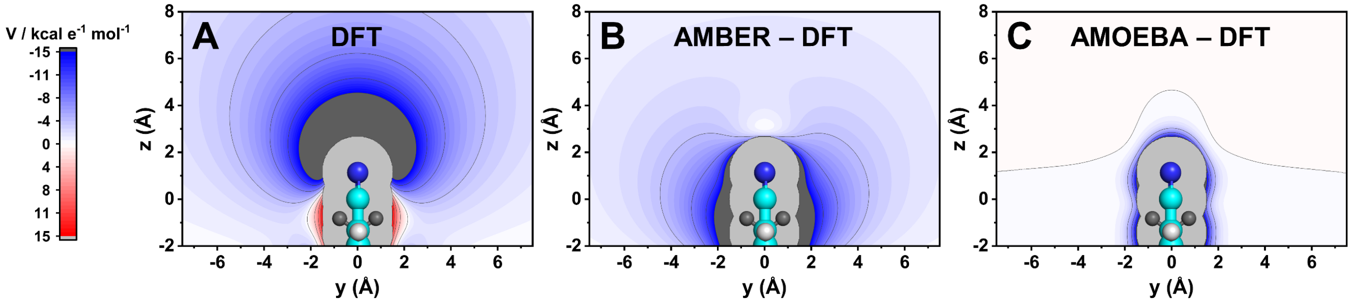


**Figure S31.** (A) ESP map with DFT in the yz plane for oTN. (B) and (C) show difference maps between either AMBER and DFT or AMOEBA and DFT, respectively (AMBER and AMOEBA ESPs in the yz plane are shown in **Figure S29**). The difference maps indicate AMOEBA ESPs are significantly closer to DFT potentials than AMBER ESPs.

## S9 MD Carbonyl H-Bonding Characterization

We chose to study the H-bond sampling for eleven PYP backbone carbonyls to see whether their H-bonding may be FF-dependent like nitriles. Of those eleven, four participate in H-bonding in alphas helices (I11, D20, A45, and F79), three participate in H-bonding in beta sheets (L33, K106, and V120), and three are on loops and H-bond with the solvent (F6, E93, and P102). Within the alpha helix group, two of the carbonyls are solvent exposed (D20 and A45) and two are buried (I11 and F79).

Unlike the nitriles, these carbonyls were present in the simulations of all PYP variants, so H-bonding statistics were taken from all trajectories and pooled. As a lesson from F92oCNF's MD sampling, where FC vs POL MD indicated changes to the primary H-bond donor *and* to the sampling of said donors are possible, we chose to look at a single H-bond donor for each carbonyl from the outset to simplify the analysis and ensure the changes we might observe come from sampling differences. For the alpha helix carbonyls, we observed the H-bond geometries with the residue at the $i$+$4^{th}$ position (*i.e.*, the canonical position for the H-bond donor in alpha helices). For the carbonyls in beta sheets, we visually identified the primary H-bond contact in the neighboring beta sheet from the MD simulations. For the carbonyls on loops, we only considered H-bonds coming from the solvent. Contour plots for the backbone carbonyls in alpha helices, beta sheets, or on loops are shown in **Figures S32-S35**, **Figures S36-S38**, and **Figures S39-S41**, respectively. Average H-bond distances and angles from Gaussian surface fits are provided in **Table S16**.

We found that carbonyls in beta sheets did not experience substantially different H-bonding between the FFs. The average distances did not change by more than 0.03 Å, and the average angle changes across the sites had no clear trend. In contrast, the other carbonyl types demonstrated clearer trends in the average distance and angle when the FF was altered. For the carbonyls in alpha helices at buried sites, comparing from FC to POL MD, we observe increases to the average H-bond distance up to 0.1 Å and decreases to the average angle in the range of 1 – 6°. The changes to alpha helical carbonyls at solvent exposed sites were even larger, with an average distance increase of 0.3 Å from FC to POL MD for A45, and average H-bond angles decreasing about 7 to 8° for both D20 and A45. These changes are the opposite of what was observed for the nitriles, where average distances *decreased* and average angles *increased* from FC to POL MD.

For the carbonyls in loops, the average H-bond distance was not very FF dependent (about 2.8 Å in all cases), but the average H-bond angles again decreased from FC to POL MD, this time with changes of 10 – 15°. However, these values do not fully capture the changes observed, as the POL contour plots had significantly lower $R^2$ values than the FC plots, and the plot for P102 with POL MD in **Figure S41** could not be fit at all. Inspection of **Figure S41** clarifies this point: nearly all H-bond distances from ~ 2.7 Å to 4 Å are appreciably sampled, and the sampling space does not look remotely Gaussian. This long tail of sampling for longer H-bond distances is also observed for F6 and E93, though less prominently. In contrast, the FC MD contour plots are all well fit with high $R^2$ values. While striking, the FF dependence for the carbonyl–water H-bond is perhaps unsurprising, as the challenges of creating realistic water models are well known.[46] However, examples like **Figure S41** highlight the extent of the differences that can be observed, and given the ubiquity of backbone carbonyl H-bonding in folded proteins, a future study comparing their experimentally-derived electric fields with those from MD simulations may be of substantial utility.

*Carbonyl H-Bond Contour Plots*

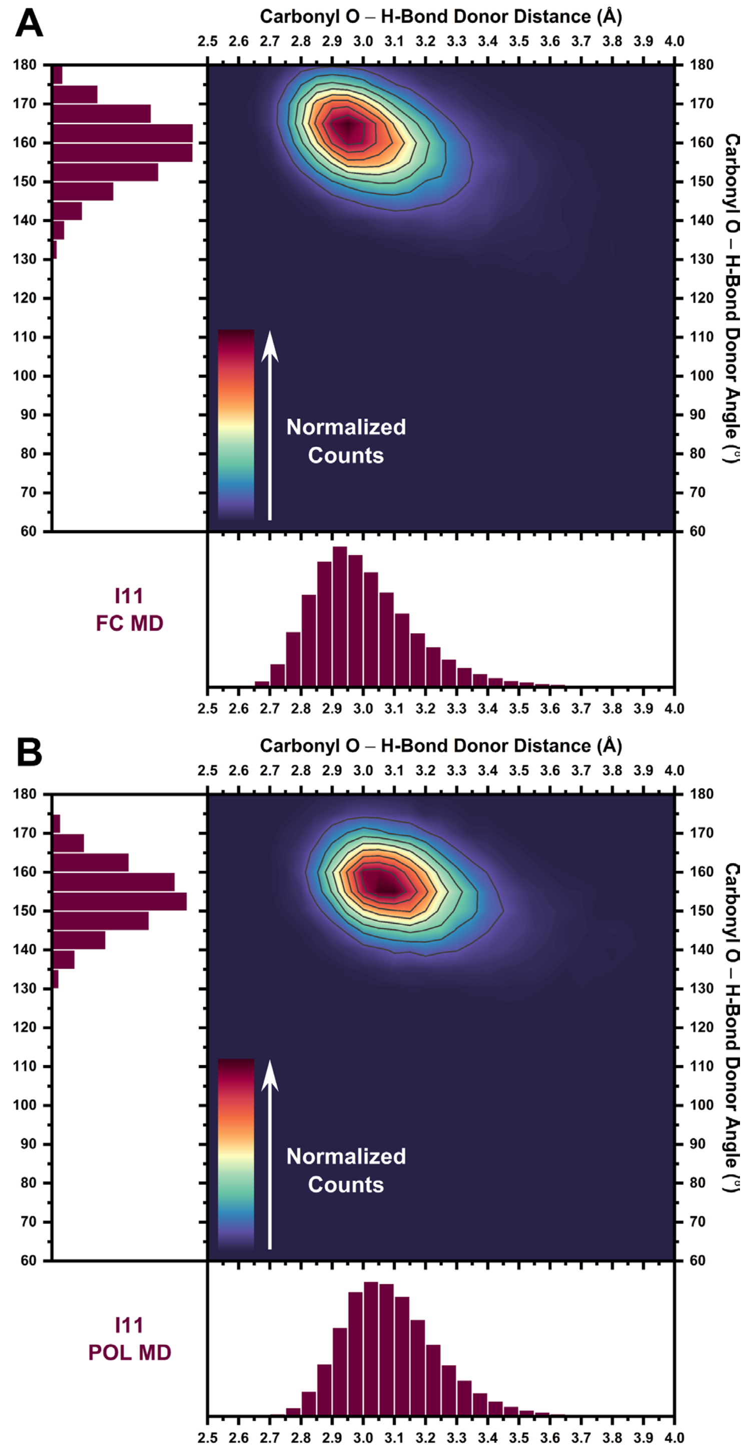


**Figure S32.** Carbonyl H-bond angle vs distance for I11 H-bonds with alpha helix H-bonding partner L15 depicted as a contour plot describing H-bond angle/distance sampling space. 1D histograms show the sampling space projected along the individual variables.

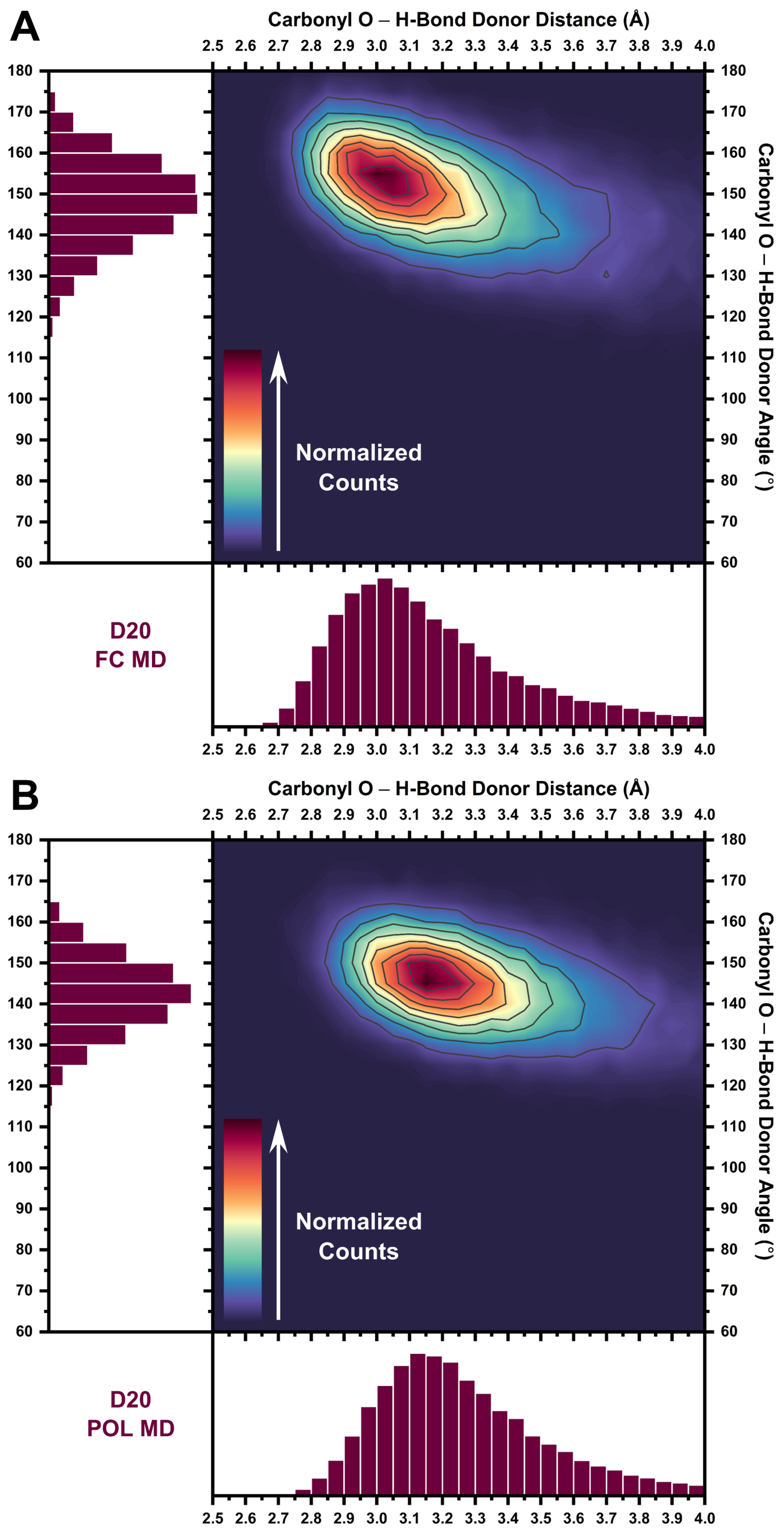


**Figure S33.** Carbonyl H-bond angle vs distance for D20 H-bonds with alpha helix H-bonding partner D24 depicted as a contour plot describing H-bond angle/distance sampling space. 1D histograms show the sampling space projected along the individual variables.

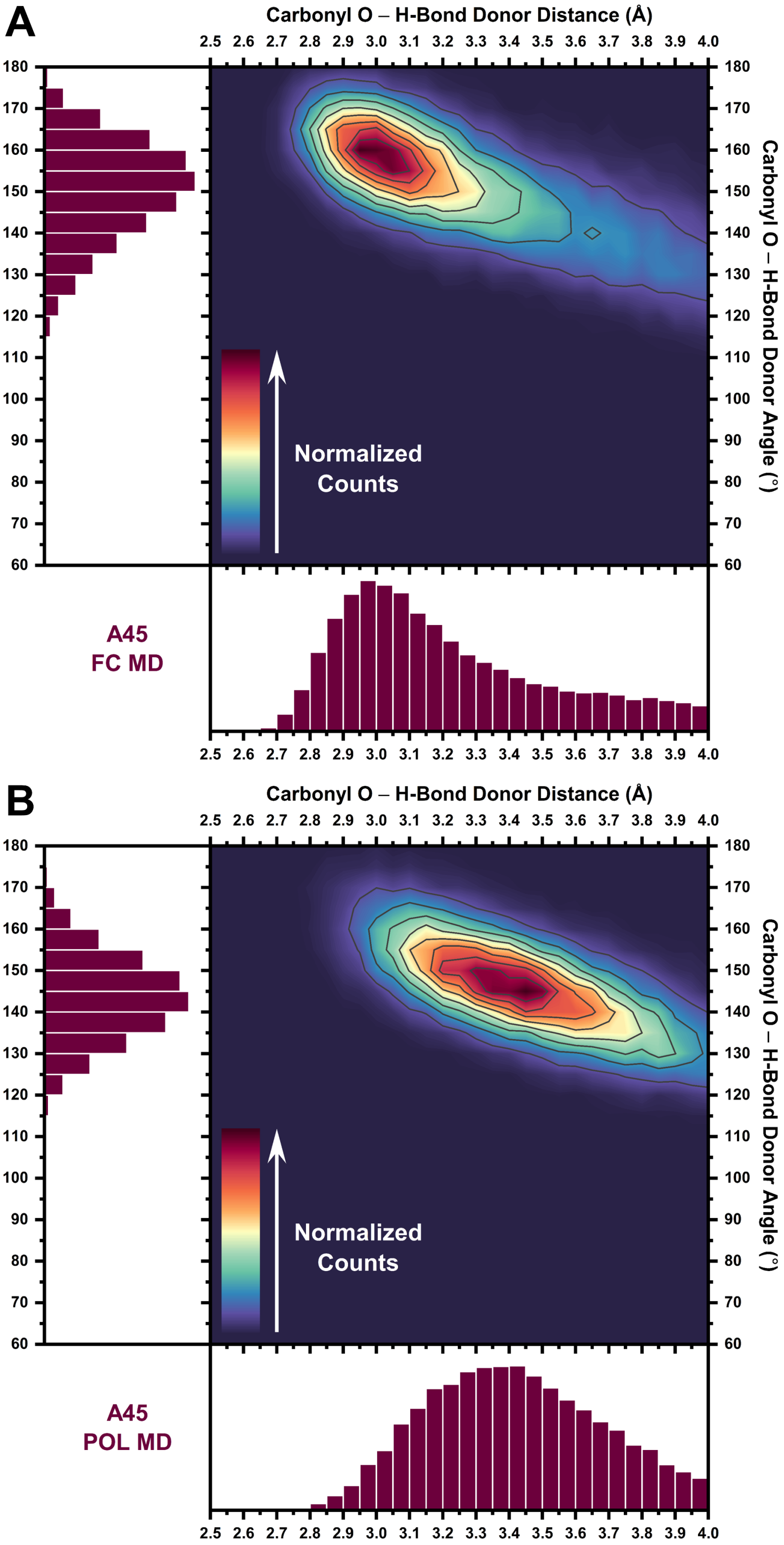


**Figure S34.** Carbonyl H-bond angle vs distance for A45 H-bonds with alpha helix H-bonding partner I49 depicted as a contour plot describing H-bond angle/distance sampling space. 1D histograms show the sampling space projected along the individual variables.

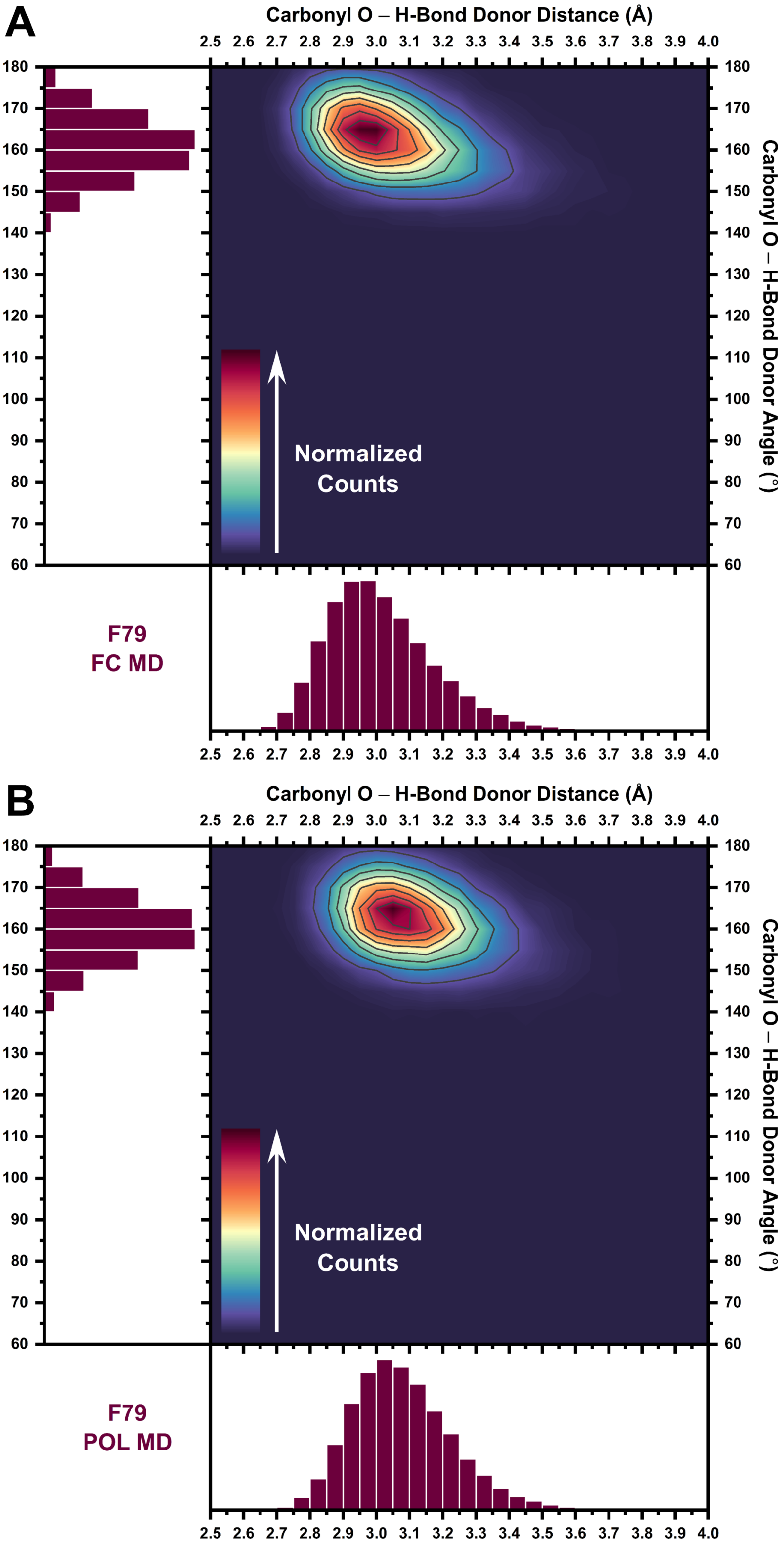


**Figure S35.** Carbonyl H-bond angle vs distance for F79 H-bonds with alpha helix H-bonding partner V83 depicted as a contour plot describing H-bond angle/distance sampling space. 1D histograms show the sampling space projected along the individual variables.

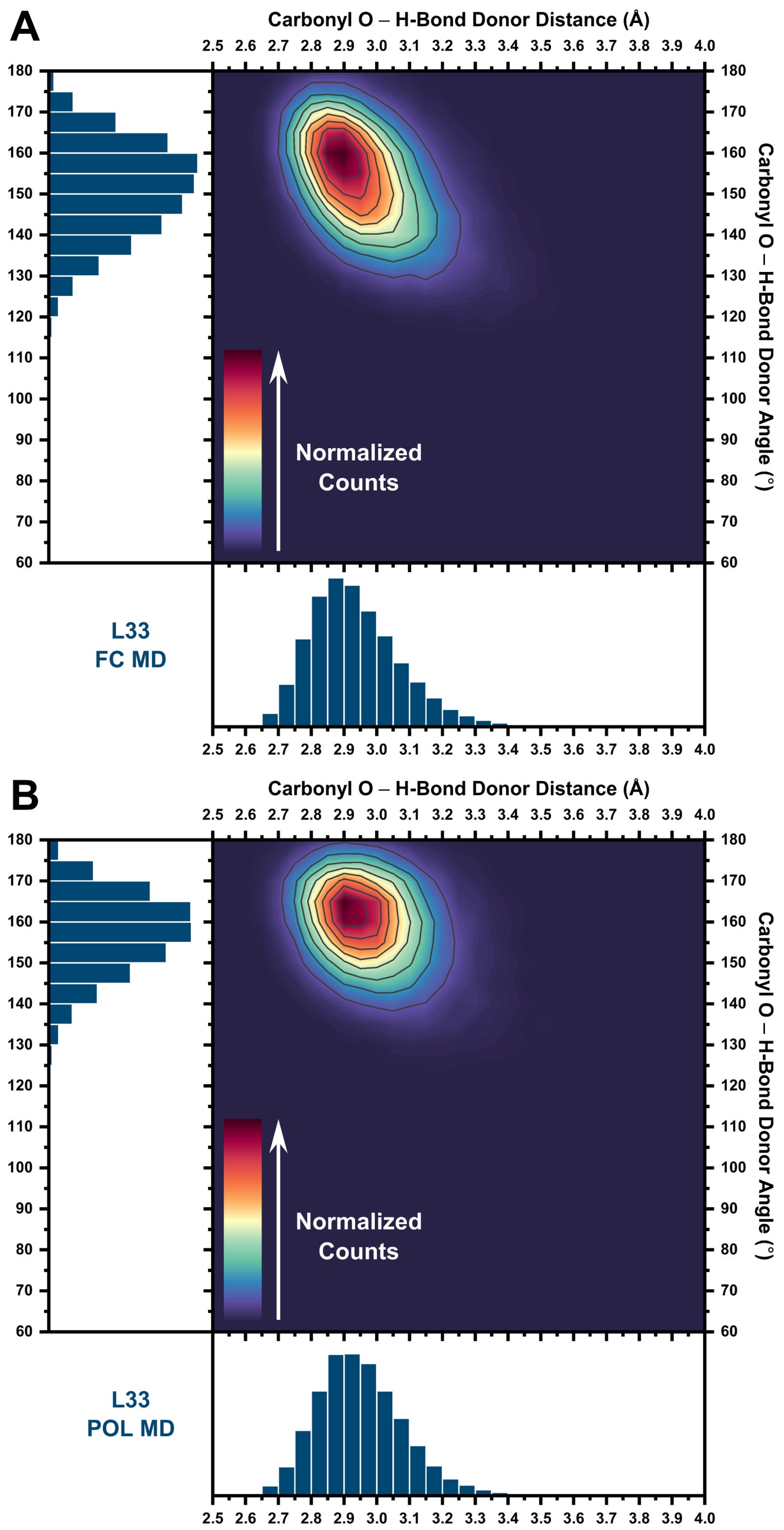


**Figure S36.** Carbonyl H-bond angle vs distance for L33 H-bonds with beta sheet H-bonding partner Y118 depicted as a contour plot describing H-bond angle/distance sampling space. 1D histograms show the sampling space projected along the individual variables.

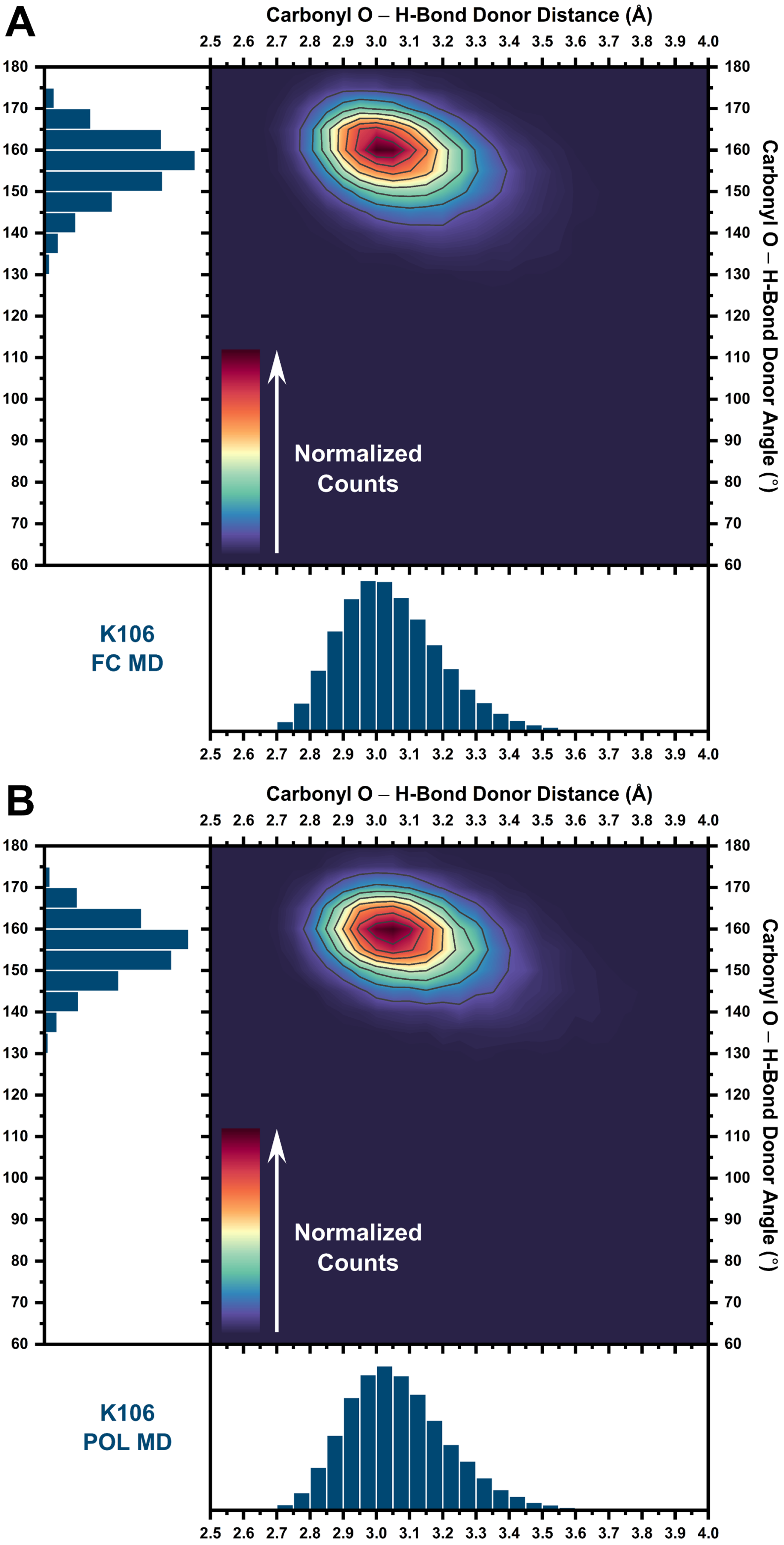


**Figure S37.** Carbonyl H-bond angle vs distance for K106 H-bonds with beta sheet H-bonding partner K123 depicted as a contour plot describing H-bond angle/distance sampling space. 1D histograms show the sampling space projected along the individual variables.

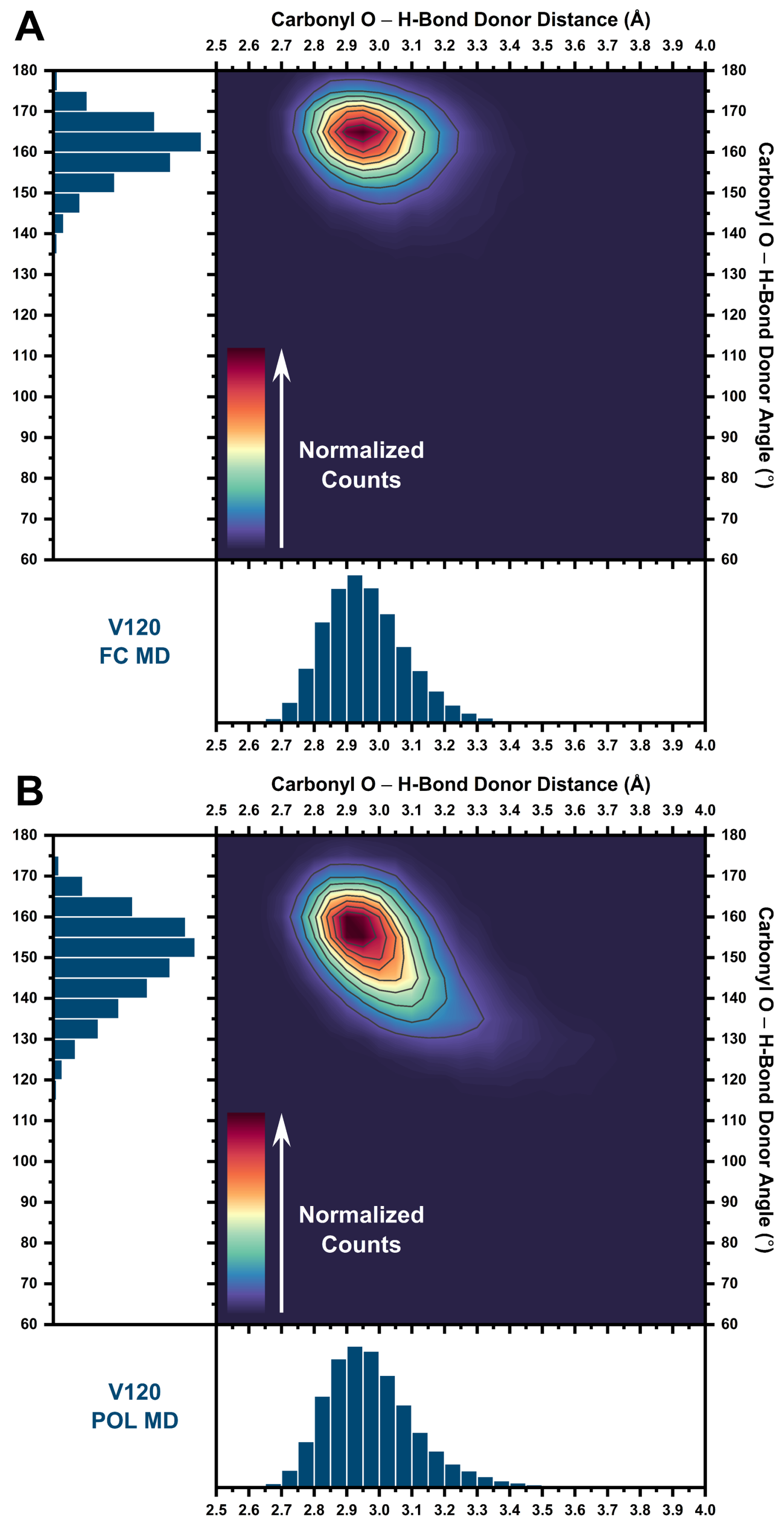


**Figure S38.** Carbonyl H-bond angle vs distance for V120 H-bonds with beta sheet H-bonding partner I31 depicted as a contour plot describing H-bond angle/distance sampling space. 1D histograms show the sampling space projected along the individual variables.

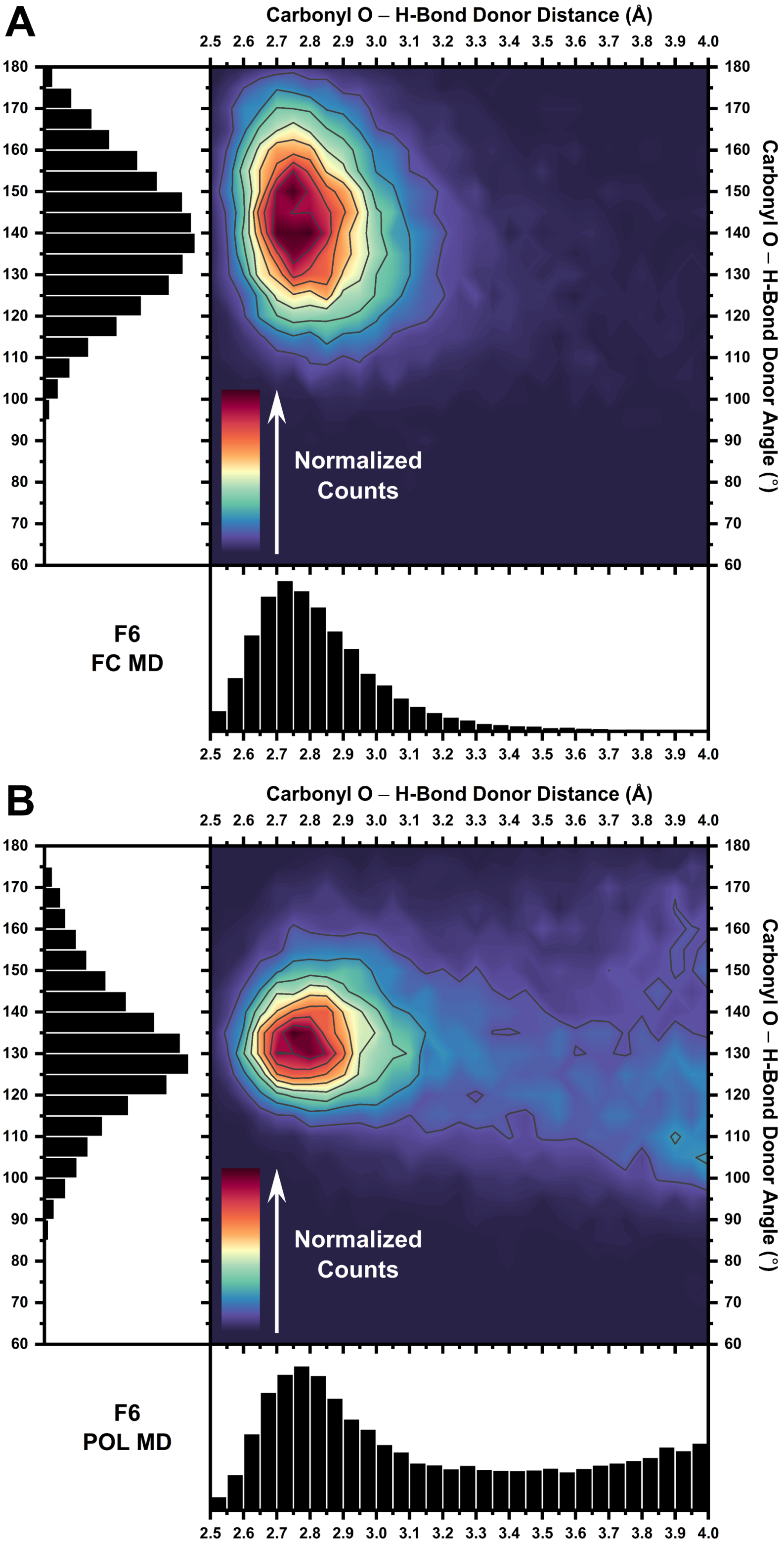


**Figure S39.** Carbonyl H-bond angle vs distance for F6 H-bonds with water depicted as a contour plot describing H-bond angle/distance sampling space. 1D histograms show the sampling space projected along the individual variables.

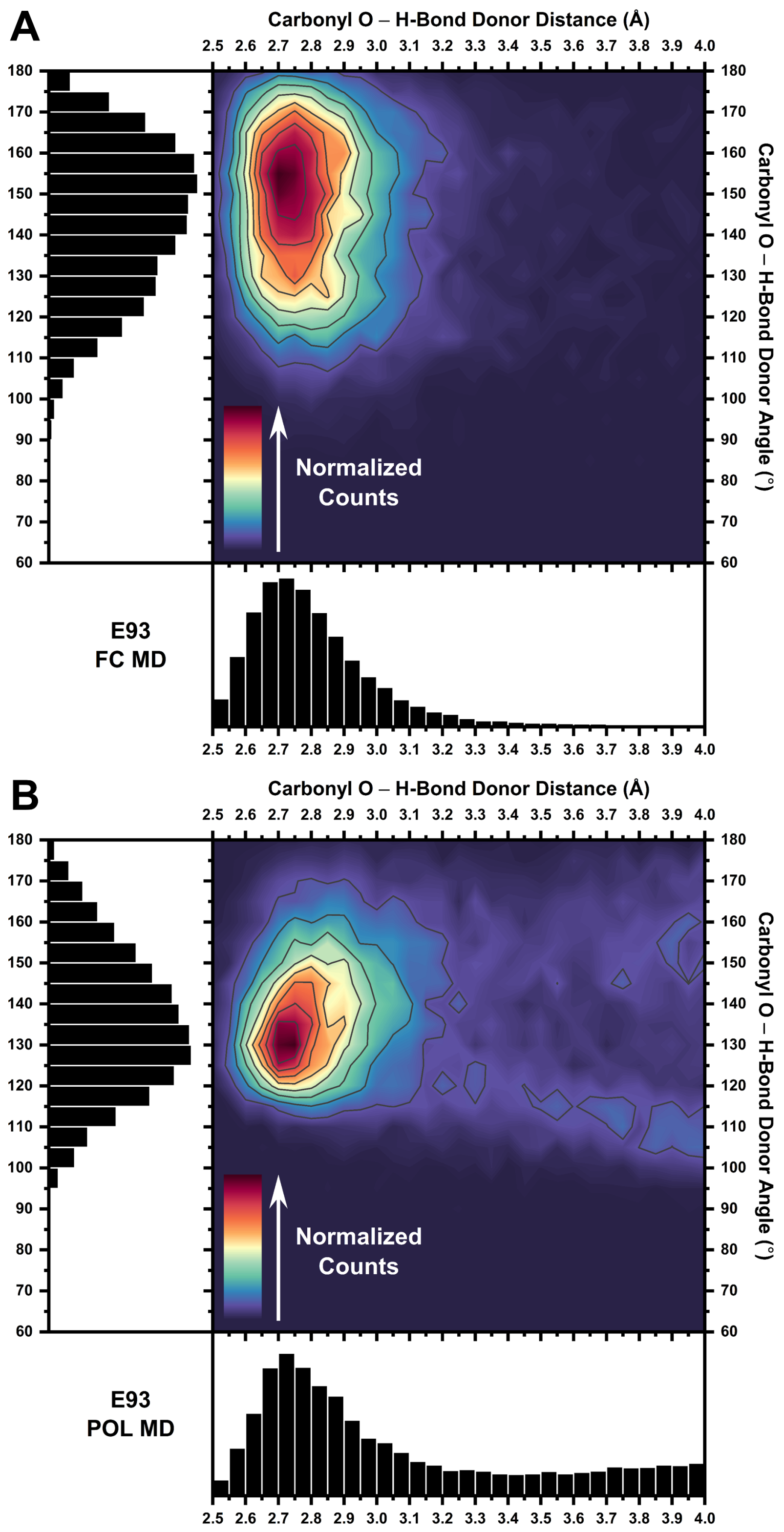


**Figure S40.** Carbonyl H-bond angle vs distance for E93 H-bonds with water depicted as a contour plot describing H-bond angle/distance sampling space. 1D histograms show the sampling space projected along the individual variables.

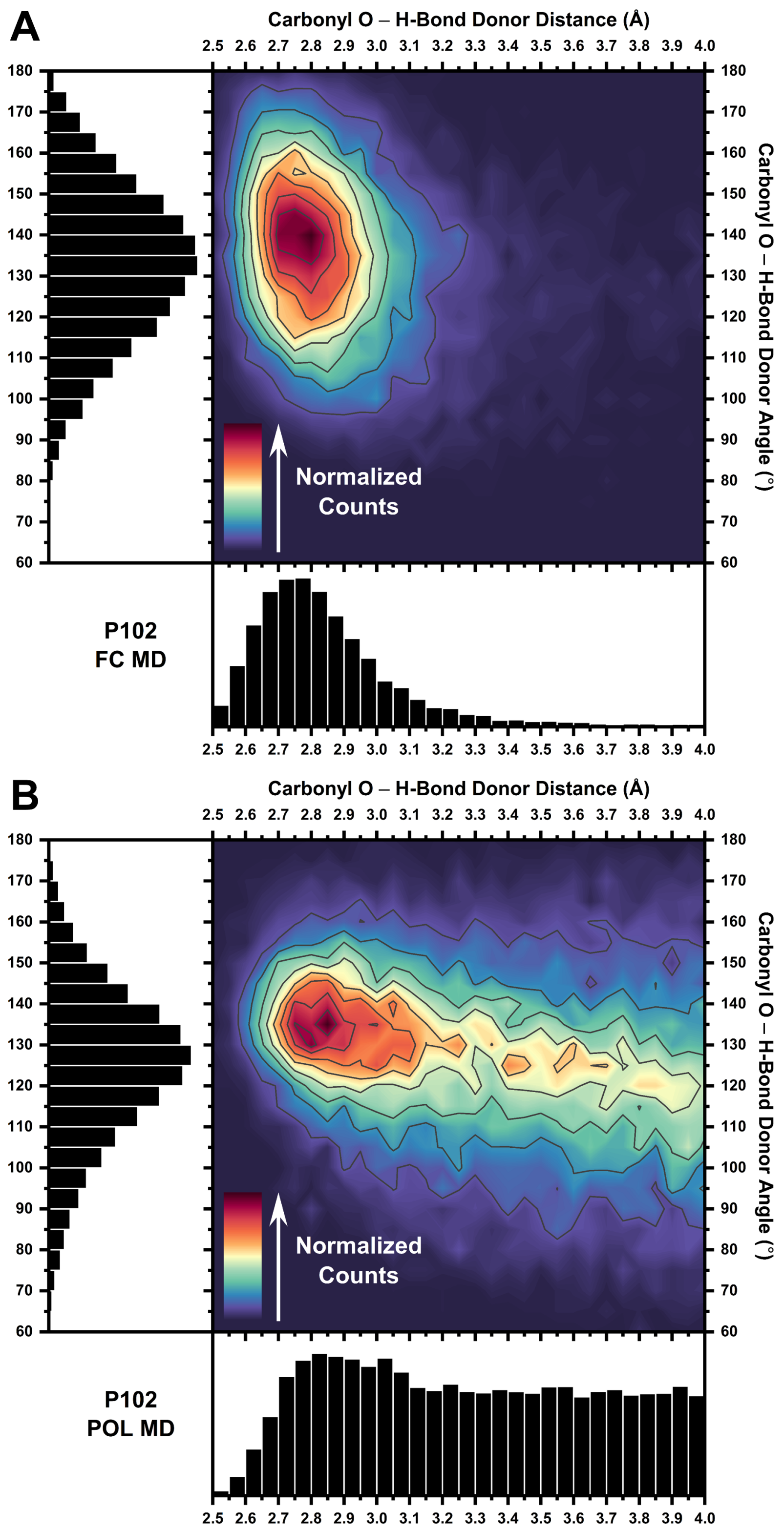


**Figure S41.** Carbonyl H-bond angle vs distance for P102 H-bonds with water depicted as a contour plot describing H-bond angle/distance sampling space. 1D histograms show the sampling space projected along the individual variables.

*Carbonyl H-bond Distances and Angles Extracted from Contour Plots*

**Table S16.** Average H-bond distances and angles for carbonyl H-bonds fit with rotated Gaussian surface. Errors provided are 1σ standard errors of the fit.

| Environment/ Force Field | Avg. H-Bond Distance ($\mu_{d_{OX}}$; Å) | Avg. H-Bond Angle ($\mu_{\theta_{COX}}$; °) | H-Bond Distance s.d. ($\sigma_{d_{OX}}$; Å) | H-Bond Angle s.d. ($\sigma_{\theta_{COX}}$; °) | $R^2$ |
|---|---|---|---|---|---|
| I11/FC | 2.99 ± 0.001 | 162.4 ± 0.06 | 0.14 ± 0.0009 | 8.8 ± 0.06 | 0.98 |
| I11/POL | 3.09 ± 0.0008 | 156.7 ± 0.04 | 0.14 ± 0.0007 | 8.1 ± 0.04 | 0.99 |
| D20/FC | 3.09 ± 0.002 | 152.3 ± 0.10 | 0.19 ± 0.002 | 10.2 ± 0.10 | 0.95 |
| D20/POL | 3.22 ± 0.002 | 145.6 ± 0.07 | 0.20 ± 0.002 | 8.9 ± 0.07 | 0.97 |
| A45/FC | 3.09 ± 0.003 | 156.4 ± 0.16 | 0.18 ± 0.002 | 10.5 ± 0.16 | 0.90 |
| A45/POL | 3.43 ± 0.002 | 146.1 ± 0.06 | 0.20 ± 0.001 | 10.7 ± 0.06 | 0.99 |
| F79/FC | 3.00 ± 0.001 | 163.6 ± 0.05 | 0.14 ± 0.0009 | 7.5 ± 0.05 | 0.98 |
| F79/POL | 3.08 ± 0.0007 | 162.8 ± 0.04 | 0.14 ± 0.0007 | 7.4 ± 0.04 | 0.99 |
| L33/FC | 2.93 ± 0.001 | 155.2 ± 0.09 | 0.11 ± 0.0009 | 12.0 ± 0.09 | 0.97 |
| L33/POL | 2.96 ± 0.0007 | 160.9 ± 0.06 | 0.12 ± 0.0007 | 9.8 ± 0.06 | 0.98 |
| K106/FC | 3.04 ± 0.0009 | 159.6 ± 0.04 | 0.14 ± 0.0008 | 7.3 ± 0.04 | 0.99 |
| K106/POL | 3.06 ± 0.0008 | 158.5 ± 0.04 | 0.14 ± 0.0007 | 7.1 ± 0.04 | 0.99 |
| V120/FC | 2.96 ± 0.0006 | 164.0 ± 0.09 | 0.12 ± 0.0006 | 6.1 ± 0.04 | 0.99 |
| V120/POL | 2.97 ± 0.001 | 154.2 ± 0.09 | 0.11 ± 0.0009 | 10.1 ± 0.09 | 0.97 |
| F6/FC | 2.79 ± 0.001 | 143.0 ± 0.2 | 0.15 ± 0.001 | 17.2 ± 0.15 | 0.96 |
| F6/POL | 2.82 ± 0.004 | 133.3 ± 0.3 | 0.17 ± 0.004 | 10.9 ± 0.3 | 0.75 |
| E93/FC | 2.77 ± 0.001 | 148.8 ± 0.2 | 0.14 ± 0.001 | 20.8 ± 0.2 | 0.95 |
| E93/POL | 2.79 ± 0.003 | 135.9 ± 0.3 | 0.15 ± 0.003 | 14.1 ± 0.3 | 0.80 |
| P102/FC | 2.80 ± 0.001 | 136.8 ± 0.2 | 0.15 ± 0.001 | 19.6 ± 0.2 | 0.96 |
| P102/POL[a] | – | – | – | – | – |

[a] A converged fit could not be obtained

## S10 Assessment of the Variants' Structural Stability During MD Simulations

We sought to assess whether our MD simulations were sufficiently converged to justify our conclusions in the main text. Fortunately, PYP is a highly characterized model photoreceptor, and its ground state dynamics have been previously characterized by NMR,[47] allowing us to estimate the necessary simulation time to sample the ground state. Analysis of the *J* coupling constants for the backbone nitrogens suggested "the absence of millisecond to microsecond time scale motions" and the presence of "internal motions in the nanosecond to picosecond time scale."[47] This study indicates PYP *does not* exhibit large conformational changes in its ground state, suggesting our simulations of at least 100 ns per variant per FF should sufficiently sample PYP's ground state. With that knowledge in hand, we also wanted to assess the stability and dynamics of the variants during the MD simulations.

To make this assessment, we obtained the average structures for F28oCNF PYP from its aggregate MD trajectories (100 ns or 200 ns for POL and FC MD, respectively; a single PYP variant was chosen as a representative since the nitrile introduction does not affect the global protein dynamics). Average structures were obtained by aligning and averaging all frames throughout the trajectories. **Figure S42A** shows the overlay of the backbones for a WT PYP crystal structure and the average structures for F28oCNF PYP with FC and POL MD. The backbones show a high degree of similarity, with root mean square deviations of 0.65 Å and 0.64 Å for the WT PYP crystal structure with F28oCNF PYP FC MD or POL MD, respectively. These values indicate the protein backbone topology is well-maintained for simulations reaching out to the hundreds of nanoseconds timescale, confirming the overall protein stability in our simulations.

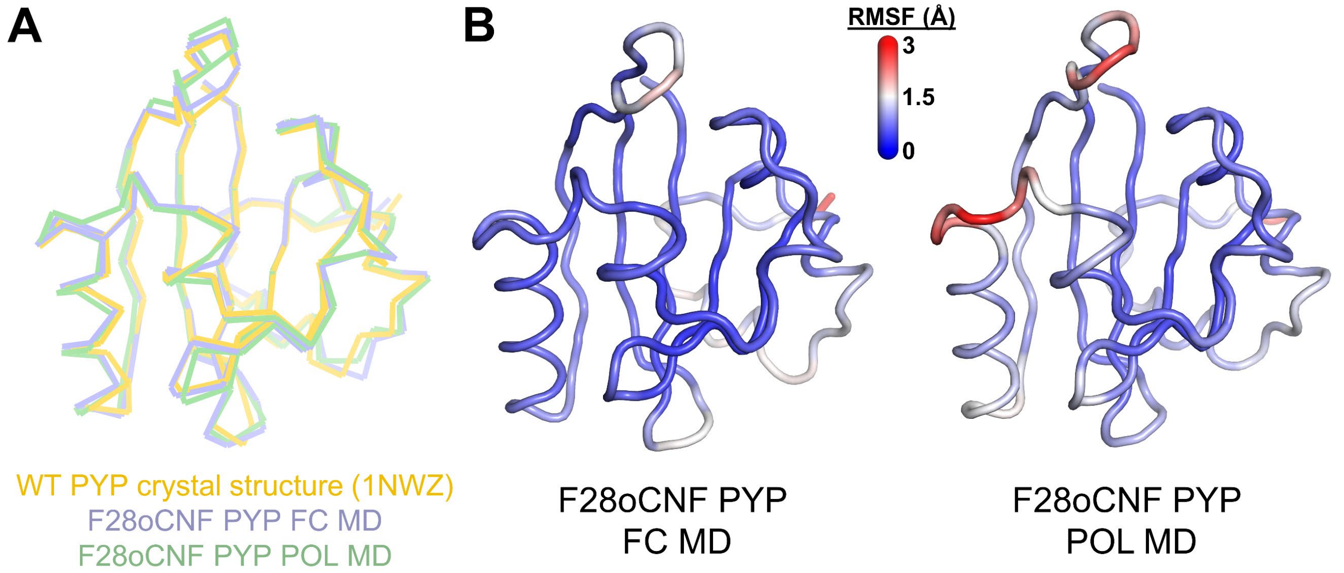


**Figure S42.** (A) Overlay of WT PYP crystal structure with average structures of F28oCNF PYP from FC and POL MD. (B) F28oCNF FC and POL MD average structures with residues colored by their $C_\alpha$'s RMSF. The color scheme runs from blue (0 Å) to white (1.5 Å) to red (3 Å).

To assess whether any large conformational changes may have occurred, we colored the F28oCNF PYP FC and POL MD average structures by the root mean square fluctuation (RMSF) for each $C_\alpha$ (**Figure S42B**). The color scheme runs from blue (0 Å) to white (1.5 Å) to red (3 Å); for reference, 3 Å is approximately the length of two C–C single bonds. In considering the RMSFs, aside from the N-terminal methionine in both FC and POL MD (4.0 Å

and 4.8 Å, respectively), no residue's RMSF was larger than 2.9 Å (E2 in POL MD). Approximately 92% and 89% of residues had RMSFs < 1.5 Å in FC and POL MD, respectively, which is readily observed in **Figure S42B** by the dearth of red coloring. Overall, the proteins demonstrated remarkable rigidity with average/standard deviation $C_\alpha$ RMSFs of 0.8/0.5 Å and 1.0/0.6 Å for FC and POL MD, respectively; the medians similarly reflect the rigidity (0.6 and 0.8 Å for FC and POL MD, respectively). Given that a C–H bond is approximately 1.0 Å, the scale of the RMSFs in the simulations implies the protein's (ground state) dynamics are confined to local fluctuations about the mean backbone position and concomitantly implies no large conformational changes occur during the simulations.

Our findings in **Figure S42** demonstrate (i) the overall protein structures were stable throughout the MD simulations and (ii) PYP does not exhibit any large conformational changes out to hundreds of nanoseconds; this second point is consistent with precedent experimental characterization.[47] Therefore, we believe the sampling performed in our MD simulations is converged such that it represents all relevant electrostatic interactions to justify the claims made in our manuscript.

## S11 References


(1) Weaver, J. B.; Kozuch, J.; Kirsh, J. M.; Boxer, S. G. Nitrile Infrared Intensities Characterize Electric Fields and Hydrogen Bonding in Protic, Aprotic, and Protein Environments. *J. Am. Chem. Soc.* **2022**, *144* (17), 7562–7567.

(2) Kozuch, J.; Schneider, S. H.; Zheng, C.; Ji, Z.; Bradshaw, R. T.; Boxer, S. G. Testing the Limitations of MD-Based Local Electric Fields Using the Vibrational Stark Effect in Solution: Penicillin G as a Test Case. *J. Phys. Chem. B* **2021**, *125* (17), 4415–4427.

(3) Schneider, S. H.; Kozuch, J.; Boxer, S. G. The Interplay of Electrostatics and Chemical Positioning in the Evolution of Antibiotic Resistance in TEM β-Lactamases. *ACS Cent Sci* **2021**, *7* (12), 1996–2008.

(4) Ji, Z.; Kozuch, J.; Mathews, I. I.; Diercks, C. S.; Shamsudin, Y.; Schulz, M. A.; Boxer, S. G. Protein Electric Fields Enable Faster and Longer-Lasting Covalent Inhibition of β-Lactamases. *J. Am. Chem. Soc.* **2022**, *144* (45), 20947–20954.

(5) Lindorff-Larsen, K.; Piana, S.; Palmo, K.; Maragakis, P.; Klepeis, J. L.; Dror, R. O.; Shaw, D. E. Improved Side-Chain Torsion Potentials for the Amber ff99SB Protein Force Field. *Proteins: Structure, Function, and Bioinformatics* **2010**, *78* (8), 1950–1958.

(6) Abraham, M. J.; Murtola, T.; Schulz, R.; Páll, S.; Smith, J. C.; Hess, B.; Lindahl, E. GROMACS: High Performance Molecular Simulations through Multi-Level Parallelism from Laptops to Supercomputers. *SoftwareX* **2015**, *1–2*, 19–25.

(7) Tinker9: Next Generation of Tinker with GPU Support. Zhi Wang, Washington University in St. Louis, 2021, Https://Github.Com/TinkerTools/Tinker9.

(8) Shi, Y.; Xia, Z.; Zhang, J.; Best, R.; Wu, C.; Ponder, J. W.; Ren, P. Polarizable Atomic Multipole-Based AMOEBA Force Field for Proteins. *J. Chem. Theory Comput.* **2013**, *9* (9), 4046–4063.

(9) Van Beeumen, J. J.; Devreese, B. V.; Van Bun, S. M.; Hoff, W. D.; Hellingwerf, K. J.; Meyer, T. E.; Cusanovich, M. A.; Mcree, D. E. Primary structure of a photoactive yellow protein from the phototrophic bacterium Ectothiorhodospira halophila, with evidence for the mass and the binding site of the chromophore. *Protein Science* **1993**, *2* (7), 1114–1125.

(10) Hoff, W. D.; Dux, P.; Hard, K.; Devreese, B.; Nugteren-Roodzant, I. M.; Crielaard, W.; Boelens, R.; Kaptein, R.; Van Beeumen, J.; Hellingwerf, K. J. Thiol Ester-Linked p-Coumaric Acid as a New Photoactive Prosthetic Group in a Protein with Rhodopsin-like Photochemistry. *Biochemistry* **1994**, *33* (47), 13959–13962.

(11) D.A. Case, I.Y. Ben-Shalom, S.R. Brozell, D.S. Cerutti, T.E. Cheatham, III, V.W.D. Cruzeiro, T.A. Darden,; R.E. Duke, D. Ghoreishi, M.K. Gilson, H. Gohlke, A.W. Goetz, D. Greene, R Harris, N. Homeyer, Y. Huang,; S. Izadi, A. Kovalenko, T. Kurtzman, T.S. Lee, S. LeGrand, P. Li, C. Lin, J. Liu, T. Luchko, R. Luo, D.J.; Mermelstein, K.M. Merz, Y. Miao, G. Monard, C. Nguyen, H. Nguyen, I. Omelyan, A. Onufriev, F. Pan, R.; Qi, D.R. Roe, A. Roitberg, C. Sagui, S. Schott-Verdugo, J. Shen, C.L. Simmerling, J. Smith, R. SalomonFerrer, J. Swails, R.C. Walker, J. Wang, H. Wei, R.M. Wolf, X. Wu, L. Xiao, D.M. York and P.A. Kollman. AMBER 2018, 2018.

(12) Wang, J.; Wolf, R. M.; Caldwell, J. W.; Kollman, P. A.; Case, D. A. Development and Testing of a General Amber Force Field. *Journal of Computational Chemistry* **2004**, *25* (9), 1157–1174.

(13) Frisch, M. J.; Trucks, G. W.; Schlegel, H. B.; Scuseria, G. E.; Robb, M. A.; Cheeseman, J. R.; Scalmani, G.; Barone, V.; Petersson, G. A.; Nakatsuji, H., et al., Gaussian 16, Rev A.03., Gaussian, Inc.: Wallingford, CT, 2016.

(14) Walker, B.; Liu, C.; Wait, E.; Ren, P. Automation of AMOEBA Polarizable Force Field for Small Molecules: Poltype 2. *Journal of Computational Chemistry* **2022**, *43* (23), 1530–1542.

(15) Rackers, J. A.; Wang, Z.; Lu, C.; Laury, M. L.; Lagardère, L.; Schnieders, M. J.; Piquemal, J.-P.; Ren, P.; Ponder, J. W. Tinker 8: Software Tools for Molecular Design. *J. Chem. Theory Comput.* **2018**, *14* (10), 5273–5289.

(16) Fried, S. D.; Wang, L.-P.; Boxer, S. G.; Ren, P.; Pande, V. S. Calculations of the Electric Fields in Liquid Solutions. *The Journal of Physical Chemistry B* **2013**, *117* (50), 16236–16248.

(17) Fried, S. D.; Boxer, S. G. Measuring Electric Fields and Noncovalent Interactions Using the Vibrational Stark Effect. *Acc. Chem. Res.* **2015**, *48* (4), 998–1006.
(18) Fried, S. D. E.; Zheng, C.; Mao, Y.; Markland, T. E.; Boxer, S. G. Solvent Organization and Electrostatics Tuned by Solute Electronic Structure: Amide versus Non-Amide Carbonyls. *J. Phys. Chem. B* **2022**, *126* (31), 5876–5886.
(19) Pang, Y.-P. Use of 1–4 Interaction Scaling Factors to Control the Conformational Equilibrium between α-Helix and β-Strand. *Biochemical and Biophysical Research Communications* **2015**, *457* (2), 183–186.
(20) Chung, J. K.; Thielges, M. C.; Bowman, S. E. J.; Bren, K. L.; Fayer, M. D. Temperature Dependent Equilibrium Native to Unfolded Protein Dynamics and Properties Observed with IR Absorption and 2D IR Vibrational Echo Experiments. *J. Am. Chem. Soc.* **2011**, *133* (17), 6681–6691.
(21) Bradshaw, R. T.; Dziedzic, J.; Skylaris, C.-K.; Essex, J. W. The Role of Electrostatics in Enzymes: Do Biomolecular Force Fields Reflect Protein Electric Fields? *J. Chem. Inf. Model.* **2020**, *60* (6), 3131–3144.
(22) Le Questel, J.-Y.; Berthelot, M.; Laurence, C. Hydrogen-Bond Acceptor Properties of Nitriles: A Combined Crystallographic and Ab Initio Theoretical Investigation. *Journal of Physical Organic Chemistry* **2000**, *13* (6), 347–358.
(23) Rani, P.; Biswas, P. Shape Dependence of the Radial Distribution Function of Hydration Water around Proteins. *J. Phys.: Condens. Matter* **2014**, *26* (33), 335102.
(24) Tan, K. P.; Singh, K.; Hazra, A.; Madhusudhan, M. S. Peptide Bond Planarity Constrains Hydrogen Bond Geometry and Influences Secondary Structure Conformations. *Curr Res Struct Biol* **2020**, *3*, 1–8.
(25) Kuroda, D. G.; Bauman, J. D.; Challa, J. R.; Patel, D.; Troxler, T.; Das, K.; Arnold, E.; Hochstrasser, R. M. Snapshot of the Equilibrium Dynamics of a Drug Bound to HIV-1 Reverse Transcriptase. *Nature Chem* **2013**, *5* (3), 174–181.
(26) Lin, Y.-C.; Ren, P.; Webb, L. J. AMOEBA Force Field Predicts Accurate Hydrogen Bond Counts of Nitriles in SNase by Revealing Water–Protein Interaction in Vibrational Absorption Frequencies. *J. Phys. Chem. B* **2023**, *127* (25), 5609–5619.
(27) Li, Y.; Natakorn, S.; Chen, Y.; Safar, M.; Cunningham, M.; Tian, J.; Li, D. D.-U. Investigations on Average Fluorescence Lifetimes for Visualizing Multi-Exponential Decays. *Frontiers in Physics* **2020**, *8:5768628*.
(28) Sepulveda-Montaño, L. X.; Galindo, J. F.; Kuroda, D. G. Infrared Spectroscopy of Liquid Solutions as a Benchmarking Tool of Semiempirical QM Methods: The Case of GFN2-xTB. *J. Phys. Chem. B* **2023**, *127* (37), 7955–7963.
(29) Kim, Y. S.; Hochstrasser, R. M. Chemical Exchange 2D IR of Hydrogen-Bond Making and Breaking. *Proceedings of the National Academy of Sciences* **2005**, *102* (32), 11185–11190.
(30) 2D IR Photon Echo Spectroscopy Reveals Hydrogen Bond Dynamics of Aromatic Nitriles. *Chemical Physics Letters* **2009**, *469* (4–6), 325–330.
(31) Rodgers, J. M.; Zhang, W.; Bazewicz, C. G.; Chen, J.; Brewer, S. H.; Gai, F. Kinetic Isotope Effect Provides Insight into the Vibrational Relaxation Mechanism of Aromatic Molecules: Application to Cyano-Phenylalanine. *J. Phys. Chem. Lett.* **2016**, *7* (7), 1281–1287.
(32) Bagchi, S.; Boxer, S. G.; Fayer, M. D. Ribonuclease S Dynamics Measured Using a Nitrile Label with 2D IR Vibrational Echo Spectroscopy. *J. Phys. Chem. B* **2012**, *116* (13), 4034–4042.
(33) Bryant, R. G. The NMR Time Scale. *J. Chem. Educ.* **1983**, *60* (11), 933.
(34) Kleckner, I. R.; Foster, M. P. An Introduction to NMR-Based Approaches for Measuring Protein Dynamics. *Biochim Biophys Acta* **2011**, *1814* (8), 942–968.
(35) Lee, H.; Choi, J.-H.; Cho, M. Vibrational Solvatochromism and Electrochromism. II. Multipole Analysis. *The Journal of Chemical Physics* **2012**, *137* (11), 114307.
(36) Xu, L.; Cohen, A. E.; Boxer, S. G. Electrostatic Fields near the Active Site of Human Aldose Reductase: 2. New Inhibitors and Complications Caused by Hydrogen Bonds. *Biochemistry* **2011**, *50* (39), 8311–8322.
(37) Huang, C.-Y.; Wang, T.; Gai, F. Temperature Dependence of the CN Stretching Vibration of a Nitrile-Derivatized Phenylalanine in Water. *Chemical Physics Letters* **2003**, *371* (5), 731–738.

(38) Kim, Y. S.; Hochstrasser, R. M. Comparison of Linear and 2D IR Spectra in the Presence of Fast Exchange. *J. Phys. Chem. B* **2006**, *110* (17), 8531–8534.
(39) Boxer, S. G. Stark Realities. *J. Phys. Chem. B* **2009**, *113* (10), 2972–2983.
(40) York, D.; Evensen, N. M.; Martínez, M. L.; De Basabe Delgado, J. Unified Equations for the Slope, Intercept, and Standard Errors of the Best Straight Line. *American Journal of Physics* **2004**, *72* (3), 367–375.
(41) Travis Wiens (2023). Linear Regression with Errors in X and Y (https://www.mathworks.com/matlabcentral/fileexchange/26586-linear-regression-with-errors-in-x-and-y), MATLAB Central File Exchange. Retrieved September 30, 2023.
(42) Welborn, V. V.; Head-Gordon, T. Fluctuations of Electric Fields in the Active Site of the Enzyme Ketosteroid Isomerase. *J. Am. Chem. Soc.* **2019**, *141* (32), 12487–12492.
(43) Wang, X.; He, X. An Ab Initio QM/MM Study of the Electrostatic Contribution to Catalysis in the Active Site of Ketosteroid Isomerase. *Molecules* **2018**, *23* (10), 2410.
(44) Richard, A. M.; Gascón, J. A. Protein Polarization Effects in the Thermodynamic Computation of Vibrational Stark Shifts. *Theor Chem Acc* **2019**, *139* (1), 9.
(45) Zheng, C.; Mao, Y.; Kozuch, J.; Atsango, A. O.; Ji, Z.; Markland, T. E.; Boxer, S. G. A Two-Directional Vibrational Probe Reveals Different Electric Field Orientations in Solution and an Enzyme Active Site. *Nat. Chem.* **2022**, *14* (8), 891–897.
(46) Izadi, S.; Anandakrishnan, R.; Onufriev, A. V. Building Water Models: A Different Approach. *J. Phys. Chem. Lett.* **2014**, *5* (21), 3863–3871.
(47) Düx, P.; Rubinstenn, G.; Vuister, G. W.; Boelens, R.; Mulder, F. A. A.; Hård, K.; Hoff, W. D.; Kroon, A. R.; Crielaard, W.; Hellingwerf, K. J.; Kaptein, R. Solution Structure and Backbone Dynamics of the Photoactive Yellow Protein. *Biochemistry* **1998**, *37* (37), 12689–12699.
